# Screen time and autism like behavior: Cross-sectional study from Georgia

**DOI:** 10.1016/j.pmedr.2026.103503

**Published:** 2026-05-22

**Authors:** Konstantine Chakhunashvili, Davit G. Chakhunashvili, Chidhambara Krishna Muthuvelayutham Sangaranachiar, Shri Subaa Mathy Muthuvelayutham Sangaranachiar

**Affiliations:** a Caucasus University, Caucasus Medicine School, Associate Professor, Tbilisi, Georgia. D. Abuladze Georgian-Italian Clinic, Pediatrician, Tbilisi, Georgia; b Alte University, International School of Medicine, Associate Professor, Tbilisi, Georgia. D. Abuladze Georgian-Italian Clinic, Pediatrician, Tbilisi, Georgia; c Caucasus University, Tbilisi, Georgia

**Keywords:** Screen time, M-CHAT-R/F, Autism

## Abstract

**Objectives:**

Early childhood is a critical neurodevelopmental period during which environmental exposures may influence cognitive, language, and social outcomes. Screen media use has become increasingly prevalent in infancy and toddlerhood, yet evidence regarding its association with autism screening outcomes remains inconsistent.

**Methods:**

A cross-sectional observational study was conducted among children aged 16–30 months. Surveys were completed by primary caregivers between April 2025 and January 2026 through in-clinic recruitment and online distribution. Autism screening was performed using the Modified Checklist for Autism in Toddlers, Revised with Follow-Up (M-CHAT-R/F). All variables were parent-reported.

**Results:**

Higher average daily screen time at assessment and over the preceding six and twelve months was significantly associated with higher M-CHAT-R/F scores. Screen exposure before 12 months of age was associated with a greater likelihood of moderate- and high-risk screening classifications. In multivariate models, earlier exposure and longer screen time duration remained significant positive predictors of M-CHAT-R/F scores.

**Conclusion:**

Both the timing and duration of screen exposure were associated with increased autism screening risk scores. Although causal inferences cannot be made and effect sizes were small to moderate, the findings support current pediatric recommendations discouraging screen exposure during infancy.

## Highlights

• Screen exposure before 12 months is linked to higher autism screening scores.
• Infant screen use increases odds of moderate/high-risk M-CHAT-R/F scores.
• Associations persisted after socioeconomic and demographic adjustment.
• The first year is a sensitive neurodevelopmental exposure window.
• Delaying infant screen exposure may lower medium/high-risk M-CHAT-R/F scores.

## 1 Introduction

Early childhood is a sensitive period for neurodevelopment, and screen media exposure now begins increasingly early despite recommendations to limit it in the first years of life (Council on Communications and Media, 2016; Sarfraz et al., 2023a; Chakhunashvili et al., 2024). This has raised concern that early and sustained exposure may affect cognitive, language, and social development.

The Modified Checklist for Autism in Toddlers, Revised with Follow-Up (M-CHAT-R/F), is widely used to identify children aged 16–30 months who may need further autism evaluation (Robins et al., 2014). Prior studies have linked screen exposure with higher autism-related screening scores, but results remain inconsistent and are often limited by small samples or incomplete adjustment for sociodemographic factors (Ophir et al., 2023a).

Recent work suggests that both exposure timing and duration may matter, with infancy potentially representing a particularly sensitive window (Sarfraz et al., 2023a; Chakhunashvili et al., 2024; Ophir et al., 2023a; Chakhunashvili and Chakhunashvili, 2025). Nevertheless, limited exposure characterization and residual confounding have constrained causal interpretation.

Building on this literature, the present study aimed to examine these associations in a Georgian sample, a setting underrepresented in the existing literature, while simultaneously considering both timing and duration of exposure. We assessed current screen time as well as average exposure over the preceding six and twelve months, retrospectively, alongside sociodemographic variables selected because of their documented links with media use patterns, developmental opportunity, and access to health resources. We hypothesized that earlier screen exposure and greater cumulative screen time would be associated with higher M-CHAT-R/F scores and screening risk categories, independent of these covariates.

## 2 Methods

### 2.1 Study design and population

This cross-sectional observational study initially received 2669 survey submissions; after removal of duplicate responses identified by the parents cell phone number and e-mail, 1834 unique parent-reported records were retained for analysis. Out of the duplicate responses the first to be submitted was left. Eligible children were aged 16–30 months. Data were collected between April 2025 and January 2026 through a combination of in-clinic recruitment and online distribution. In-person data collection was conducted at two pediatric centers in Georgia. Parents were approached during routine pediatric visits or could access the same structured self-administered questionnaire online. Recruitment-source-specific refusal rates and source-stratified response counts were not recorded and are therefore not available for analysis. The survey targeted caregivers living in Georgia; emigrant responses were retained as a separate residence category when reported.

### 2.2 Measures

Autism risk was assessed using the Modified Checklist for Autism in Toddlers, Revised with Follow-Up (M-CHAT-R/F), a 20-item parent-completed screening tool for children aged 16–30 months (Robins et al., 2014). Items assess early social-communication and related behavioral features indicitave of autism risk. The total score was analyzed both as a continuous variable and as a categorical outcome, with children classified into low- (0–2), moderate- (3–7), and high-risk (8–20) groups according to established scoring criteria (Robins et al., 2014). Higher scores indicated greater screening risk rather than a clinical diagnosis.

Parents were instructed to report active screen exposure only, defined as time during which the child directly attended to screen-based content on digital devices (e.g. TV, smart phone, tablet, laptop, desktop computer, gaming consoles). Background media exposure (e.g., television on in the room) and video calls involving caregivers were excluded. Screen exposure variables were derived from parent-reported estimates as average daily screen time in minutes per day, including current exposure at survey completion and average exposure over the preceding six and twelve months; all measures were treated as estimates rather than precise values. These retrospective exposure windows were intended to capture earlier typical exposure and should not be interpreted as longitudinal trajectories. The questionnaire was study-specific rather than based on a previously validated screen-time instrument, and content type and caregiver-mediated versus solitary screen use could not be distinguished. Age at onset of screen exposure was categorized as before 6 months, 6 to 12 months, 12 to 18 months, 18 to 24 months, or no exposure; for analysis, exposure before 12 months was dichotomized (yes/no).

Sociodemographic variables collected included biologic sex, place of residence (which were then categorized into city/town, village, emigrant, mountainous region), maternal and paternal education level, ethnicity, religious affiliation, and average monthly household income reported in Georgian Lari (GEL) (supplementary Table 1). These variables were included as covariates to account for socio-contextual factors that may influence children's media exposure patterns and broader developmental opportunity structures. Religious affiliation was not assumed to be a direct confounder, but was included as a proxy for cultural and behavioral factors influencing parenting practices and screen exposure, consistent with epidemiological adjustment strategies. Parental education was categorized based on the highest attained academic degree. All sociodemographic variables were treated as categorical variables rather than continuous measures and were entered into the analyses as categorical covariates.

Missing values were initially handled using pairwise deletion because overall missingness was limited (3.48%) and data availability remained high across variable pairs. To evaluate the robustness of the findings and reduce the possibility of bias related to incomplete data, multiple imputation analyses were additionally performed, as this approach is generally recommended under missing-at-random assumptions. Importantly, the primary associations of interest — particularly current screen time exposure and exposure before 12 months of age — remained directionally and statistically consistent across both approaches. Multiple imputation resulted in slightly narrower confidence intervals and several additional covariates reaching statistical significance, likely reflecting increased statistical efficiency rather than meaningful changes in effect estimates or study conclusions. Therefore, the imputed analyses were interpreted as supportive sensitivity analyses confirming the stability of the main findings.

### 2.3 Statistical analysis

Descriptive statistics summarized continuous variables as means and standard deviations and categorical variables as frequencies and percentages. Associations with M-CHAT-R/F scores were examined using correlation and chi-square analyses. Multiple linear regression and general linear models assessed independent associations with screen time across exposure periods, adjusting for sociodemographic factors. Effect sizes were reported, and a Bonferroni correction (m = 3, *p* < 0.017) was applied. Analyses were performed in SPSS 26.

## 3 Results

The study included 1834 children aged 16–30 months, with the majority classified as low risk on the M-CHAT-R/F (77.60%), followed by medium (17.60%) and high risk (4.90%) (Table 1). The sample was predominantly male (62.80%) and most patients reported residing in urban areas (74.60%). Screen exposure was common early in life, with 12.2% of children exposed before 6 months of age and nearly half (49.80%) exposed before 12 months, while 17.40% had not yet been exposed during the survey collection (Table 1). Most mothers (72.30%) and fathers (56.30%) held at least a bachelor's degree (Table 1). The population was largely ethnically Georgian (94.50%) and Orthodox Christian (93.20%) (Table 1). The mean M-CHAT-R/F score was 1.71 (Table 1). Average daily screen time was 64.10 min at assessment, increasing to 93.80 min over the past six months average and 108.40 min over the past year average (Table 2). These retrospective estimates should be interpreted cautiously because they reflect parent recall rather than within-child longitudinal change. Mean monthly household income was 3143.8 GEL (Table 2).

**Table 1:** Frequencies of categorical variables among children aged 16–30 months included in a cross-sectional screen time and autism screening study in Georgia, april 2025–january 2026.

M-CHAT-R/F Categories					
		Frequency	%	Valid %	Total %
Valid	Low Risk	1423	77.59	77.59	77.59
	Medium Risk	322	17.56	17.56	95.15
	High Risk	89	4.85	4.85	100.00
	Total	1834	100.00	100.00	

Age in months					
		Frequency	%	Valid %	Total %
Valid	16	174	9.49	9.63	9.63
	17	117	6.38	6.47	16.10
	18	205	11.18	11.34	27.45
	19	122	6.65	6.75	34.20
	20	123	6.71	6.81	41.01
	21	101	5.51	5.59	46.60
	22	91	4.96	5.04	51.63
	23	87	4.74	4.81	56.45
	24	231	12.60	12.78	69.23
	25	87	4.74	4.81	74.05
	26	75	4.09	4.15	78.20
	27	65	3.54	3.60	81.79
	28	57	3.11	3.15	84.95
	29	64	3.49	3.54	88.49
	30	208	11.34	11.51	100.00
	Total	1807	98.53	100.00	
Missing	Missing	27	1.47		
Total		1834	100.00		

Biologic Sex					
		Frequency	%	Valid %	Total %
Valid	Female	673	36.70	37.22	37.22
	Male	1135	61.89	62.78	100.00
	Total	1808	98.58	100.00	
Missing	Missing	26	1.42		
Total		1834	100.0		

Start of Screen Time Exposure					
		Frequency	%	Valid %	Total %
Valid	Exposure Before 6 Months	221	12.05	12.22	12.22
	Exposure Between 6 and 12 Months	679	37.02	37.56	49.78
	Exposure Between 12 and 18 Months	420	22.90	23.23	73.01
	Exposure Between 18 and 24 Months	173	9.43	9.57	82.58
	Not Exposed Yet	315	17.18	17.42	100.00
	Total	1808	98.58	100.00	
Missing	Missing	26	1.42		
Total		1834	100.00		

Level of Education (Mother)					
		Frequency	%	Valid %	Total %
Valid	Middle School	62	3.38	3.44	3.44
	High School	420	22.90	23.32	26.76
	Bachelor	912	49.73	50.64	77.40
	Master or Equivalent	391	21.32	21.71	99.11
	PhD	16	0.87	0.89	100.00
	Total	1801	98.20	100.00	
Missing	System	33	1.80		
Total		1834	100.00		

Level of Education (Father)					
		Frequency	%	Valid %	Total %
Valid	Middle School	90	4.91	5.00	5.00
	High School	663	36.15	36.83	41.83
	Bachelor	727	39.64	40.39	82.22
	Master or Equivalent	286	15.59	15.89	98.11
	PhD	34	1.85	1.89	100.00
	Total	1800	98.15	100.00	
Missing	Missing	34	1.85		
Total		1834	100.00		

Place of Residence					
		Frequency	%	Valid %	Total %
Valid	City/Town	1343	73.23	74.57	74.57
	Village	270	14.72	14.99	89.56
	Mountainous Region	28	1.53	1.55	91.12
	Emigrant (West)	153	8.34	8.50	99.61
	Emigrant (Asia/Russia/Africa)	7	0.38	0.39	100.00
	Total	1801	98.20	100.00	
Missing	Missing	33	1.80		
Total		1834	100.0		

Ethnicity					
		Frequency	%	Valid %	Total %
Valid	Georgian	1703	92.86	94.51	94.51
	Azerbaijani	10	0.55	0.55	95.06
	Armenian	15	0.82	0.83	95.89
	Ukrainian	5	0.27	0.28	96.17
	Russian	6	0.33	0.33	96.50
	Mixed	47	2.56	2.61	99.11
	Other	16	0.87	0.89	100.00
	Total	1802	98.26	100.00	
Missing	Missing	32	1.74		
Total		1834	100.0		

Religious Belonging of the Family					
		Frequency	Percent	Valid Percent	Cumulative Percent
Valid	Orthodox	1682	91.71	93.24	93.24
	Muslim	61	3.33	3.38	96.62
	Catholic	6	0.33	0.33	96.95
	Protestant	8	0.44	0.44	97.39
	Atheist	13	0.71	0.72	98.12
	Other	34	1.85	1.88	100.00
	Total	1804	98.36	100.00	
Missing	Missing	30	1.64		
Total		1834	100.0

**Table 2:** Descriptive statistics for numerical variables among children aged 16–30 months included in a cross-sectional screen time and autism screening study in Georgia, april 2025–january 2026.

	N	Minimum	Maximum	Mean	Std. Deviation
M-CHAT-R/F Score	1808	0	17	1.71	2.75
Average Daily Screen Time Duration (Now)	1801	0	600	64.12	77.68
Average Daily Screen Time Duration (For The Past 6 months)	1466	0	600	93.79	106.59
Average Daily Screen Time Duration (For the past year)	1458	0	800	108.42	130.94
Average Monthly Household Income (GEL)	1755	0	100,000	3143.77	3845.41
Valid N (listwise)	1421				

### 3.1 Bivariate analysis

Among continuous variables, average daily screen time at the time of survey completion, during the past six months, and during the past year showed weak but statistically significant positive correlations with M-CHAT-R/F scores (see Table 3) (Supplementary File S). In contrast, average monthly household income demonstrated a weak, statistically significant negative association with M-CHAT-R/F scores (Supplementary File S).

Among categorical variables, religious affiliation and ethnicity were not significantly associated with M-CHAT-R/F scores. Maternal education level and age at onset of screen time exposure showed moderate, statistically significant associations with M-CHAT-R/F scores (Table 3) (Supplementary File S).

Early screen exposure (before 12 months of age) was significantly associated with M-CHAT-R/F risk category, χ^2^(2) = 69.85, *p* < 0.01 (Table 4). Children exposed to screen time before 12 months were nearly twice as likely to fall into the moderate or high-risk categories. Specifically, the proportion classified as high risk was 6.55% compared with 3.30% among those exposed later (difference = 3.25%, 95% CI [1.26, 5.31], χ^2^(1) = 10.20, *p* < 0.01), while moderate risk classification was observed in 24.44% versus 11.23% (difference = 13.21%, 95% CI [9.72, 16.68], χ^2^(1) = 53.86, p < 0.01) (Supplementary File S). The odds ratios (OR) essentially showed the same results - children exposed before 12 months of age had significantly higher odds of being classified as high risk compared with those exposed later (OR = 1.98, 95% CI [1.27, 3.11],p < 0.01) and early exposure was also associated with increased odds of medium risk classification (OR = 2.18, 95% CI [1.69, 2.80], *p* < 0.01).

### 3.2 Multivariable analysis

In the multivariate analysis, separate linear regression models were fitted with M-CHAT-R/F score as the dependent variable and average daily screen time exposure (current, past 6 months, and past 12 months) together with average monthly household income as predictors. All three models were statistically significant. The model including current screen time explained 11.0% of the variance in M-CHAT-R/F scores, R^2^ = 0.11, F(2, 1750) = 105.68, *p* < 0.01. The model incorporating average screen time over the past six months explained 8.0% of the variance, R^2^ = 0.08, F(2, 1424) = 63.48, p < 0.01, while the model using average screen time over the past year explained 6.1% of the variance, R^2^ = 0.06, F(2, 1418) = 45.69, *p* < 0.01. A multiple linear regression was conducted to examine whether average daily screen time during the past 6 months and the past year predicted current average daily screen time duration. The overall model showed that both predictors were statistically significant. A multiple linear regression was conducted to examine whether average daily screen time during the past 6 months and the past year predicted current average daily screen time duration. The overall model showed that both predictors were statistically significant. Average daily screen time during the past 6 months was a strong positive predictor of current screen time, B = 0.51, SE = 0.01, β = 0.71, *t* = 51.75, *p* < 0.01, 95% CI [0.49, 0.53]. In contrast, average daily screen time during the past year demonstrated a smaller but statistically significant negative association with current screen time after adjustment for the 6-month exposure variable, B = −0.08, SE = 0.01, β = −0.14, *t* = −10.23, p < 0.01, 95% CI [−0.10, −0.07]. Collinearity diagnostics suggested acceptable but notable overlap between predictors (Tolerance = 0.325, VIF = 3.08 for both variables), indicating moderate shared variance but no evidence of severe multicollinearity according to conventional thresholds.

**Table 3:** Bivariate Relationships Between M-CHAT-R/F Scores and Sociodemographic and Screen Exposure Variables Among Children Aged 16–30 Months in Georgia, April 2025–January 2026.

	M-CHAT-R/F score
Average Daily Screen Time (At the moment of survey completion)	r(1751) = 0.32, R^2^ = 0.10, p < 0.01
Average Daily Screen Time (For the past 6 months)	r(1425) = 0.28, R^2^ = 0.08, p < 0.01
Average Daily Screen Time (For the Past Year)	r(1419) = 0.23, R^2^ = 0.05, p < 0.01
Average Monthly Household Income (Georgian Lari)	r(1751) = − 0.08, R^2^ = 0.08, p < 0.01
Biologic Sex	ηp^2^ (1) = 0.01, p < 0.01
Start of Screen Time Exposure	ηp^2^ (4) = 0.06, p < 0.01
Level of Education (Mother)	ηp^2^ (4) = 0.05, *p* < 0.01
Level of Education (Father)	ηp^2^ (4) = 0.02, *p* < 0.01
Religious belonging of the family	ηp^2^ (5) = 0.01, *p* = 0.10
Ethnicity	ηp^2^(6) = 0.01, *p* = 0.17
Place of Residence	ηp^2^ (4) = 0.01, *p* < 0.01

r(n) = correlation coefficient (r) with corresponding sample size (n); R^2^ = coefficient of determination; ηₚ^2^ = partial eta squared (effect size measure).

**Table 4:** Distribution of Modified Checklist for Autism in Toddlers, Revised with Follow-Up (M-CHAT-R/F) Risk Classifications According to Screen Exposure Before 12 Months of Age Among Children Aged 16–30 Months in Georgia, April 2025–January 2026.

		Exposure before 12 months of age		Total
		No	Yes	
M-CHAT-R/F Categories	Low Risk	776	621	1397
	Medium Risk	102	220	322
	High Risk	30	59	89
Total		908	900	1808

In the full model containing all the variables, among the daily screen time variables, only average daily current screen time was statistically significant in both the pairwise deletion and multiple imputation analyses. Exposure before 12 months of age was also consistently statistically significant in both models. No variable that was significant under pairwise deletion lost significance after multiple imputation. In contrast, several variables became statistically significant after imputation, average daily screen time during the past 6 months (*P* < 0.01 vs *P* = 0.05), average monthly household income (P < 0.01 vs *P* = 0.02), ethnicity (P < 0.01 vs *P* = 0.16), and place of residence (P < 0.01 vs *P* = 0.34). Average daily screen time during the past year, paternal education, and religious belonging of the family remained non-significant in both models (Table 5).

**Table 5:** Multivariable Linear Regression Results for Factors Associated With Modified Checklist for Autism in Toddlers, Revised With Follow-Up (M-CHAT-R/F) Scores Among Children Aged 16–30 Months in Georgia, April 2025–January 2026.

Pairwise deletion analysis									
Model		Unstandardized Coefficients		Standardized Coefficients	t	p	95.0% Confidence Interval for B		Collinearity Statistics
		B	Std. Error	Beta	t	p	Lower Bound	Upper Bound	VIF
1	(Constant)	−1.61	0.59		−2.75	< 0.01	−2.76	−0.46	
	Age in months	0.12	0.02	0.18	7.16	< 0.01	0.08	0.15	1.06
	Biologic Sex	0.47	0.14	0.08	3.35	< 0.01	0.19	0.74	1.00
	Exposure before 12 months of age	0.45	0.14	0.08	3.07	< 0.01	0.16	0.73	1.14
	Average Daily Screen Time Duration (Recent use)	0.01	0.00	0.19	5.89	< 0.01	0.01	0.01	1.71
	Average Daily Screen Time Duration (For The Past 6 months)	0.00	0.00	0.09	1.99	0.05	0.00	0.01	3.69
	Average Daily Screen Time Duration (For the past year)	0.000	0.00	0.01	0.34	0.74	−0.00	0.00	3.02
	Average Monthly Household Income (Georgian Lari)	−4.18E-5	0.00	−0.06	−2.38	0.02	0.00	0.00	1.06
	Level of Education (Mother)	−0.40	0.10	−0.11	−3.89	< 0.01	−0.600	−0.20	1.41
	Level of Education (Father)	−0.09	0.09	−0.03	−0.95	0.34	−0.27	0.10	1.40
	Religious Belonging of the Family	−0.05	0.09	−0.01	−0.57	0.57	−0.24	0.13	1.06
	Ethnicity	0.10	0.07	0.04	1.42	0.16	−0.04	0.24	1.11
	Place of Residence	0.07	0.08	0.02	0.95	0.35	−0.08	0.22	1.06


Pairwise deletion model summary									
Model	R	R Square	Adjusted R Square	Std. Error of the Estimate	Change Statistics				
					R Square Change	F Change	df1	df2	p
1	0.43	0.19	0.18	2.55	0.19	26.91	12	1403	< 0.01


Multiple imputation analysis									
Model		Unstandardized Coefficients		Standardized Coefficients	t	p	95.0% Confidence Interval for B		Collinearity Statistics
		B	Std. Error	Beta	t	p	Lower Bound	Upper Bound	VIF
1	(Constant)	−1.101	0.211		−5.21	< 0.01	−1.52	−0.69	
	Age in months	0.09	0.01	0.14	15.21	< 0.01	0.08	0.10	1.07
	Biologic Sex	0.50	0.05	0.09	9.82	< 0.01	0.40	0.60	1.00
	Exposure before 12 months of age	0.44	0.05	0.08	8.34	< 0.01	0.34	0.54	1.13
	Average Daily Screen Time Duration (Recent use)	0.01	0.00	0.18	15.42	< 0.01	0.01	0.01	1.72
	Average Daily Screen Time Duration (For The Past 6 months)	0.00	0.00	0.09	5.34	< 0.01	0.00	0.00	3.89
	Average Daily Screen Time Duration (For the past year)	0.00	0.00	0.02	1.36	0.18	0.00	0.00	3.15
	Average Monthly Household Income (Georgian Lari)	−3.74E-5	0.00	−0.05	−5.70	< 0.01	0.00	0.00	1.06
	Level of Education (Mother)	−0.42	0.04	−0.12	−11.15	< 0.01	−0.49	−0.34	1.41
	Level of Education (Father)	−0.07	0.03	−0.02	−1.91	0.06	−0.13	0.00	1.39
	Religious Belonging of the Family	0.02	0.03	0.00	0.06	0.95	−0.06	0.06	1.07
	Ethnicity	0.10	0.03	0.04	3.84	< 0.01	0.05	0.15	1.10
	Place of Residence	0.09	0.03	0.03	2.91	< 0.01	0.03	0.13	1.05


Multiple imputation model summary									
Model	R	R Square	Adjusted R Square	Std. Error of the Estimate	Change Statistics				
					R Square Change	F Change	df1	df2	p
1	0.42	0.17	0.17	2.52	0.17	181.14	12	10,408	< 0.01

General linear models were constructed with M-CHAT-R/F score treated as a continuous outcome. In the first model, only maternal education level and age at onset of screen exposure remained significant predictors (*p* < 0.01), while biologic sex and paternal education were not. A second model replacing education variables with religious affiliation also remained statistically significant; however, only age at onset of screen exposure retained significance (*p* = 0.01) (Table 5).

A third model incorporating current average daily screen time together with parental education and exposure timing significantly increased explained variance (ηp^2^ = 0.44, p < 0.01), with both current screen time duration and age at onset of exposure remaining significant predictors (p < 0.01) (Table 5).

In the final model, religious affiliation, ethnicity, and place of residence were combined; however, the overall model was not statistically significant (*p* = 0.13).

## 4 Discussion

In this large cross-sectional sample of toddlers aged 16–30 months, we observed consistent associations between screen time exposure measured in minutes per day and M-CHAT-R/F screening outcomes. Higher average daily screen time at the time of assessment, as well as greater exposure over the preceding six and twelve months, was associated with higher M-CHAT-R/F scores, although effect sizes were small. At the same time, earlier initiation of screen exposure, particularly before 12 months of age, was associated with a higher likelihood of moderate- and high-risk screening classifications. These findings suggest that both the quantity and timing of early screen exposure may influence autism screening results during a developmentally sensitive period.

Multivariate and general linear models demonstrated that these associations persisted after adjustment for key sociodemographic factors, including household income and parental education, indicating that screen exposure was independently associated with variation in screening scores. The inverse associations observed with maternal education and household income are consistent with a broader literature highlighting the protective role of socioeconomic resources, potentially through increased parental mediation, enriched language environments, or reduced reliance on passive screen use. At the same time, the absence of significant associations for ethnicity, religious affiliation, or place of residence within this largely homogeneous sample suggests that the main findings were not explained by the measured cultural or geographic subgrouping in this dataset.

The first year of life represents a highly sensitive neurodevelopmental period characterized by rapid synaptogenesis, pruning, and heightened experience-dependent plasticity (Law et al., 2023; Priscilla et al., 2025). During this time, environmental inputs may exert lasting effects on neural circuits and later cognitive, motor, and social development (Law et al., 2023; Priscilla et al., 2025).

Accumulating evidence suggests that the timing of screen exposure may be more consequential than cumulative duration alone. Early infancy screen exposure has been associated with developmental delay, motor impairment, and alterations in EEG markers related to attention and executive function (Priscilla et al., 2025; Supanitayanon et al., 2020; Nelson 3rd and Gabard-Durnam, 2020). Children exposed to screens before 12 months also demonstrate higher risks of delayed gross and fine motor development, even after adjustment for sociodemographic confounders (Supanitayanon et al., 2020; Nelson 3rd and Gabard-Durnam, 2020). Neurophysiological studies further suggest that early exposure may influence later executive functioning through altered neural development (Priscilla et al., 2025). Together, these findings support the importance of exposure timing during sensitive developmental periods.

Evidence also supports an independent role of age at screen introduction. One longitudinal cohort study found that introducing screens after approximately 2 years of age was associated with better cognitive performance at 4 years, irrespective of total screen time (Inguaggiato et al., 2017). Because infancy represents a particularly sensitive neurodevelopmental period (Law et al., 2023), early screen exposure—especially when it replaces caregiver interaction—may exert stronger effects on developing neural networks than exposure initiated later in childhood.

Our results also align with current guidance from the American Academy of Pediatrics and the World Health Organization, which recommend avoiding or minimizing screen exposure in infancy while prioritizing direct social interaction (White et al., 2013; World Health Organization, 2019).

Dose–response relationships have also been reported. Greater screen exposure has been associated with worse social communication outcomes and higher ASD-like symptoms in both cross-sectional and longitudinal studies (Alrahili et al., 2021; Heffler et al., 2020; Takahashi et al., 2023). Similar associations have additionally been observed in large Japanese birth cohorts (Takahashi et al., 2023).

Systematic reviews and meta-analyses further support associative links between greater screen exposure and ASD-related outcomes, although causality cannot be inferred and effect sizes remain modest (Ophir et al., 2023a; Sarfraz et al., 2023b; Ophir et al., 2023b). Within ASD populations, higher screen exposure has also been associated with greater symptom severity and lower developmental quotients (Liu and Zhu, 2024; Wang et al., 2024), findings that may still carry population-level significance (Bölte et al., 2023).

Parental and sociodemographic factors also appear to influence early screen exposure. Lower socioeconomic status has been associated with greater screen exposure risk (Cillero and Jago, 2010; Atkin et al., 2014), while parental education and cultural factors may affect caregiver attitudes toward screen use and adherence to medical guidance (Pons et al., 2020; Certain and Kahn, 2002; Kourlaba et al., 2009; Cillero and Jago, 2010; Atkin et al., 2014; Auxier et al., 2020; Evans et al., 2011; Barber et al., 2017; Lowe et al., 2023). Ethnic differences have also been reported, although findings remain inconsistent across populations (Barber et al., 2017; Lowe et al., 2023).

Although the directionality between screen exposure and developmental characteristics is sometimes debated, evidence suggests that screen use is largely parent-driven. Studies from large cohorts demonstrate that parental behaviors, attitudes, and family context are stronger predictors of early screen exposure than child temperament (Chakhunashvili and Chakhunashvili, 2025; Barber et al., 2017; Howe et al., 2017). Nevertheless, bidirectional influences cannot be entirely excluded (Chakhunashvili and Chakhunashvili, 2025).

Several limitations warrant consideration. The cross-sectional design precludes causal inference, and early developmental differences may have influenced caregiver screen use. Screen exposure was parent-reported and subject to recall and social desirability bias. Autism risk was assessed using the M-CHAT-R/F, a screening rather than diagnostic instrument, which may capture broader developmental vulnerabilities. Although sensitivity analyses supported robustness, residual confounding—particularly related to caregiving quality and parental mental health—cannot be excluded. Finally, the relatively homogeneous study population may limit generalizability to more diverse cultural contexts.

These findings reinforce existing recommendations emphasizing limited screen exposure during infancy and highlight the importance of early caregiver counseling regarding screen use. They also support the role of pediatricians in providing evidence-informed guidance, particularly for families with fewer socioeconomic resources.

Future studies should prioritize longitudinal designs and objective measures of screen exposure to better clarify temporal relationships and reduce reporting bias. Further research should additionally examine differences in screen content and modes of engagement, including passive versus interactive and caregiver-mediated versus solitary exposure.

## 5 Conclusions

We can conclude, that both the amount and timing of screen exposure were associated with autism like behavior. Higher average daily screen time, quantified in minutes per day, and earlier initiation of screen exposure (particularly before 12 months of age) were linked to higher screening scores and an increased likelihood of moderate- and high-risk classifications. These associations persisted after adjustment for key sociodemographic factors, including parental education and household income, suggesting that screen exposure was independently associated with screening results. These findings should be interpreted within the context of a screening framework and do not imply a causal relationship between screen time and autism spectrum disorder. These findings support cautious interpretation of early screen exposure in relation to autism screening outcomes and reinforce existing guidance emphasizing interactive caregiving during infancy, while limit the exposure to screens. Future longitudinal studies incorporating objective measures of screen use and diagnostic follow-up are needed to clarify causal pathways and long-term developmental implications.

The following are the supplementary data related to this article.

**Supplementary fig S1:**
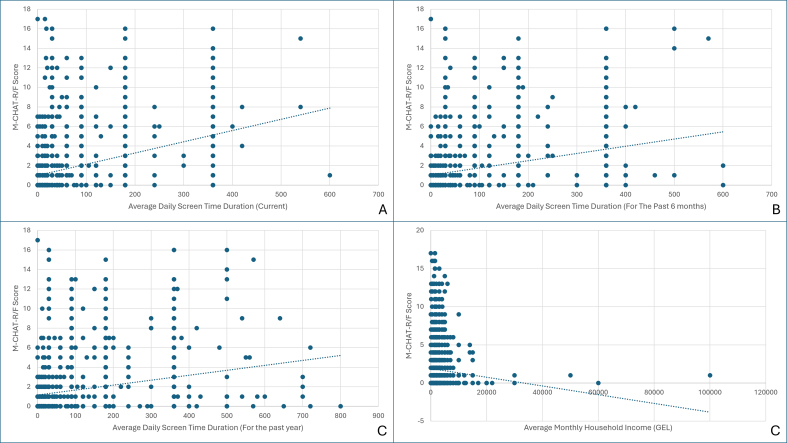
Linear Relationships Between Modified Checklist for Autism in Toddlers, Revised With Follow-Up (M-CHAT-R/F) Scores and Average Daily Screen Time at Assessment (A), During the Past 6 Months (B), During the Past 12 Months (C), and Average Monthly Household Income (D) Among Children Aged 16–30 Months in Georgia, April 2025–January 2026.

**Supplementary fig S2:**
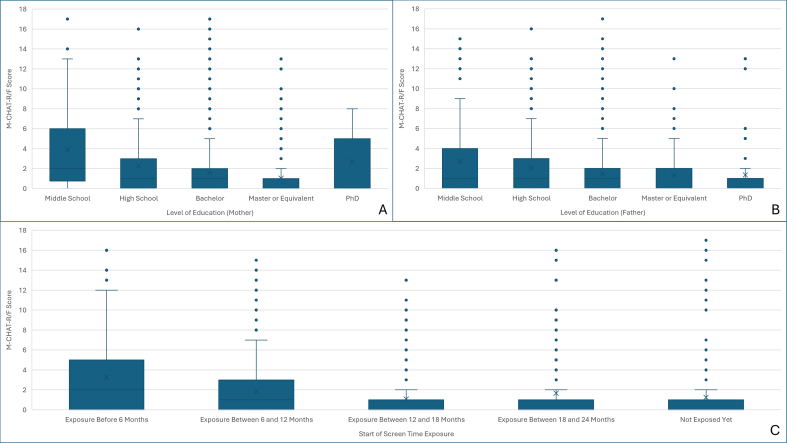
Relationships Between Modified Checklist for Autism in Toddlers, Revised With Follow-Up (M-CHAT-R/F) Scores and Maternal Education (A), Paternal Education (B), and Timing of Screen Exposure Initiation (C) Among Children Aged 16–30 Months in Georgia, April 2025–January 2026.

Supplementary material 1 Sociodemographic Characteristics According to Timing of Screen Exposure (Before 12 Months, After 12 Months, and Not Exposed Yet) Among Children Aged 16–30 Months in Georgia, April 2025–January 2026. Categorical variables are presented as n (%), and continuous variables as mean (SD). Percentages represent column percentages based on non-missing values for each variable.

Supplementary material 2 General Linear Model Results for Factors Associated With Modified Checklist for Autism in Toddlers, Revised With Follow-Up (M-CHAT-R/F) Scores Among Children Aged 16–30 Months in Georgia, April 2025–January 2026.

## Clinical trial number

Clinical Trial Number:Not Applicable.

## CRediT authorship contribution statement

**Konstantine Chakhunashvili:** Writing – review & editing, Writing – original draft, Supervision, Methodology, Investigation, Formal analysis, Data curation, Conceptualization. **Davit G. Chakhunashvili:** Writing – review & editing, Writing – original draft, Project administration, Methodology, Investigation, Formal analysis, Data curation, Conceptualization. **Chidhambara Krishna Muthuvelayutham Sangaranachiar:** Writing – review & editing, Writing – original draft, Methodology, Formal analysis. **Shri Subaa Mathy Muthuvelayutham Sangaranachiar:** Writing – review & editing, Writing – original draft, Validation, Formal analysis.

## Consent for publication

Not Applicable

## Ethics approval and consent to participate

The study was conducted in accordance with the principles of the Declaration of Helsinki and was approved by the Ethical Committee of The University of Georgia (Study code: UGREC-32-23). Informed consent was obtained from each parent and/or legal guardian.

## Funding

N/A

## Declaration of competing interest

The authors declare that they have no known competing financial interests or personal relationships that could have appeared to influence the work reported in this paper.

## Data Availability

The generated data is in supplemental file 1

## References

[bb0005] Alrahili N.M., Almarshad N., Alturki A., Alenazi S., Aljrboa A., Alageel A.A. (2021). The association between screen time exposure and autism spectrum disorder-like symptoms in children. Cureus.

[bb0010] Atkin A.J., Sharp S.J., Corder K., van Sluijs E.M., International children’s Accelerometry database (ICAD) collaborators (2014). Prevalence and correlates of screen time in youth: an international perspective. Am. J. Prev. Med..

[bb0015] Auxier B., Anderson M., Perrin A., Turner E. (2020).

[bb0020] Barber S.E., Kelly B., Collings P.J., Nagy L., Bywater T., Wright J. (2017). Prevalence, trajectories, and determinants of television viewing time in an ethnically diverse sample of young children from the UK. Int. J. Behav. Nutr. Phys. Act..

[bb0025] Bölte S., Leifler E., Berggren S., Borg J. (2023). Patterns and impact of technology use in autistic children. Res. Autism Spectr. Disord..

[bb0030] Certain L.K., Kahn R.S. (2002). Prevalence, correlates, and trajectory of television viewing among infants and toddlers. Pediatrics.

[bb0035] Chakhunashvili K., Chakhunashvili D.G. (2025). Does early screentime exposure or duration affect M-CHAT-R autism screening tool score?. BMC Pediatr..

[bb0040] Chakhunashvili K., Kvirkvelia E., Chakhunashvili D.G. (2024). Does screen time do more damage in boys than girls?. Cureus.

[bb0045] Cillero I.H., Jago R. (2010). Systematic review of correlates of screen-viewing among young children. Prev. Med..

[bb0050] Council on Communications and Media (2016). Media and young minds. Pediatrics.

[bb0055] Evans C.A., Jordan A.B., Horner J. (2011). Only two hours? A qualitative study of the challenges parents perceive in restricting child television time. J. Fam. Issues.

[bb0060] Heffler K.F., Sienko D.M., Subedi K., McCann K.A., Bennett D.S. (2020). Association of Early-Life Social and Digital Media Experiences with Development of autism Spectrum disorder-like symptoms. JAMA Pediatr..

[bb0065] Howe A.S., Heath A.M., Lawrence J., Galland B.C., Gray A.R., Taylor B.J., Sayers R., Taylor R.W. (2017). Parenting style and family type, but not child temperament, are associated with television viewing time in children at two years of age. PLoS One.

[bb0070] Inguaggiato E., Sgobio C., Buffo A. (2017). Brain plasticity and early development: implications for sensitive periods. Dev. Psychobiol..

[bb0075] Kourlaba G., Kondaki K., Liarigkovinos T., Manios Y. (2009). Factors associated with television viewing time in toddlers and preschoolers in Greece: the GENESIS study. J. Public Health (Oxf.).

[bb0080] Law E.C., Han J., Wilkinson L. (2023). Associations between infant screen use, electroencephalography markers, and cognitive outcomes. JAMA Pediatr..

[bb0085] Liu H., Zhu X. (2024). The association between screen exposure and autism spectrum disorder in children: meta-analysis. Rev. Environ. Health.

[bb0090] Lowe B.M., Smith M., Jaine R., Stanley J., Gage R., Signal L. (2023). Watching the watchers: assessing the nature and extent of children’s screen time using wearable cameras. N. Z. Med. J..

[bb0095] Nelson C.A., Gabard-Durnam L.J. (2020). Early adversity and critical periods: neurodevelopmental framework. Trends Neurosci..

[bb0100] Ophir Y., Rosenberg H., Tikochinski R., Dalyot S., Lipshits-Braziler Y. (2023). Screen time and autism spectrum disorder: a systematic review and Meta-analysis. JAMA Netw. Open.

[bb0105] Ophir Y., Tikochinski R., Asterhan C.S.C., Sisso I., Reichart R. (2023). Screen time and autism spectrum disorder: a systematic review and meta-analysis. JAMA Netw. Open.

[bb0110] Pons M., Bennasar-Veny M., Yañez A.M. (2020). Maternal education level and excessive recreational screen time in children: a mediation analysis. Int. J. Environ. Res. Public Health.

[bb0115] Priscilla A.P. (2025). Screen exposure and early childhood development in resource-limited regions: findings from a population-based survey study. J. Med. Internet Res..

[bb0120] Robins D.L., Casagrande K., Barton M., Chen C.M., Dumont-Mathieu T., Fein D. (2014). Validation of the modified checklist for autism in toddlers, revised with follow-up (M-CHAT-R/F). Pediatrics.

[bb0125] Sarfraz S., Shlaghya G., Narayana S.H., Mushtaq U., Shaman Ameen B., Nie C., Nechi D., Mazhar I.J., Yasir M., Arcia Franchini A.P. (2023). Early screen-time exposure and its association with risk of developing autism spectrum disorder: a systematic review. Cureus.

[bb0130] Sarfraz S., Shlaghya G., Narayana S.H., Mushtaq U., Ameen B.S., Nie C., Nechi D., Mazhar I.J., Yasir M., Franchini A.P.A. (2023). Early screen-time exposure and its association with risk of developing autism spectrum disorder: a systematic review. Cureus.

[bb0135] Supanitayanon S., Trairatvorakul P., Chonchaiya W. (2020). Screen media exposure in the first 2 years of life and preschool cognitive development: a longitudinal study. Pediatr. Res..

[bb0140] Takahashi I., Obara T., Ishikuro M., Murakami K., Ueno F., Noda A., Onuma T., Shinoda G., Nishimura T., Tsuchiya K.J., Kuriyama S. (2023). Screen time at age 1 year and communication and problem-solving developmental delay at 2 and 4 years. JAMA Pediatr..

[bb0145] Wang H., Peng X., Xue Y., Dong H., Ma C., Jia F., Du L. (2024). A study of the effects of screen exposure on neuropsychological development in children with ASD using the ScreenQ. BMC Pediatr..

[bb0150] White E.J., Hutka S.A., Williams L.J., Moreno S. (2013). Learning, neural plasticity and sensitive periods: implications for language acquisition, music training and transfer across the lifespan. Front. Syst. Neurosci..

[bb0155] World Health Organization (2019). https://www.who.int/publications/i/item/9789241550536.

