# Supplementary material for: Screen time and autism like behavior: Cross-sectional study from Georgia
Source: Prev Med Rep. 2026 May 22;67:103503. doi: 10.1016/j.pmedr.2026.103503 (PMC13223515; doi:10.1016/j.pmedr.2026.103503)
Supplement: Supplementary material 1 — Sociodemographic Characteristics According to Timing of Screen Exposure (Before 12 Months, After 12 Months, and Not Exposed Yet) Among Children Aged 16–30 Months in Georgia, April 2025–January 2026. Categorical variables are presented as n (%), and continuous variables as mean (SD). Percentages represent column percentages based on non-missing values for each variable. [file mmc3.pdf]

MCHATRFS	MCHATRFS	Age	Sex	EXP BFR 12	Start of Scr	Average Da	Average Da	AverageDa	Average M	Level of Ed	Level of Ed	Religious B	Ethnicity	Place of Residence
Medium Ri	3	17	Male	No	Not Expose	30	15	40	1000	Bachelor	Bachelor	Orthodox	Georgian	Village
High Risk	12	30	Male	Yes	Exposure B	360	360	369	1500	High Schoo	Middle Sch	Orthodox	Georgian	City/Town
Low Risk	0	29	Male	No	Exposure B	30	30	30	1500	Middle Sch	Middle Sch	Orthodox	Georgian	City/Town
High Risk	17	30	Male	No	Not Expose	0	0	0	0	Bachelor	Bachelor	Orthodox	Georgian	City/Town
Low Risk	2	24	Male	No	Exposure B	40	30	30	2000	High Schoo	High Schoo	Orthodox	Georgian	City/Town
Medium Ri	5	30	Male	Yes	Exposure B	90	90	90	500	Bachelor	High Schoo	Orthodox	Georgian	Village
Low Risk	0	21	Female	Yes	Exposure B	30	40	60	1500	Middle Sch	High Schoo	Orthodox	Georgian	City/Town
Low Risk	0	18	Female	No	Exposure B	30	30	30	5000	Bachelor	Bachelor	Orthodox	Georgian	Village
Low Risk	1	17	Male	No	Not Expose	0	0	0	4000	Master or	Bachelor	Orthodox	Georgian	City/Town
Low Risk	0	21	Male	Yes	Exposure B	1	4	4	9500	Master or	Master or	Orthodox	Georgian	City/Town
Low Risk	0	23	Male	No	Exposure B	30	30	30	6000	Bachelor	Bachelor	Orthodox	Georgian	City/Town
Medium Ri	3	23	Female	Yes	Exposure B	180	360	360	1500	High Schoo	High Schoo	Orthodox	Georgian	Village
Low Risk	0	21	Male	Yes	Exposure B	30	180	360	3000	Master or	Master or	Orthodox	Georgian	City/Town
Low Risk	0	21	Male	Yes	Exposure B	30	180	360	3000	Master or	Master or	Orthodox	Georgian	City/Town
Low Risk	2	18	Male	Yes	Exposure B	60	60	50	1500	Master or	Master or	Orthodox	Georgian	City/Town
Low Risk	1	23	Male	No	Exposure B	30	30	30	3000	Master or	Master or	Orthodox	Georgian	City/Town
Low Risk	1	24	Male	Yes	Exposure B	180	500	500	5000	Master or	Bachelor	Orthodox	Georgian	City/Town
Low Risk	2	17	Male	No	Exposure B	30	180	180	3000	Bachelor	Bachelor	Orthodox	Georgian	City/Town
Low Risk	2	27	Female	No	Exposure B	90	360	360	3000	Bachelor	High Schoo	Orthodox	Georgian	Village
Low Risk	0	23	Female	Yes	Exposure B	90	30	90	1500	Bachelor	Master or	Orthodox	Georgian	City/Town
Low Risk	0	27	Male	No	Not Expose	0	0	0	1500	Master or	Master or	Orthodox	Georgian	City/Town
Medium Ri	3	26	Male	No	Exposure B	0	30	50	1200	High Schoo	High Schoo	Orthodox	Georgian	City/Town
Low Risk	0	17	Male	No	Not Expose	0	0	0	4000	Master or	Bachelor	Orthodox	Georgian	Village
Low Risk	1	18	Male	Yes	Exposure B	30	360	360	4000	Master or	High Schoo	Orthodox	Georgian	Village
Medium Ri	3	26	Male	No	Exposure B	10	120	90	1200	High Schoo	High Schoo	Orthodox	Georgian	City/Town
Low Risk	1	21	Female	No	Exposure B	30	180	30	1500	Middle Sch	High Schoo	Orthodox	Georgian	City/Town
Low Risk	1	20	Male	Yes	Exposure B	10	30	90	4000	Bachelor	Bachelor	Orthodox	Georgian	City/Town
High Risk	16	30	Male	No	Exposure B	18	360	360	1500	High Schoo	High Schoo	Orthodox	Georgian	City/Town
Low Risk	1	27	Female	No	Exposure B	20	0	30	1000	High Schoo	High Schoo	Orthodox	Georgian	Village
Medium Ri	4	18	Male	Yes	Exposure B	0	360	360	1800	Bachelor	High Schoo	Orthodox	Georgian	City/Town
Low Risk	0	24	Female	Yes	Exposure B	40	40	40	3000	High Schoo	High Schoo	Orthodox	Georgian	City/Town
Low Risk	2	18	Male	No	Exposure B	20	90	180	500	High Schoo	High Schoo	Orthodox	Georgian	City/Town
Low Risk	1	23	Female	Yes	Exposure B	180	180	90	3000	High Schoo	Master or	Orthodox	Georgian	City/Town
Low Risk	0	23	Female	Yes	Exposure B	180	180	180	3000	Bachelor	Bachelor	Orthodox	Georgian	City/Town
High Risk	8	27	Male	Yes	Exposure B	90	360	360	1500	Bachelor	High Schoo	Orthodox	Georgian	City/Town
Low Risk	0	17	Female	No	Exposure B	20	30	90	3000	Bachelor	Master or	Orthodox	Georgian	City/Town
Low Risk	0	19	Female	Yes	Exposure B	90	30	60	3500	Bachelor	Master or	Orthodox	Georgian	City/Town
Low Risk	1	29	Male	No	Exposure B	30	30	90	3000	Master or	Master or	Orthodox	Georgian	Village
Medium Ri	6	24	Female	Yes	Exposure B	90	180	200	3000	High Schoo	Middle Sch	Orthodox	Armenian	City/Town
Low Risk	0	20	Male	No	Not Expose	0	0	0	5000	Bachelor	High Schoo	Orthodox	Georgian	Village
Low Risk	0	26	Male	No	Not Expose	0	30	30	3000	Bachelor	Bachelor	Other	Georgian	City/Town
Low Risk	1	24	Male	No	Exposure B	10	20	20	600	High Schoo	High Schoo	Orthodox	Georgian	City/Town
Medium Ri	3	30	Male	Yes	Exposure B	30	30	30	600	High Schoo	High Schoo	Orthodox	Georgian	City/Town

Low Risk	0	24	Male	Yes	Exposure B	30	30	30	10000	Master or I	Master or I	Orthodox	Georgian	Emigrant (West)
Medium Ri	7	18	Male	Yes	Exposure B	360	360	360	3000	Bachelor	Master or I	Orthodox	Georgian	Village
Low Risk	0	20	Male	No	Not Expose	0	0	0	0	Bachelor	Bachelor	Orthodox	Georgian	City/Town
Low Risk	0	29	Female	No	Exposure B	90	180	360	800	High Schoo	High Schoo	Orthodox	Georgian	City/Town
Low Risk														
Medium Ri	7	30	Male	No	Exposure B	0	30	30	3000	Master or I	Master or I	Orthodox	Georgian	City/Town
Medium Ri	3	30	Male	No	Not Expose	0	0	0	3000	Master or I	Master or I	Orthodox	Georgian	Village
Low Risk	1	29	Male	Yes	Exposure B	30	30	30	2000	Middle Sch	High Schoo	Orthodox	Georgian	City/Town
Medium Ri	4	30	Male	No	Exposure B	90	180	180	1500	High Schoo	Middle Sch	Orthodox	Georgian	City/Town
Medium Ri	4	24	Male	Yes	Exposure B	90	90	90	15000	Master or I	Master or I	Orthodox	Georgian	City/Town
Low Risk	0	27	Female	Yes	Exposure B	30	30	30	5000	Bachelor	High Schoo	Orthodox	Georgian	City/Town
Low Risk	0	18	Female	No	Exposure B	90	90	30	3000	Master or I	Bachelor	Orthodox	Georgian	City/Town
Low Risk	0	19	Female	Yes	Exposure B	90	180	180	4000	Bachelor	Bachelor	Orthodox	Georgian	City/Town
Low Risk	0	25	Male	No	Exposure B	15	15	15	4000	Master or I	Bachelor	Orthodox	Georgian	City/Town
Low Risk	0	20	Female	Yes	Exposure B	40	40	40	5000	Master or I	Master or I	Orthodox	Georgian	City/Town
Medium Ri	3	30	Female	No	Not Expose	0	0	0		High Schoo	Master or I	Orthodox	Georgian	Village
Low Risk	0	28	Female	No	Exposure B	30	60	60	2500	Bachelor	Bachelor	Orthodox	Georgian	Village
Low Risk	0	27	Male	Yes	Exposure B	30	30	30	60000	High Schoo	High Schoo	Orthodox	Georgian	City/Town
Low Risk	1	28	Female	No	Exposure B	40	30	20	3000	Master or I	PhD	Orthodox	Georgian	City/Town
Low Risk	0	18	Female	Yes	Exposure B	30	90	180	3000	Bachelor	Middle Sch	Orthodox	Georgian	City/Town
Low Risk	0	25	Female	Yes	Exposure B	30	30	30	3000	Master or I	Bachelor	Orthodox	Georgian	City/Town
Low Risk	0	23	Male	No	Exposure B	30	30	30	500	Bachelor	Bachelor	Orthodox	Georgian	Emigrant (West)
Low Risk	2	24	Female	No	Not Expose	90	90	90	1500	Master or I	Bachelor	Orthodox	Georgian	City/Town
Low Risk	0	18	Female	Yes	Exposure B	15	15	15	10000	PhD	Bachelor	Atheist	Mixed	Emigrant (West)
Low Risk	1	18	Male	No	Not Expose	1	1	1	1600	High Schoo	Bachelor	Orthodox	Georgian	City/Town
Low Risk	0	26	Male	Yes	Exposure B	30	60	60	10000	Master or I	Master or I	Orthodox	Georgian	City/Town
Low Risk	1	24	Male	Yes	Exposure B	90	90	90	3500	Bachelor	High Schoo	Orthodox	Georgian	City/Town
Low Risk	1	24	Male	No	Exposure B	30	30	15	7000	Master or I	Master or I	Orthodox	Georgian	City/Town
Low Risk	1	24	Male	Yes	Exposure B	90	90	90	3000	Bachelor	High Schoo	Orthodox	Georgian	City/Town
Low Risk	1	18	Male	Yes	Exposure B	90	90	180	5000	Master or I	Master or I	Orthodox	Georgian	City/Town
Medium Ri	3	18	Male	Yes	Exposure B	360	360	360	5000	Bachelor	High Schoo	Orthodox	Georgian	Village
Low Risk	0	21	Male	Yes	Exposure B	90	90	30	2000	Master or I	Bachelor	Orthodox	Georgian	City/Town
Medium Ri	6	22	Male	Yes	Exposure B	30	180	180	180	Bachelor	Bachelor	Orthodox	Georgian	City/Town
Low Risk	0	25	Female	Yes	Exposure B	90	90	90	3000	Bachelor	Bachelor	Orthodox	Georgian	Village
Low Risk	0	26	Female	No	Exposure B	30	60	20	1500	Master or I	Bachelor	Orthodox	Georgian	City/Town
Low Risk	1	18	Female	No	Not Expose	10	60	90	5000	Master or I	Master or I	Orthodox	Georgian	City/Town
Low Risk	0	17	Female	No	Exposure B	15	15	10		Master or I	Master or I	Orthodox	Georgian	City/Town
Low Risk	0	21	Female	No	Exposure B	90	90	90	2000	Master or I	Master or I	Orthodox	Georgian	City/Town
Low Risk	0	21	Female	No	Not Expose	0	0	0	7000	Master or I	Bachelor	Orthodox	Georgian	City/Town
Low Risk	0	20	Male	No	Exposure B	30	60	60	12000	Bachelor	Bachelor	Orthodox	Georgian	Emigrant (West)
Low Risk	1	29	Male	No	Exposure B	90	360	700	1500	High Schoo	High Schoo	Orthodox	Georgian	City/Town
Low Risk	0	27	Male	Yes	Exposure B	30	90	90	1500	Bachelor	Bachelor	Orthodox	Georgian	City/Town
Low Risk	0	29	Male	No	Exposure B	180			1500	High Schoo	High Schoo	Orthodox	Georgian	City/Town
Medium Ri	4	19	Female	Yes	Exposure B	60	60	15	14000	Master or I	Master or I	Orthodox	Georgian	City/Town

Low Risk	1	21	Male	Yes	Exposure B	180	180	180	0	Master or I	Master or I	Orthodox	Georgian	City/Town
Low Risk	0	28	Male	Yes	Exposure B	90	90	90	1500	Master or I	Bachelor	Orthodox	Georgian	City/Town
Low Risk	0	20	Male	No	Not Expose	0	0	0	2500	Master or I	Master or I	Orthodox	Georgian	City/Town
Low Risk	0	17	Male	No	Not Expose	0	0	0	2500	Bachelor	Master or I	Orthodox	Georgian	City/Town
Low Risk	0	29	Female	No	Not Expose	0	5	10	2000	Master or I	Master or I	Orthodox	Georgian	Emigrant (West)
Low Risk	0	22	Female	No	Exposure B	30	30	30	5000	Master or I	Bachelor	Orthodox	Georgian	City/Town
Low Risk	0	24	Male	Yes	Exposure B	90	90	120	5000	Master or I	PhD	Orthodox	Georgian	City/Town
Low Risk	0	28	Female	No	Exposure B	90	90	90	1500	Bachelor	Bachelor	Orthodox	Georgian	City/Town
Low Risk	0	17	Male	No	Not Expose	0	0	0	12000	Bachelor	Bachelor	Orthodox	Georgian	City/Town
Low Risk	2	21	Male	No	Not Expose	0	0	0	1500	Bachelor	Bachelor	Orthodox	Georgian	Emigrant (West)
Medium Ri	7	20	Female	No	Not Expose	30	30	30	3000	Bachelor	High Schoo	Orthodox	Georgian	City/Town
Medium Ri	3	30	Male	Yes	Exposure B	90	90	90	1500	Bachelor	High Schoo	Orthodox	Georgian	City/Town
Low Risk	1	27	Male	No	Exposure B	50	45	45	3000	Bachelor	High Schoo	Orthodox	Georgian	City/Town
Low Risk	1	29	Female	Yes	Exposure B	45	240	500	12000	Master or I	PhD	Orthodox	Georgian	City/Town
Low Risk	1	25	Male	No	Not Expose	180	180	90	3000	Bachelor	High Schoo	Orthodox	Georgian	City/Town
Low Risk	0	23	Male	No	Exposure B	30	30	30	1500	Bachelor	Bachelor	Orthodox	Georgian	City/Town
Low Risk	0	27	Male	Yes	Exposure B	60	60	30	1500	Bachelor	Bachelor	Orthodox	Georgian	City/Town
Low Risk	0	29	Male	Yes	Exposure B	45	360	720		Bachelor	Master or I	Orthodox	Georgian	City/Town
Low Risk	1	26	Male	Yes	Exposure B	30	30	60	3000	Bachelor	Bachelor	Orthodox	Georgian	City/Town
Low Risk	0	27	Female	No	Exposure B	5	20	30	1500	High Schoo	High Schoo	Orthodox	Georgian	City/Town
High Risk	12	18	Male	Yes	Exposure B	40	40	30	5000	Master or I	PhD	Orthodox	Georgian	City/Town
Low Risk	1	18	Male	No	Exposure B	30	30	30	3000	Bachelor	High Schoo	Orthodox	Georgian	Emigrant (West)
Low Risk	0	23	Female	No	Exposure B	15	25	360	2000	Bachelor	High Schoo	Orthodox	Georgian	City/Town
Low Risk	1	24	Male	No	Exposure B	90	90	40	30000	Master or I	Master or I	Orthodox	Georgian	City/Town
Low Risk	0	18	Female	Yes	Exposure B	90	400	650	2500	Bachelor	High Schoo	Orthodox	Georgian	City/Town
Low Risk	0	25	Female	Yes	Exposure B	0	200	240	3000	Master or I	Master or I	Orthodox	Georgian	City/Town
Low Risk	1	17	Male	No	Exposure B	18	30	30	5000	Master or I	Bachelor	Orthodox	Georgian	City/Town
Low Risk	0	29	Male	Yes	Exposure B	60	180	400	4000	Bachelor	High Schoo	Orthodox	Georgian	City/Town
Low Risk	0	25	Female	No	Exposure B	30	10	5	2000	Bachelor	Bachelor	Orthodox	Georgian	City/Town
Low Risk	0	28	Male	No	Exposure B	30	30	30	3000	Bachelor	Bachelor	Orthodox	Georgian	City/Town
Medium Ri	3	30	Male	Yes	Exposure B	90	180	180	1100	High Schoo	High Schoo	Orthodox	Georgian	City/Town
Low Risk	1	23	Female	No	Exposure B	30	15	5	4000	Bachelor	Bachelor	Orthodox	Georgian	City/Town
Low Risk	0	29	Female	No	Not Expose	0	0	0	5000	Bachelor	Bachelor	Orthodox	Georgian	City/Town
Low Risk	1	25	Male	No	Not Expose	30	15	15	5000	Master or I	Master or I	Orthodox	Georgian	City/Town
Low Risk	0	28	Female	No	Exposure B	45	60	60	9000	PhD	Bachelor	Orthodox	Georgian	Emigrant (West)
Low Risk	1	18	Male	Yes	Exposure B	30	50	0	6000	Bachelor	Master or I	Orthodox	Georgian	City/Town
Low Risk	0	28	Male	Yes	Exposure B	90	90	45	5000	PhD	PhD	Orthodox	Georgian	City/Town
Low Risk	1	20	Female	Yes	Exposure B	40			4000	Bachelor	High Schoo	Orthodox	Mixed	City/Town
Low Risk	0	25	Male	Yes	Exposure B	90	60	60	10000	Bachelor	Bachelor	Orthodox	Georgian	City/Town
Low Risk	0	20	Male	Yes	Exposure B	120	180	180	2500	Bachelor	High Schoo	Protestant	Georgian	City/Town
Low Risk	2	25	Male	Yes	Exposure B	60	60	60	3000	Bachelor	High Schoo	Orthodox	Mixed	City/Town
Low Risk	1	27	Male	Yes	Exposure B	240	240	200	10000	Master or I	Master or I	Orthodox	Georgian	City/Town
Medium Ri	3	24	Male	Yes	Exposure B	30	30	30	3000	Master or I	High Schoo	Orthodox	Georgian	City/Town
Medium Ri	5	30	Male	Yes	Exposure B	180	180	180	3000	High Schoo	High Schoo	Orthodox	Georgian	Village

Low Risk	0	19	Male	No	Exposure B	20	20	10	4000	Bachelor	Bachelor	Orthodox	Georgian	City/Town
Low Risk	0	21	Male	Yes	Exposure B	10	10	10	1500	Master or I	Master or I	Orthodox	Georgian	City/Town
Low Risk	0	21	Female	Yes	Exposure B	30	30	30	3000	Bachelor	Bachelor	Orthodox	Georgian	City/Town
Low Risk	0	18	Male	No	Not Expose	0	0	0	3000	Master or I	Bachelor	Orthodox	Georgian	City/Town
Low Risk	0	24	Male	Yes	Exposure B	120	120	120	7000	Master or I	Master or I	Atheist	Georgian	City/Town
Low Risk	0	17	Female	No	Exposure B	30	30	30	500	High Schoo	High Schoo	Orthodox	Georgian	City/Town
Medium Ri	3	29	Male	No	Exposure B	30	20	10	5000	Master or I	Master or I	Orthodox	Georgian	City/Town
Low Risk	1	18	Male	Yes	Exposure B	90	90	90	150	Bachelor	High Schoo	Orthodox	Georgian	City/Town
Low Risk	0	17	Female	Yes	Exposure B	10	10	5	4000	Master or I	Bachelor	Other	Georgian	City/Town
Low Risk	1	20	Male	No	Not Expose	0	0	0	1000	Bachelor	Bachelor	Orthodox	Georgian	Village
Low Risk	0	24	Male	Yes	Exposure B	30	360	569	6500	Master or I	Master or I	Atheist	Georgian	City/Town
Low Risk	0	21	Female	No	Exposure B	30	13	13	3000	Bachelor	High Schoo	Orthodox	Georgian	City/Town
Low Risk	0	18	Male	No	Not Expose	0	0	0	3000	Master or I	Bachelor	Orthodox	Georgian	City/Town
Low Risk	0	26	Male	Yes	Exposure B	90	90	90	1500	High Schoo	High Schoo	Orthodox	Georgian	City/Town
Low Risk	0	24	Male	Yes	Exposure B	30	90	90	1500	Bachelor	High Schoo	Orthodox	Georgian	Village
Low Risk	2	19	Female	Yes	Exposure B	90	360	360	3000	PhD	PhD	Other	Other	Emigrant (West)
Low Risk	0	24	Male	No	Exposure B	30	13	90		High Schoo	Middle Sch	Orthodox	Georgian	City/Town
Low Risk	1	18	Female	Yes	Exposure B	90	90	540	1500	High Schoo	High Schoo	Orthodox	Georgian	City/Town
High Risk	11	30	Male	Yes	Exposure B	360	360	360	3000	Bachelor	High Schoo	Orthodox	Georgian	City/Town
Low Risk	1	19	Male	Yes	Exposure B	20	30	30	3000	Bachelor	Bachelor	Orthodox	Georgian	City/Town
Low Risk	0	20	Female	Yes	Exposure B	180	360	90	3000	Bachelor	Bachelor	Orthodox	Georgian	City/Town
Low Risk	0	29	Male	No	Exposure B	60	40	30	6000	Bachelor	Bachelor	Orthodox	Georgian	City/Town
Medium Ri	7	19	Male	No	Not Expose	90	180	180	4000	High Schoo	High Schoo	Orthodox	Georgian	City/Town
Low Risk	0	19	Male	Yes	Exposure B	30	30	30	3000	Bachelor	Bachelor	Orthodox	Georgian	City/Town
Low Risk	2	27	Male	Yes	Exposure B	90	30	90	3000	Bachelor	High Schoo	Orthodox	Georgian	City/Town
Medium Ri	3	30	Male	No	Exposure B	40	250	500	5000	Bachelor	Bachelor	Orthodox	Georgian	Emigrant (West)
Low Risk	1	24	Female	Yes	Exposure B	2	90	30	500	High Schoo	High Schoo	Muslim	Georgian	City/Town
Low Risk	0	26	Female	No	Exposure B	5	75	180	1400	Bachelor	High Schoo	Orthodox	Georgian	City/Town
Low Risk	0	28	Female	No	Exposure B	10	10	10				Other	Georgian	City/Town
Low Risk	0	22	Female	Yes	Exposure B	90	180	200	2000	Bachelor	High Schoo	Orthodox	Georgian	City/Town
Medium Ri	6	19	Male	Yes	Exposure B	30	30	30	1000	Bachelor	High Schoo	Orthodox	Georgian	Village
Medium Ri	7	24	Male	Yes	Exposure B	180	180	180	5000	Bachelor	Bachelor	Orthodox	Mixed	City/Town
Low Risk	2	24	Male	Yes	Exposure B	90	120	120	3000	Bachelor	Master or I	Orthodox	Georgian	City/Town
Low Risk	0	21	Male	Yes	Exposure B	30	180	360	5000	Bachelor	High Schoo	Orthodox	Georgian	City/Town
Medium Ri	6	30	Male	Yes	Exposure B	10	30	60	5000	Master or I	High Schoo	Orthodox	Armenian	Village
Low Risk	1	19	Male	Yes	Exposure B	90	460	580	3000	Bachelor	High Schoo	Muslim	Georgian	City/Town
Medium Ri	6	30	Male	No	Exposure B	250	180	150	4500	Bachelor	Bachelor	Orthodox	Georgian	City/Town
Low Risk	2	20	Male	No	Exposure B	35	60	60	2700	Bachelor	High Schoo	Orthodox	Georgian	Mountainous Region
Low Risk	0	20	Female	Yes	Exposure B	30	30	30	3000	Master or I	Master or I	Atheist	Georgian	City/Town
Medium Ri	7	29	Male	Yes	Exposure B	90	90	180	5000	Master or I	Bachelor	Orthodox	Georgian	City/Town
Low Risk	0	23	Male	Yes	Exposure B	30	90	60	3000	Bachelor	Master or I	Orthodox	Georgian	City/Town
Medium Ri	7	23	Male	Yes	Exposure B	30	30	45	800	High Schoo	Middle Sch	Orthodox	Georgian	Village
Low Risk	1	17	Male	No	Not Expose	0	0	0	3000	Bachelor	Bachelor	Orthodox	Georgian	City/Town
High Risk	9	23	Male	Yes	Exposure B	360	90	360	4000	High Schoo	Bachelor	Orthodox	Georgian	City/Town

Low Risk	0	29	Female	Yes	Exposure B	90	360	360	3000	Master or I	Master or I	Orthodox	Georgian	City/Town
Low Risk	2	20	Male	Yes	Exposure B	90	90	30	1500	Bachelor	Bachelor	Orthodox	Georgian	City/Town
Low Risk	1	19	Male	Yes	Exposure B	30	90	90	2000	Bachelor	Bachelor	Orthodox	Georgian	City/Town
Low Risk	0	21	Male	Yes	Exposure B	180	180	90	5000	Bachelor	Bachelor	Orthodox	Georgian	City/Town
Low Risk	1	18	Female	Yes	Exposure B	30	90	30	3000	Bachelor	Bachelor	Orthodox	Georgian	City/Town
Medium Ri	6	30	Female	No	Exposure B	120	360	720	1500	Bachelor	High Schoo	Orthodox	Georgian	Village
Medium Ri	3	25	Male	Yes	Exposure B	90	30	30	2000	Middle Sch	High Schoo	Orthodox	Georgian	City/Town
Low Risk	1	25	Male	No	Not Expose	5	10	10	2000	Bachelor	Bachelor	Orthodox	Georgian	Village
Low Risk	0	23	Male	No	Not Expose	30	90	180	3000	High Schoo	Bachelor	Orthodox	Georgian	City/Town
Low Risk	1	19	Male	No	Exposure B	10	5	5	5000	Bachelor	Bachelor	Orthodox	Georgian	Emigrant (West)
Medium Ri	3	19	Male	Yes	Exposure B	1	1	1		Master or I	Master or I	Orthodox	Georgian	City/Town
High Risk	12	29	Male	Yes	Exposure B	90	360	360	3000	Bachelor	Bachelor	Orthodox	Georgian	City/Town
Medium Ri	5	17	Male	Yes	Exposure B	30	30	30	10000	Master or I	Master or I	Orthodox	Georgian	City/Town
Low Risk	2	29	Male	No	Exposure B	30	180	180	5000	Bachelor	Bachelor	Orthodox	Georgian	City/Town
Low Risk	0	27	Male	No	Exposure B	60	60	0	2000	Master or I	High Schoo	Orthodox	Georgian	Village
Medium Ri	3	30	Male	Yes	Exposure B	180	180	90	1500	Bachelor	High Schoo	Orthodox	Georgian	Village
Low Risk	0	18	Female	No	Exposure B	90	30	30	2000	Bachelor	Bachelor	Orthodox	Georgian	City/Town
Low Risk	2	22	Female	Yes	Exposure B	120	120	120	3000	High Schoo	High Schoo	Orthodox	Georgian	Village
Low Risk	0	18	Male	Yes	Exposure B	30	30	30	10000	Bachelor	High Schoo	Orthodox	Georgian	Village
Medium Ri	5	17	Male	Yes	Exposure B	60	60	30	5000	Bachelor	Bachelor	Orthodox	Georgian	City/Town
High Risk	9	24	Female	Yes	Exposure B	90	180	180	3000	High Schoo	High Schoo	Orthodox	Armenian	City/Town
Low Risk	0	27	Male	Yes	Exposure B	30	90	90	3000	Bachelor	Bachelor	Orthodox	Georgian	City/Town
High Risk	13	30	Female	Yes	Exposure B	180	180	180	3000	Master or I	Master or I	Orthodox	Georgian	City/Town
Low Risk	0	27	Male	Yes	Exposure B	60	360	360	2500	High Schoo	High Schoo	Orthodox	Georgian	Emigrant (West)
Low Risk	0	18	Female	No	Exposure B	10	150	150	4500	Bachelor	Bachelor	Orthodox	Mixed	City/Town
Low Risk	0	24	Male	No	Not Expose	30	30	360	3000	Bachelor	Bachelor	Orthodox	Other	City/Town
Low Risk	0	28	Male	Yes	Exposure B	90	180	360	5000	Master or I	Bachelor	Orthodox	Georgian	City/Town
Low Risk	0	24	Male	No	Exposure B	30	90	90	5000	Master or I	PhD	Orthodox	Georgian	City/Town
High Risk	9	30	Female	No	Exposure B	90	180	90	1500	Middle Sch	High Schoo	Orthodox	Georgian	City/Town
Medium Ri	4	25	Male	Yes	Exposure B	90	90	90	3000	Bachelor	High Schoo	Orthodox	Georgian	City/Town
High Risk	13	30	Male	No	Exposure B	90	360		1200	Middle Sch	Middle Sch	Orthodox	Georgian	Mountainous Region
High Risk	10	28	Male	Yes	Exposure B	180	189	90	2000	Bachelor	Bachelor	Orthodox	Georgian	City/Town
Medium Ri	4	24	Male	Yes	Exposure B	30	30	80		High Schoo	High Schoo	Orthodox	Georgian	City/Town
Medium Ri	4	18	Male	Yes	Exposure B	60	90	180	5000	Bachelor	Bachelor	Orthodox	Georgian	City/Town
Low Risk	1	22	Female	No	Exposure B	30	60	30	3000	Bachelor	High Schoo	Orthodox	Georgian	Emigrant (West)
Medium Ri	3	25	Female	No	Exposure B	30	30	30	1500	High Schoo	High Schoo	Orthodox	Georgian	Mountainous Region
High Risk	15	28	Male	Yes	Exposure B	540	570	570	3000	Bachelor	Middle Sch	Orthodox	Georgian	City/Town
Low Risk	0	19	Female	No	Exposure B	60	200	400	10000	Bachelor	Master or I	Orthodox	Georgian	City/Town
Low Risk	0	18	Female	Yes	Exposure B	60	60	60	2500	Bachelor	Bachelor	Orthodox	Georgian	City/Town
Low Risk	0	24	Male	Yes	Exposure B	20	15	15	3000	Bachelor	Master or I	Orthodox	Georgian	Village
Low Risk	0	22	Male	No	Exposure B	30	20	10		Master or I	PhD	Orthodox	Georgian	Village
Low Risk	1	22	Male	No	Exposure B	10	10	10	4500	Master or I	Bachelor	Orthodox	Georgian	City/Town
Low Risk	0	22	Female	No	Exposure B	30	30	30	8000	Bachelor	High Schoo	Orthodox	Armenian	City/Town
Low Risk	0	17	Male	Yes	Exposure B	30	30	30	1500	High Schoo	High Schoo	Orthodox	Georgian	City/Town

Low Risk	2	20	Male	No	Exposure B	90	30	30	1000	Bachelor	High Schoo	Orthodox	Georgian	City/Town
Medium Ri	5	24	Male	Yes	Exposure B	90	90	90	3000	Bachelor	Bachelor	Orthodox	Georgian	City/Town
Low Risk	0	29	Male	No	Exposure B	30	30	90	1200	High Schoo	High Schoo	Orthodox	Georgian	Village
Low Risk	0	22	Female	Yes	Exposure B	90	60	40	17000	Bachelor	Bachelor	Orthodox	Georgian	City/Town
Low Risk	1	19	Female	No	Exposure B	30	30	30	1500	Bachelor	Bachelor	Orthodox	Georgian	City/Town
Low Risk	0	19	Female	No	Not Expose	15	15	15	1500	High Schoo	High Schoo	Orthodox	Georgian	City/Town
Low Risk	1	19	Female	No	Not Expose	0	15	15	1500	High Schoo	High Schoo	Orthodox	Georgian	City/Town
Medium Ri	3	30	Female	Yes	Exposure B	90	90	90	1500	Middle Sch	High Schoo	Orthodox	Georgian	Mountainous Region
Low Risk	0	21	Male	No	Exposure B	20	10	3	3000	Bachelor	Master or	Orthodox	Georgian	City/Town
Low Risk	1	26	Male	No	Exposure B	90	400	400	4000	Master or	High Schoo	Orthodox	Mixed	City/Town
Low Risk	0	27	Female	Yes	Exposure B	0	360	270	1000	High Schoo	High Schoo	Muslim	Georgian	City/Town
Low Risk	0	28	Female	No	Exposure B	60	60	60	8000	PhD	PhD	Orthodox	Georgian	City/Town
Low Risk	1	20	Male	No	Exposure B	30	20	15	3000	Master or	Master or	Orthodox	Georgian	City/Town
Low Risk	0	25	Female	No	Exposure B	60	60	60	1500	High Schoo	Master or	Orthodox	Georgian	City/Town
High Risk	12	17	Male	No	Not Expose	20	90	30	1600	Bachelor	Bachelor	Orthodox	Georgian	City/Town
Low Risk	1	22	Male	Yes	Exposure B	30	30	90	1500	Bachelor	Bachelor	Other	Georgian	Village
High Risk	9	30	Male	No	Exposure B	90	90	90	0	High Schoo	High Schoo	Orthodox	Mixed	Emigrant (West)
Low Risk	0	18	Female	Yes	Exposure B	30	90	90	3000	High Schoo	Bachelor	Orthodox	Georgian	City/Town
Medium Ri	3	30	Male	Yes	Exposure B	180	90	60	3000	High Schoo	High Schoo	Orthodox	Georgian	City/Town
Low Risk	0	22	Female	No	Exposure B	45	180	90	1500	Bachelor	Bachelor	Orthodox	Georgian	City/Town
Low Risk	1	27	Male	Yes	Exposure B	0	360		500	Bachelor	Bachelor	Orthodox	Georgian	City/Town
Low Risk	2	26	Male	No	Exposure B	30	90	180	1500	Bachelor	High Schoo	Orthodox	Georgian	City/Town
Low Risk	0	17	Female	No	Not Expose	30	30	30	3000	High Schoo	High Schoo	Orthodox	Georgian	City/Town
Low Risk	1	21	Female	No	Exposure B	15	10	5	1500	Bachelor	High Schoo	Orthodox	Georgian	City/Town
Low Risk	0	21	Male	No	Exposure B	30	30	90	1500	Bachelor	High Schoo	Orthodox	Georgian	City/Town
Low Risk	0	18	Female	No	Exposure B	20	20	0	3000	Master or	Bachelor	Orthodox	Georgian	City/Town
Low Risk	0	18	Female	No	Exposure B	20	20	0	3000	Master or	Bachelor	Orthodox	Georgian	City/Town
Low Risk	0	19	Female	No	Not Expose	0	0	0	1800	Master or	High Schoo	Orthodox	Georgian	City/Town
Low Risk	0	24	Male	No	Exposure B	180	180	180	2000	High Schoo	High Schoo	Orthodox	Georgian	City/Town
Low Risk	0	24	Male	Yes	Exposure B	90	90	90		Bachelor	Bachelor	Orthodox	Georgian	City/Town
Low Risk	0	17	Male	No	Exposure B	5	5	0	2000	Bachelor	Master or	Orthodox	Georgian	City/Town
Low Risk	0	24	Female	Yes	Exposure B	90	90	90	3000	Bachelor	High Schoo	Orthodox	Georgian	Emigrant (West)
Medium Ri	7	30	Male	Yes	Exposure B	360	360	360	3000	Middle Sch	Middle Sch	Orthodox	Georgian	City/Town
Medium Ri	3	23	Male	Yes	Exposure B	90	90	90	10000	Master or	High Schoo	Orthodox	Georgian	City/Town
Low Risk	0	18	Female	Yes	Exposure B	60	60	0	4000	Master or	Master or	Orthodox	Georgian	City/Town
Low Risk	2	24	Male	Yes	Exposure B	90	90	90	1500	Bachelor	Bachelor	Orthodox	Georgian	City/Town
Medium Ri	7	24	Male	Yes	Exposure B	180	360	360	1500	Bachelor	Bachelor	Orthodox	Georgian	City/Town
Medium Ri	3	18	Male	Yes	Exposure B	360	360	180	0	Master or	Master or	Orthodox	Georgian	City/Town
Low Risk	0	29	Male	No	Exposure B	5	60	300	3500	Bachelor	Bachelor	Orthodox	Georgian	City/Town
Low Risk	0	24	Female	Yes	Exposure B	90	90	30	6000	Bachelor	Bachelor	Orthodox	Georgian	City/Town
Low Risk	1	24	Female	No	Exposure B	30	10	20	3000	Bachelor	Bachelor	Orthodox	Georgian	City/Town
Low Risk	0	18	Male	Yes	Exposure B	180	180	180	1500	Bachelor	Master or	Orthodox	Georgian	City/Town
High Risk	9	24	Male	Yes	Exposure B	90	90	360	3000	High Schoo	Middle Sch	Orthodox	Georgian	City/Town
Low Risk	2	19	Male	Yes	Exposure B	40	60	60	1000	High Schoo	High Schoo	Orthodox	Georgian	City/Town

Low Risk	0	22	Male	Yes	Exposure B	30	30	20	3000	Master or I	High Schoo	Orthodox	Georgian	City/Town
Low Risk	0	20	Female	No	Exposure B	30	30	30	5000	Bachelor	PhD	Orthodox	Georgian	City/Town
Medium Ri	5	28	Male	No	Exposure B	90	360	560	5000	Master or I	Bachelor	Orthodox	Georgian	City/Town
Low Risk	1	17	Male	Yes	Exposure B	90	60	30	6000	Bachelor	Master or I	Orthodox	Georgian	City/Town
Low Risk	0	21	Male	Yes	Exposure B	360	360	360	1500	High Schoo	High Schoo	Orthodox	Georgian	City/Town
Low Risk	1	18	Male	Yes	Exposure B	90	90	90	2000	High Schoo	Bachelor	Muslim	Azerbaijan	City/Town
High Risk	9	30	Male	Yes	Exposure B	30	360	640		Bachelor	High Schoo	Orthodox	Georgian	City/Town
Low Risk	0	19	Male	No	Not Expose	0	0	15	4400	Master or I	PhD	Orthodox	Georgian	City/Town
Low Risk	1	21	Male	No	Exposure B	30	180	360	2000	High Schoo	High Schoo	Orthodox	Georgian	City/Town
Low Risk	0	19	Female	No	Exposure B	15	90	120	7000	Master or I	Bachelor	Orthodox	Georgian	City/Town
Low Risk	1	17	Female	Yes	Exposure B	10	0	0	1500	Bachelor	High Schoo	Orthodox	Georgian	Village
Low Risk	2	29	Male	Yes	Exposure B	20	20	20	500	Bachelor	High Schoo	Orthodox	Georgian	Village
High Risk	15	30	Male	Yes	Exposure B	180	180	180	1500	Bachelor	Bachelor	Orthodox	Georgian	City/Town
Low Risk	1	26	Male	Yes	Exposure B	180	240	180	4000	Bachelor	Bachelor	Orthodox	Georgian	City/Town
Low Risk	0	18	Female	No	Not Expose	20	20	20	15000	High Schoo	High Schoo	Orthodox	Georgian	City/Town
Low Risk	0	25	Female	No	Exposure B	15	10	16	6000	High Schoo	High Schoo	Orthodox	Georgian	Village
Low Risk	0	22	Male	Yes	Exposure B	60	600	800	2500	High Schoo	High Schoo	Orthodox	Georgian	City/Town
Low Risk	1	19	Male	No	Not Expose	0	0	0	2000	Middle Sch	Middle Sch	Orthodox	Georgian	City/Town
Low Risk	1	21	Male	No	Exposure B	40	30	20	3000	Bachelor	Bachelor	Orthodox	Georgian	Village
Medium Ri	4	21	Male	Yes	Exposure B	0	120	90	2300	Bachelor	High Schoo	Orthodox	Georgian	City/Town
Low Risk	1	20	Male	Yes	Exposure B	60	60	90	2000	Bachelor	Bachelor	Orthodox	Georgian	City/Town
Low Risk	1	26	Male	Yes	Exposure B	90	90	90	3000	High Schoo	High Schoo	Orthodox	Georgian	Village
Low Risk	0	26	Male	Yes	Exposure B	90	90	90	3000	High Schoo	High Schoo	Orthodox	Georgian	Village
Low Risk	1	18	Male	No	Exposure B	30	20	20		Bachelor	Bachelor	Orthodox	Georgian	City/Town
Low Risk	0	24	Male	Yes	Exposure B	90	90	90	3000	High Schoo	High Schoo	Orthodox	Georgian	Mountainous Region
Low Risk	0	20	Male	No	Exposure B	30	30	10	4500	Bachelor	Bachelor	Orthodox	Georgian	City/Town
Low Risk	1	18	Female	No	Not Expose	5	15	5	150	Bachelor	Bachelor	Orthodox	Georgian	City/Town
Low Risk	0	29	Male	Yes	Exposure B	90	30	30	1500	Bachelor	Bachelor	Orthodox	Georgian	City/Town
Medium Ri	5	30	Female	Yes	Exposure B	60	30	30	2000	Bachelor	Bachelor	Orthodox	Georgian	City/Town
Medium Ri	3	19	Male	Yes	Exposure B	0	0	0	3000	High Schoo	Bachelor	Orthodox	Georgian	Village
Low Risk	0	28	Male	No	Exposure B	18	120	300	3300	Bachelor	Bachelor	Orthodox	Georgian	City/Town
High Risk	8	17	Male	Yes	Exposure B	420	420	420	5000	Bachelor	Bachelor	Orthodox	Georgian	City/Town
Low Risk	0	17	Male	No	Not Expose	0	0	0	5000	Master or I	Master or I	Orthodox	Georgian	City/Town
Low Risk	0	25	Female	No	Exposure B	30	180	360	3000	Master or I	Master or I	Orthodox	Georgian	City/Town
Low Risk	1	18	Female	Yes	Exposure B	15	90	180	400	High Schoo	High Schoo	Orthodox	Georgian	Mountainous Region
Low Risk	2	27	Female	Yes	Exposure B	180	180	180	1000	Master or I	Master or I	Orthodox	Georgian	City/Town
Low Risk	2	22	Male	Yes	Exposure B	90	90	90	1000	Bachelor	Bachelor	Orthodox	Georgian	City/Town
Low Risk	0	26	Male	Yes	Exposure B	30	30	30	1500	High Schoo	High Schoo	Orthodox	Georgian	City/Town
Low Risk	2	18	Male	Yes	Exposure B	1	7	10	6000	High Schoo	High Schoo	Orthodox	Georgian	City/Town
Medium Ri	3	25	Female	Yes	Exposure B	60	240	240	3000	Bachelor	High Schoo	Orthodox	Georgian	City/Town
Low Risk	2	27	Male	No	Exposure B	90	90	180	1000	High Schoo	High Schoo	Orthodox	Georgian	City/Town
Low Risk	0	25	Female	Yes	Exposure B	60	60	60	2000	Bachelor	High Schoo	Orthodox	Georgian	City/Town
Low Risk	0	21	Female	No	Not Expose	0	0	0	4500	Bachelor	High Schoo	Orthodox	Georgian	Village
Low Risk	0	24	Male	Yes	Exposure B	40	30	30	2000	Bachelor	High Schoo	Orthodox	Georgian	Village

Low Risk	0	18	Female	No	Not Expose	20	15	15	3000	Bachelor	Bachelor	Orthodox	Georgian	City/Town
High Risk	10	29	Female	No	Not Expose	180	180	180	1500	High Schoo	High Schoo	Orthodox	Georgian	City/Town
Low Risk	1	23	Male	Yes	Exposure B	30	300	600	3000	Master or I	Bachelor	Orthodox	Georgian	City/Town
Medium Ri	3	27	Female	Yes	Exposure B	360	360	360	1000	Master or I	Bachelor	Orthodox	Georgian	City/Town
Medium Ri	3	24	Male	Yes	Exposure B	30	30	30	5000	Master or I	Bachelor	Orthodox	Georgian	City/Town
Low Risk	0	22	Male	Yes	Exposure B	90	90	30	5000	Master or I	High Schoo	Orthodox	Georgian	City/Town
Low Risk	0	26	Female	No	Exposure B	90	180	360	2000	Bachelor	Bachelor	Orthodox	Georgian	City/Town
Low Risk	0	21	Female	Yes	Exposure B	90	90	90	3000	Bachelor	Bachelor	Orthodox	Georgian	City/Town
Low Risk	1	27	Male	No	Exposure B	90	30	90	3500	Bachelor	Bachelor	Orthodox	Georgian	City/Town
High Risk	11	30	Male	No	Not Expose	90	90	500	2000	Bachelor	High Schoo	Other	Mixed	City/Town
High Risk	9	24	Female	No	Exposure B	180	360	360	500	High Schoo	High Schoo	Orthodox	Georgian	City/Town
Low Risk														
Medium Ri	3	30	Male	Yes	Exposure B	90	90	180	3000	High Schoo	High Schoo	Orthodox	Georgian	City/Town
Medium Ri	4	17	Male	Yes	Exposure B	20	20	20	1500	Bachelor	High Schoo	Orthodox	Georgian	City/Town
Low Risk	2	18	Female	No	Not Expose	0	0	0	3000	Bachelor	High Schoo	Orthodox	Georgian	City/Town
Low Risk	0	24	Female	No	Exposure B	60	60	30	4000	Bachelor	Bachelor	Orthodox	Georgian	City/Town
Low Risk	1	29	Male	No	Not Expose	0	25	120	1100	Bachelor	Bachelor	Orthodox	Georgian	City/Town
Low Risk	0	22	Female	Yes	Exposure B	180	180	180	1500	High Schoo	High Schoo	Orthodox	Georgian	City/Town
Medium Ri	3	19	Male	Yes	Exposure B	30	30	30	3000	Bachelor	Bachelor	Orthodox	Georgian	City/Town
Low Risk	0	29	Female	No	Exposure B	60	60	60	6000	Bachelor	Bachelor	Orthodox	Georgian	City/Town
Low Risk	1	24	Female	No	Exposure B	30	90	90	15000	Middle Sch	High Schoo	Orthodox	Georgian	City/Town
Low Risk	1	21	Female	No	Exposure B	20	90	120	6000	Bachelor	Bachelor	Orthodox	Georgian	City/Town
Low Risk	0	24	Female	No	Exposure B	90	90	180	1500	Middle Sch	High Schoo	Orthodox	Georgian	City/Town
Low Risk	0	22	Female	Yes	Exposure B	5	10	10	1500	Bachelor	Bachelor	Orthodox	Georgian	City/Town
Low Risk	0	17	Male	No	Not Expose	10	0	0	3000	Bachelor	Bachelor	Orthodox	Georgian	City/Town
Low Risk	0	19	Male	No	Not Expose	0	36	36	3000	Master or I	Bachelor	Orthodox	Georgian	City/Town
Low Risk	0	28	Female	No	Exposure B	90	90	30	3000	Bachelor	High Schoo	Orthodox	Georgian	Village
Low Risk	1	17	Female	Yes	Exposure B	90	90	90	5000	Master or I	Bachelor	Orthodox	Georgian	City/Town
Low Risk	0	18	Male	No	Not Expose	0	0	0	5500	Master or I	Bachelor	Orthodox	Georgian	City/Town
Low Risk	1	21	Male	Yes	Exposure B	30	50	30	2000	Bachelor	Bachelor	Orthodox	Georgian	City/Town
Low Risk	1	24	Male	Yes	Exposure B	30	180	360	1500	Bachelor	Bachelor	Orthodox	Georgian	City/Town
Low Risk	0	23	Female	No	Exposure B	15	30	60	2500	Bachelor	High Schoo	Orthodox	Georgian	Village
Low Risk	1	19	Female	Yes	Exposure B	90	90	30	100	Bachelor	High Schoo	Orthodox	Georgian	City/Town
Low Risk	0	23	Male	No	Exposure B	1	1	1	2000	Middle Sch	Bachelor	Orthodox	Georgian	City/Town
Medium Ri	5	23	Male	No	Exposure B	30	180	360	6000	Master or I	Master or I	Orthodox	Georgian	City/Town
Low Risk	0	28	Male	No	Exposure B	45	30	30	5000	Master or I	Master or I	Orthodox	Georgian	City/Town
Low Risk	2	18	Male	Yes	Exposure B	30	90	180	5000	Master or I	Master or I	Orthodox	Georgian	City/Town
Low Risk	0	20	Male	Yes	Exposure B	20	50	20	8000	Bachelor	Bachelor	Orthodox	Georgian	City/Town
Low Risk	0	26	Male	Yes	Exposure B	120	120	120	1000	Bachelor	High Schoo	Orthodox	Georgian	Village
Medium Ri	5	24	Male	No	Exposure B	30	20	30	1500	High Schoo	High Schoo	Orthodox	Other	Mountainous Region
Low Risk	1	17	Female	No	Exposure B	15	15	15	1500	Master or I	Bachelor	Orthodox	Georgian	Village
Low Risk	2	17	Male	No	Not Expose	0	0	0	1500	Bachelor	Bachelor	Orthodox	Georgian	Emigrant (West)
Low Risk	1	18	Male	No	Exposure B	5	5	5		Bachelor	Bachelor	Atheist	Georgian	City/Town
Medium Ri	3	18	Male	No	Exposure B	300	180	90	1200	Master or I	Master or I	Orthodox	Georgian	City/Town

Low Risk	0	18	Female	No	Exposure B	10	10	10	2500	High Schoo	Bachelor	Orthodox	Georgian	City/Town
Low Risk	0	20	Female	No	Not Expose	0	0	0		Bachelor	Bachelor	Orthodox	Georgian	Village
Low Risk	0	24	Female	Yes	Exposure B	60	60	60	6000	High Schoo	High Schoo	Orthodox	Georgian	Village
Low Risk	2	24	Male	Yes	Exposure B	90	90	90	500		Bachelor	Orthodox	Georgian	Mountainous Region
Low Risk	1	24	Female	Yes	Exposure B	360	360	360	2500	High Schoo	Bachelor	Orthodox	Russian	Village
Medium Ri	4	30	Female	Yes	Exposure B	90	60	60	5000	Master or	Master or	Orthodox	Georgian	Emigrant (Asia/Russia/Africa)
Low Risk	0	24	Male	Yes	Exposure B	15	30	50	1500	Bachelor	High Schoo	Orthodox	Georgian	Village
Low Risk	0	20	Female	No	Exposure B	120	120	120	1500	High Schoo	High Schoo	Muslim	Georgian	City/Town
Medium Ri	4	17	Male	No	Exposure B	180	240	90	5000	Master or	Master or	Orthodox	Georgian	City/Town
Low Risk	1	22	Male	No	Not Expose	0	0	0	500	Bachelor	Bachelor	Orthodox	Georgian	City/Town
Low Risk	0	21	Male	No	Exposure B	10	40	40	0	Bachelor	Bachelor	Orthodox	Georgian	Emigrant (West)
Low Risk	1	23	Female	No	Exposure B	60	180	360	8000	Bachelor	Bachelor	Orthodox	Georgian	Village
Low Risk														
Low Risk	0	25	Male	No	Exposure B	60	60	60	3500	Master or	High Schoo	Orthodox	Georgian	City/Town
Low Risk	0	17	Male	Yes	Exposure B	30	90	180	1500	Bachelor	Bachelor	Orthodox	Georgian	City/Town
Low Risk	0	18	Male	Yes	Exposure B	18	5	10	1500	High Schoo	High Schoo	Orthodox	Georgian	City/Town
Low Risk	0	23	Female	No	Not Expose	90	90	90	2000	Bachelor	High Schoo	Orthodox	Georgian	City/Town
Low Risk	1	28	Female	Yes	Exposure B	90	90	90	5000	Bachelor	High Schoo	Orthodox	Georgian	City/Town
Low Risk	1	26	Male	No	Exposure B	0	10	10	2500	Master or	High Schoo	Orthodox	Georgian	City/Town
Low Risk	0	23	Male	No	Exposure B	30	30	30	3000	Bachelor	Bachelor	Orthodox	Georgian	Village
Low Risk	0	18	Male	Yes	Exposure B	30	30	20	2000	Bachelor	High Schoo	Orthodox	Georgian	City/Town
Low Risk	0	20	Male	No	Not Expose	0	0	0	5000	Bachelor	High Schoo	Orthodox	Georgian	Village
Medium Ri	7	19	Male	Yes	Exposure B	120	120	120	3000	Bachelor	Bachelor	Orthodox	Georgian	Mountainous Region
Low Risk	0	26	Female	Yes	Exposure B	40	30	30	3000	Master or	Master or	Orthodox	Georgian	City/Town
Low Risk	0	19	Male	No	Not Expose	0	0	0	3000	Master or	Master or	Orthodox	Georgian	City/Town
Medium Ri	5	30	Male	Yes	Exposure B	30	360	360	3000	Master or	Master or	Orthodox	Georgian	City/Town
Low Risk	1	24	Female	Yes	Exposure B	90	90	90	2750	Bachelor	High Schoo	Orthodox	Georgian	City/Town
Low Risk	2	29	Male	Yes	Exposure B	90	90	90	5000	Bachelor	Bachelor	Orthodox	Georgian	City/Town
Low Risk	0	18	Male	Yes	Exposure B	180	180	180	2500	Middle Sch	Middle Sch	Orthodox	Armenian	Emigrant (West)
Low Risk	2	22	Male	Yes	Exposure B	30	600	240	2000	Bachelor	High Schoo	Orthodox	Georgian	City/Town
Low Risk														
Low Risk	1	24	Male	Yes	Exposure B	90	90	80	5000	High Schoo	High Schoo	Orthodox	Georgian	Village
Low Risk	1	21	Female	No	Exposure B	10	60	150	1500	Bachelor	Middle Sch	Orthodox	Georgian	City/Town
Low Risk	1	17	Male	Yes	Exposure B	30	30	30	2000	Master or	Bachelor	Orthodox	Georgian	City/Town
Low Risk	0	24	Male	Yes	Exposure B	360	360	360	2500	High Schoo	Bachelor	Orthodox	Georgian	City/Town
Low Risk	0	27	Female	No	Exposure B	15	90	90	5000	Master or	Bachelor	Orthodox	Georgian	City/Town
Low Risk	0	21	Female	No	Exposure B	30	180	180	4000	Bachelor	Bachelor	Orthodox	Georgian	City/Town
Medium Ri	3	18	Female	No	Not Expose	0	0	0	2500	Bachelor	High Schoo	Orthodox	Georgian	City/Town
Low Risk	0	17	Male	No	Not Expose	10	60	100	10000	Master or	Master or	Orthodox	Georgian	City/Town
Low Risk	0	25	Female	No	Exposure B	10	80	180	1500	Bachelor	Bachelor	Orthodox	Georgian	City/Town
High Risk	8	30	Male	Yes	Exposure B	45	90	90	3000	Bachelor	Bachelor	Orthodox	Georgian	Mountainous Region
Low Risk	0	21	Female	No	Not Expose	15	5	5	3000	Bachelor	Bachelor	Orthodox	Georgian	City/Town
Low Risk	0	24	Male	No	Exposure B	30	6	360	1500	Bachelor	Bachelor	Orthodox	Georgian	City/Town
Low Risk	0	19	Male	Yes	Exposure B	90	90	30	1500	Bachelor	High Schoo	Orthodox	Georgian	City/Town

Low Risk	0	22	Male	Yes	Exposure B	30	10	15	2000	Middle Sch	Middle Sch	Orthodox	Georgian	City/Town
Low Risk	0	25	Male	No	Exposure B	90	30	30	1000	Bachelor	Bachelor	Orthodox	Georgian	City/Town
Low Risk	0	28	Male	Yes	Exposure B	100	100	90	5000	Bachelor	Bachelor	Orthodox	Georgian	City/Town
Low Risk	1	22	Male	No	Exposure B	30	400	500	2000	Bachelor	High Schoo	Orthodox	Armenian	City/Town
Low Risk	0	18	Male	No	Not Expose	0	0	0	1500	High Schoo	High Schoo	Orthodox	Georgian	City/Town
Low Risk	0	28	Female	Yes	Exposure B	30	180	200	3000	Bachelor	Bachelor	Orthodox	Georgian	City/Town
Low Risk	0	18	Male	No	Not Expose	15	10	10	2500	Bachelor	High Schoo	Orthodox	Georgian	City/Town
Low Risk	0	18	Female	No	Not Expose	0	0	0	3000	Bachelor	Bachelor	Orthodox	Georgian	City/Town
Low Risk	0	24	Female	Yes	Exposure B	30	20	15	2500	High Schoo	High Schoo	Orthodox	Georgian	City/Town
Low Risk	0	24	Female	No	Exposure B	30	10	15	2500	High Schoo	High Schoo	Orthodox	Georgian	City/Town
Low Risk	1	25	Male	No	Exposure B	65	55	40	6000	Master or I	Bachelor	Atheist	Mixed	Emigrant (West)
Low Risk	0	22	Male	No	Exposure B	15	0	0	0	Bachelor	Master or I	Orthodox	Georgian	City/Town
Low Risk	1	22	Female	Yes	Exposure B	5	5	5	1500	Bachelor	Master or I	Orthodox	Georgian	Emigrant (West)
Low Risk	1	24	Male	No	Exposure B	90	400	360	3000	Bachelor	High Schoo	Orthodox	Georgian	City/Town
Medium Ri	7	24	Male	No	Not Expose	90	180	180	3000	High Schoo	High Schoo	Orthodox	Georgian	Emigrant (West)
Medium Ri	3	22	Female	Yes	Exposure B	90	90	90	3000	Bachelor	High Schoo	Orthodox	Georgian	City/Town
Low Risk	1	24	Male	No	Exposure B	90	360	500	2000	Bachelor	Bachelor	Orthodox	Georgian	City/Town
Low Risk	1	19	Female	Yes	Exposure B	10	30	30		Bachelor	Bachelor	Orthodox	Mixed	City/Town
Low Risk	0	21	Female	No	Exposure B	15	15	15	2500	Bachelor	Bachelor	Orthodox	Georgian	City/Town
Low Risk	1	23	Female	No	Exposure B	10	60	10	5000	Master or I	Master or I	Orthodox	Georgian	City/Town
Low Risk	0	21	Male	No	Exposure B	90	60	60	20000	Master or I	Master or I	Orthodox	Georgian	City/Town
Low Risk	0	18	Male	No	Not Expose	15	0	15	3500	Bachelor	Bachelor	Orthodox	Georgian	City/Town
Low Risk	2	24	Female	Yes	Exposure B	30	180	360	1500	Bachelor	Bachelor	Orthodox	Georgian	City/Town
Low Risk	1	25	Male	No	Not Expose	20	30	180	3000	Bachelor	Bachelor	Orthodox	Georgian	City/Town
Low Risk	1	24	Male	Yes	Exposure B	90	90	180	3000	High Schoo	Middle Sch	Orthodox	Georgian	City/Town
Low Risk	0	23	Female	Yes	Exposure B	90	90			Bachelor	Bachelor	Orthodox	Georgian	City/Town
Low Risk	1	25	Male	No	Not Expose	5	5	5	5000	Master or I	Bachelor	Orthodox	Georgian	City/Town
Low Risk	1	18	Female	Yes	Exposure B	30	360	360	1500	Bachelor	Bachelor	Orthodox	Georgian	City/Town
Low Risk	0	21	Male	No	Not Expose	30	30	30	2000	Bachelor	Bachelor	Orthodox	Georgian	City/Town
Low Risk	1	19	Male	No	Not Expose	5	10	5	1000	Bachelor	Bachelor	Orthodox	Georgian	City/Town
Medium Ri	6	18	Male	No	Exposure B	30	180	360	4000	High Schoo	High Schoo	Orthodox	Georgian	Village
Low Risk	1	24	Male	Yes	Exposure B	70	90	40	2200	High Schoo	High Schoo	Orthodox	Georgian	Village
Low Risk	0	28	Male	No	Not Expose	0	0	0	0	Master or I	Bachelor	Orthodox	Georgian	City/Town
Low Risk	0	27	Male	No	Exposure B	30	90	180	7000	Master or I	Master or I	Orthodox	Georgian	Emigrant (West)
High Risk	8	30	Male	No	Exposure B	90	90	90	5000	Master or I	Master or I	Orthodox	Georgian	City/Town
Low Risk	0	18	Male	Yes	Exposure B	40	180	30	2000	Bachelor	High Schoo	Orthodox	Georgian	City/Town
Low Risk	0	24	Male	Yes	Exposure B	18	20	15	4000	Master or I	Bachelor	Orthodox	Georgian	City/Town
Low Risk	0	21	Female	No	Exposure B	30	180	400	2000	Bachelor	Bachelor	Orthodox	Georgian	City/Town
Low Risk	1	21	Male	No	Exposure B	15	15	30	2000	Bachelor	Bachelor	Orthodox	Georgian	Emigrant (West)
Low Risk														
Low Risk	2	21	Male	Yes	Exposure B	5	5	10	3000	High Schoo	High Schoo	Orthodox	Georgian	City/Town
Medium Ri	5	25	Female	Yes	Exposure B	90	90	90	1500	Master or I	Bachelor	Orthodox	Georgian	City/Town
Medium Ri	7	26	Male	Yes	Exposure B	90	90	90	2000	High Schoo	High Schoo	Orthodox	Georgian	Village
Low Risk	0	19	Male	No	Not Expose	0	0	0	3000	Bachelor	High Schoo	Orthodox	Georgian	City/Town

Low Risk	0	20	Male	No	Exposure B	40	30	40	3000	Master or	Bachelor	Orthodox	Georgian	City/Town
Medium Ri	5	28	Female	Yes	Exposure B	90	360	550	1200	Bachelor	Bachelor	Orthodox	Georgian	City/Town
Low Risk	0	27	Male	Yes	Exposure B	180	180	180	2000	Bachelor	Bachelor	Orthodox	Georgian	City/Town
Medium Ri	5	17	Male	Yes	Exposure B	30	180	18	3000	High Schoo	Bachelor	Orthodox	Georgian	City/Town
Medium Ri	5	17	Male	Yes	Exposure B	30	180	18	3000	High Schoo	Bachelor	Orthodox	Georgian	City/Town
Low Risk	1	17	Male	Yes	Exposure B	30	30	30	1500	Master or	Master or	Orthodox	Georgian	Village
Low Risk	0	17	Female	Yes	Exposure B	15	180	90	1500	Bachelor	High Schoo	Orthodox	Georgian	City/Town
Low Risk	0	23	Male	No	Exposure B	90	30	20	3500	Master or	Bachelor	Orthodox	Georgian	City/Town
Low Risk	0	17	Female	No	Not Expose	0	0	0	2000	Master or	High Schoo	Orthodox	Georgian	Village
Low Risk	1	21	Female	Yes	Exposure B	90	90	90	5000	Master or	Master or	Orthodox	Georgian	City/Town
Low Risk	0	19	Male	No	Not Expose	0	0	0	2000	High Schoo	High Schoo	Orthodox	Georgian	City/Town
Low Risk	0	23	Female	No	Exposure B	90	30	30	5000	Master or	PhD	Orthodox	Georgian	City/Town
Low Risk	0	18	Male	No	Exposure B	20	360	360	4000	Master or	Master or	Orthodox	Georgian	City/Town
Medium Ri	4	30	Female	Yes	Exposure B	360	360	360	1500	Middle Sch	Middle Sch	Orthodox	Georgian	Emigrant (West)
Low Risk	0	19	Male	Yes	Exposure B	30	15	5	1000	Bachelor	High Schoo	Orthodox	Georgian	Village
Medium Ri	3	17	Male	Yes	Exposure B	30	30	30	3000	Bachelor	Bachelor	Orthodox	Georgian	Village
High Risk	9	30	Male	No	Exposure B	30	30	30	2000	High Schoo	High Schoo	Catholic	Mixed	Emigrant (West)
Low Risk	1	19	Male	Yes	Exposure B	60	30	20	3000	Bachelor	Bachelor	Orthodox	Georgian	City/Town
Low Risk	0	19	Male	No	Exposure B	8	8	8	2500	Bachelor	Bachelor	Orthodox	Georgian	City/Town
Low Risk	1	18	Female	Yes	Exposure B	90	180	200	7000	Bachelor	Bachelor	Orthodox	Georgian	City/Town
Low Risk	0	18	Female	Yes	Exposure B	15	30	30	5000	Master or	Master or	Orthodox	Georgian	City/Town
Medium Ri	6	28	Male	No	Exposure B	90	180	30	1500	Bachelor	High Schoo	Orthodox	Georgian	City/Town
Low Risk	0	18	Male	No	Exposure B	8	200	290	0	Bachelor	Bachelor	Orthodox	Georgian	City/Town
Low Risk	1	22	Female	Yes	Exposure B	15	15	5	6000	Master or	Master or	Orthodox	Georgian	City/Town
Low Risk	0	17	Female	No	Not Expose	5	30	40	1000	High Schoo	High Schoo	Orthodox	Georgian	City/Town
Low Risk	0	24	Male	No	Exposure B	10	50	80	2500	Master or	Master or	Orthodox	Georgian	City/Town
Low Risk	0	17	Female	No	Not Expose	5	30	40	1000	High Schoo	High Schoo	Orthodox	Georgian	City/Town
Low Risk	1	21	Male	No	Exposure B	0	30	90	3000	Master or	Bachelor	Orthodox	Georgian	City/Town
Low Risk	0	23	Female	No	Exposure B	20	10	3	5000	Master or	Master or	Orthodox	Georgian	City/Town
Low Risk	0	19	Male	No	Exposure B	30	30	30	3000	Bachelor	Bachelor	Orthodox	Georgian	Village
Low Risk	0	20	Female	Yes	Exposure B	20	30	90	3000	High Schoo	High Schoo	Orthodox	Georgian	City/Town
Low Risk	2	24	Male	Yes	Exposure B	20	30	90	1500	High Schoo	High Schoo	Orthodox	Georgian	Village
Medium Ri	3	21	Female	No	Exposure B	60	90	90	1800	Bachelor	Bachelor	Muslim	Georgian	City/Town
Low Risk	2	25	Male	Yes	Exposure B	105	105	105	2000	High Schoo	High Schoo	Orthodox	Georgian	Village
Medium Ri	3	18	Female	Yes	Exposure B	360	360	700	1500	Middle Sch	Middle Sch	Orthodox	Russian	Emigrant (West)
Low Risk	0	24	Male	No	Exposure B	15	60	90	3000	Bachelor	Middle Sch	Orthodox	Georgian	Village
Medium Ri	3	21	Male	Yes	Exposure B	30	30	30	1000	High Schoo	High Schoo	Orthodox	Georgian	Mountainous Region
Low Risk	0	19	Male	Yes	Exposure B	90	60	30	4000	Bachelor	High Schoo	Orthodox	Georgian	City/Town
Low Risk	0	26	Male	Yes	Exposure B	90	90	90	4000	High Schoo	Master or	Orthodox	Georgian	Village
Low Risk	2	27	Male	No	Exposure B	10	10	15	2000	Master or	Bachelor	Orthodox	Georgian	City/Town
Medium Ri	3	22	Female	Yes	Exposure B	180	180	180	400	High Schoo	High Schoo	Orthodox	Georgian	Village
Low Risk	0	27	Female	No	Exposure B	30	360	500	7000	Bachelor	Bachelor	Orthodox	Georgian	City/Town
Low Risk	0	29	Male	No	Exposure B	60	30	20	5000	Bachelor	Bachelor	Orthodox	Georgian	City/Town
Medium Ri	6	27	Female	Yes	Exposure B	90	30	90	1500	Middle Sch	High Schoo	Orthodox	Georgian	City/Town

Medium Ri	7	30	Female	Yes	Exposure B	120	40	60	500	High Schoo	High Schoo	Orthodox	Georgian	Village
Low Risk	1	17	Male	Yes	Exposure B	30	30	30	1500	Master or I	High Schoo	Orthodox	Georgian	City/Town
Medium Ri	3	20	Female	No	Exposure B	90	90	360	4000	High Schoo	Master or I	Orthodox	Georgian	City/Town
Low Risk	0	17	Male	No	Exposure B	30	30	30	1500	Bachelor	High Schoo	Orthodox	Georgian	Village
Low Risk	1	18	Male	No	Exposure B	30	30	30	3000	Bachelor	Bachelor	Orthodox	Georgian	City/Town
Low Risk	1	24	Male	Yes	Exposure B	60	90	90	3000	Bachelor	Bachelor	Orthodox	Georgian	City/Town
Medium Ri	3	18	Female	No	Exposure B	180	180	180	4000	Bachelor	Bachelor	Orthodox	Georgian	City/Town
Low Risk	0	18	Female	Yes	Exposure B	360	360	360	1500	High Schoo	High Schoo	Orthodox	Georgian	City/Town
Low Risk	1	20	Female	Yes	Exposure B	60	60	60	3500	High Schoo	Bachelor	Orthodox	Georgian	City/Town
Low Risk	0	25	Male	No	Exposure B	90	90	30	1500	Bachelor	High Schoo	Orthodox	Georgian	City/Town
High Risk	14	30	Male	Yes	Exposure B	360	500	500	1200	Middle Sch	Middle Sch	Orthodox	Georgian	City/Town
Low Risk	0	24	Female	No	Exposure B	90	90	90	3000	Master or I	Bachelor	Other	Georgian	City/Town
Medium Ri	3	30	Male	No	Exposure B	30	30	30	1000	Bachelor	High Schoo	Orthodox	Georgian	City/Town
Low Risk	2	27	Male	Yes	Exposure B	30	30	30	2400	Bachelor	Bachelor	Orthodox	Georgian	City/Town
Low Risk	0	22	Male	Yes	Exposure B	90	180	360	3000	Bachelor	Bachelor	Orthodox	Georgian	City/Town
Low Risk	0	29	Female	No	Exposure B	40	30	20	2000	High Schoo	High Schoo	Orthodox	Georgian	City/Town
Low Risk	0	17	Female	No	Exposure B	40	30	20	2000	High Schoo	High Schoo	Orthodox	Georgian	City/Town
Medium Ri	4	20	Male	No	Exposure B	30	30	30	1500	Middle Sch	Bachelor	Orthodox	Georgian	Village
Low Risk	1	20	Male	No	Not Expose	0	20	40	3000	Bachelor	Bachelor	Orthodox	Georgian	City/Town
Low Risk	0	22	Female	No	Exposure B	15	15	15	1500	Bachelor	Bachelor	Orthodox	Georgian	City/Town
High Risk	16	30	Male	Yes	Exposure B	360	500	360	1500	High Schoo	High Schoo	Orthodox	Georgian	City/Town
Low Risk	0	20	Female	No	Not Expose	18	20	15	4000	Master or I	Master or I	Orthodox	Georgian	Village
Medium Ri	3	30	Male	Yes	Exposure B	30	90	90	3000	Master or I	Master or I	Orthodox	Georgian	City/Town
Low Risk	0	22	Female	No	Exposure B	15	30	30	3000	Bachelor	Bachelor	Orthodox	Georgian	City/Town
Low Risk	0	22	Female	No	Exposure B	30	90	90	3000	Bachelor	Bachelor	Orthodox	Georgian	City/Town
Medium Ri	7	21	Female	Yes	Exposure B	40	220	380	5000	Bachelor	High Schoo	Orthodox	Georgian	Emigrant (West)
Low Risk	1	24	Male	No	Not Expose	360	360	180	1500	Bachelor	Bachelor	Orthodox	Georgian	Emigrant (West)
Low Risk	0	26	Female	Yes	Exposure B	30	30	90	500	Bachelor	Bachelor	Orthodox	Georgian	City/Town
Low Risk	0	18	Male	No	Not Expose	0	0	0	8000	Master or I	Master or I	Orthodox	Georgian	City/Town
Low Risk	0	18	Male	No	Not Expose	90	200	200	3000	Master or I	Master or I	Orthodox	Georgian	City/Town
Medium Ri	3	24	Male	No	Not Expose	0	180	180	1500	Bachelor	High Schoo	Orthodox	Georgian	City/Town
Low Risk	0	25	Male	No	Exposure B	60	90	60	3000	High Schoo	Master or I	Orthodox	Georgian	City/Town
Medium Ri	3	24	Male	Yes	Exposure B	180	360	360	6000	Bachelor	Bachelor	Orthodox	Georgian	City/Town
Low Risk	1	19	Female	Yes	Exposure B	30	90	90	7000	High Schoo	High Schoo	Orthodox	Georgian	Emigrant (West)
Low Risk	1	25	Female	Yes	Exposure B	180	180	180	1000	High Schoo	Bachelor	Orthodox	Georgian	City/Town
Low Risk	0	18	Male	Yes	Exposure B	90	60	30	1500	High Schoo	Bachelor	Orthodox	Georgian	Emigrant (West)
Medium Ri	3	22	Male	No	Exposure B	90	60	180	3000	High Schoo	High Schoo	Orthodox	Georgian	Emigrant (West)
Low Risk	0	27	Female	Yes	Exposure B	90	90	90	1500	High Schoo	Middle Sch	Orthodox	Georgian	City/Town
Low Risk	0	29	Female	No	Exposure B	60				Bachelor	High Schoo	Orthodox	Georgian	Village
High Risk	16	30	Male	No	Exposure B	180	360	500	1200	High Schoo	High Schoo	Muslim	Georgian	City/Town
Low Risk	0	20	Female	Yes	Exposure B	15	15	15		Bachelor	High Schoo	Orthodox	Georgian	City/Town
Low Risk	0	24	Male	No	Exposure B	30	360	360	1000	Bachelor	High Schoo	Orthodox	Georgian	City/Town
Low Risk	1	17	Female	Yes	Exposure B	180	180	180	3000	Bachelor	Bachelor	Orthodox	Georgian	City/Town
High Risk	8	23	Male	Yes	Exposure B	360	360	180	2000	Bachelor	Bachelor	Orthodox	Georgian	City/Town

Low Risk	0	17	Male	No	Not Expose	0	0	0	1500	Bachelor	Master or I	Orthodox	Georgian	City/Town
Medium Ri	7	17	Male	Yes	Exposure B	180	180	200	3000	Bachelor	High Schoo	Orthodox	Georgian	Emigrant (West)
Medium Ri	3	19	Female	Yes	Exposure B	120	120	120	4000	Bachelor	Middle Sch	Orthodox	Mixed	City/Town
Low Risk	2	24	Male	Yes	Exposure B	90	90	90	3500	Bachelor	High Schoo	Muslim	Georgian	City/Town
Low Risk	1	19	Female	No	Exposure B	15	90	100	3000	Master or I	Bachelor	Orthodox	Georgian	City/Town
Low Risk	0	24	Female	No	Exposure B	15	30	30	1000	High Schoo	High Schoo	Orthodox	Georgian	City/Town
High Risk	17	20	Female	No	Not Expose	15	0	0	1500	Middle Sch	Bachelor	Orthodox	Georgian	Village
Low Risk	1	20	Male	No	Exposure B	15	0	0	1500	Middle Sch	Bachelor	Orthodox	Georgian	Village
Low Risk	0	18	Female	Yes	Exposure B	90	90	30	5000	Bachelor	Master or I	Orthodox	Georgian	City/Town
Low Risk	0	24	Female	No	Not Expose	5	60	60	3000	Bachelor	High Schoo	Orthodox	Georgian	Village
Low Risk	0	22	Female	Yes	Exposure B	90	90	30	1500	High Schoo	Master or I	Orthodox	Georgian	Village
Medium Ri	3	26	Male	No	Exposure B	30	360	180	3000	Bachelor	Bachelor	Orthodox	Georgian	City/Town
Low Risk	0	25	Male	No	Exposure B	30	30	30	2500	Master or I	Bachelor	Orthodox	Georgian	City/Town
Low Risk	1	26	Female	No	Exposure B	10	10	10	2500	Master or I	Bachelor	Orthodox	Georgian	City/Town
High Risk	12	30	Female	Yes	Exposure B	150	150	150	150	High Schoo	High Schoo	Orthodox	Georgian	City/Town
High Risk	12	30	Female	Yes	Exposure B	150	150	150	150	High Schoo	High Schoo	Orthodox	Georgian	City/Town
Low Risk	0	28	Female	Yes	Exposure B	90	180	360	5000	High Schoo	Master or I	Orthodox	Georgian	City/Town
Low Risk	0	23	Female	Yes	Exposure B	90	90	180	1000	Bachelor	Bachelor	Orthodox	Georgian	City/Town
Low Risk	1	26	Male	No	Not Expose	60	60	60	1500	High Schoo	High Schoo	Orthodox	Georgian	City/Town
Medium Ri	3	30	Male	No	Exposure B	90	90	90	1500	Master or I	Master or I	Muslim	Georgian	City/Town
Low Risk	0	18	Male	No	Exposure B	15	20	10	4000	Bachelor	Bachelor	Orthodox	Georgian	City/Town
Low Risk	0	18	Female	Yes	Exposure B	30	90	180	3000	High Schoo	High Schoo	Orthodox	Georgian	City/Town
High Risk	13	30	Female	Yes	Exposure B	180	90	360	150	Middle Sch	High Schoo	Orthodox	Georgian	City/Town
Medium Ri	3	24	Female	No	Exposure B	30	30	30	15000	Bachelor	Bachelor	Orthodox	Georgian	Village
Low Risk	1	23	Male	No	Not Expose	10	30	180	1900	High Schoo	High Schoo	Orthodox	Georgian	Village
Medium Ri	4	22	Male	No	Not Expose	30	30	360	3000	Master or I	Master or I	Orthodox	Georgian	Emigrant (West)
High Risk	8	24	Female	Yes	Exposure B	30	180	360	500	High Schoo	Bachelor	Orthodox	Georgian	Village
Low Risk	0	25	Male	No	Not Expose	0	0	150	1500	Bachelor	Bachelor	Orthodox	Georgian	City/Town
Medium Ri	4	30	Male	Yes	Exposure B	360	360	360	3000	Master or I	Bachelor	Orthodox	Georgian	City/Town
Low Risk	1	20	Male	Yes	Exposure B	90	180	360	1500	High Schoo	High Schoo	Orthodox	Georgian	City/Town
Low Risk	0	19	Female	Yes	Exposure B	180	180	99	1500	Bachelor	Bachelor	Orthodox	Georgian	Emigrant (West)
Medium Ri	6	21	Male	Yes	Exposure B	360	360	360	2300	Bachelor	High Schoo	Orthodox	Georgian	Mountainous Region
Low Risk	0	19	Male	Yes	Exposure B	180	90	60	1500	Bachelor	Bachelor	Orthodox	Georgian	City/Town
Low Risk	0	29	Male	No	Exposure B	30	30	30	3000	Master or I	Master or I	Orthodox	Georgian	City/Town
Low Risk	0	17	Male	Yes	Exposure B	90	360	360	20000	Master or I	Master or I	Orthodox	Georgian	Emigrant (West)
Low Risk	1	19	Female	Yes	Exposure B	40	30	25	10000	Master or I	Master or I	Atheist	Georgian	City/Town
Low Risk	0	22	Male	No	Not Expose	10	60	180	0	Master or I	Bachelor	Orthodox	Georgian	City/Town
High Risk	11	30	Male	Yes	Exposure B	360	360	180	1500	Middle Sch	Middle Sch	Orthodox	Georgian	Emigrant (West)
Low Risk	0	19	Female	Yes	Exposure B	10	20	30	10000	Master or I	Master or I	Orthodox	Georgian	City/Town
Low Risk	1	29	Male	No	Exposure B	90	90	90	5000	Master or I	Bachelor	Atheist	Ukrainian	City/Town
Low Risk	0	19	Female	No	Not Expose	0	0	0	7000	PhD	Master or I	Orthodox	Georgian	City/Town
Medium Ri	3	24	Female	Yes	Exposure B	30	30	30	1500	Bachelor	High Schoo	Orthodox	Georgian	City/Town
Low Risk														
Low Risk	0	27	Female	No	Exposure B	30	30	40	1500	Bachelor	High Schoo	Orthodox	Armenian	City/Town

Medium Ri	3	18	Female	Yes	Exposure B	60	360		7000	Bachelor	Bachelor	Other	Georgian	City/Town
Low Risk														
Low Risk	0	17	Male	Yes	Exposure B	10	5	10	2000	Bachelor	Middle Sch	Orthodox	Georgian	City/Town
Medium Ri	3	28	Male	Yes	Exposure B	30	30	30	500	Bachelor	Bachelor	Orthodox	Georgian	Village
Medium Ri	3	22	Female	Yes	Exposure B	180	360	360	1500	Bachelor	Bachelor	Orthodox	Georgian	City/Town
Low Risk	0	18	Male	No	Exposure B	30	20	20	3000	Bachelor	Bachelor	Orthodox	Georgian	Emigrant (West)
Low Risk	0	20	Male	No	Exposure B	15	15	10	2000	Master or I	Bachelor	Orthodox	Georgian	City/Town
Low Risk														
Low Risk	1	18	Female	Yes	Exposure B	15	80	180	2000	Master or I	Bachelor	Orthodox	Georgian	City/Town
Low Risk	1	24	Male	No	Exposure B	18	90	90	3000	Bachelor	Master or I	Orthodox	Georgian	City/Town
Low Risk	0	26	Male	No	Not Expose	0	0	0	3000	Master or I	Master or I	Orthodox	Georgian	City/Town
Low Risk	0	21	Female	No	Exposure B	180	180	180	30000	Bachelor	Bachelor	Orthodox	Georgian	Emigrant (West)
Low Risk	1	21	Female	Yes	Exposure B	90	90	90	4000	Master or I	Master or I	Orthodox	Georgian	City/Town
High Risk	11	30	Female	No	Exposure B	15	90	30	1500	High Schoo	Bachelor	Orthodox	Georgian	City/Town
Low Risk	0	18	Male	Yes	Exposure B	90	180	180	3000	Bachelor	High Schoo	Orthodox	Georgian	Village
Low Risk	0	20	Female	No	Exposure B	30	180	90	2300	Bachelor	High Schoo	Orthodox	Georgian	City/Town
Medium Ri	7	24	Female	Yes	Exposure B	360	360	360	3000	High Schoo	High Schoo	Orthodox	Georgian	Village
Medium Ri	6	18	Male	Yes	Exposure B	90	90	30	4000	Bachelor	Master or I	Orthodox	Georgian	City/Town
Medium Ri	6	19	Male	Yes	Exposure B	30	360	360	3000	Master or I	Bachelor	Orthodox	Georgian	Emigrant (West)
Low Risk	0	18	Male	No	Not Expose	0	0	0	0	High Schoo	High Schoo	Orthodox	Georgian	Emigrant (West)
High Risk	13	30	Male	No	Exposure B	18	90	100	600	High Schoo	High Schoo	Orthodox	Georgian	City/Town
Low Risk	1	24	Female	Yes	Exposure B	30	30	30	1500	High Schoo	Master or I	Orthodox	Georgian	City/Town
Low Risk	1	23	Male	Yes	Exposure B	30	0	30	15000	Master or I	High Schoo	Orthodox	Georgian	Emigrant (West)
Low Risk	0	24	Male	Yes	Exposure B	180	360	180	1500	Middle Sch	Bachelor	Orthodox	Georgian	City/Town
Medium Ri	3	19	Male	Yes	Exposure B	30	70	70	1000	Bachelor	Bachelor	Orthodox	Georgian	City/Town
Low Risk	0	23	Male	Yes	Exposure B	60	60	60	8000	Master or I	Bachelor	Orthodox	Mixed	City/Town
Low Risk	2	17	Male	No	Not Expose	15	15	15	3000	Bachelor	Bachelor	Orthodox	Georgian	City/Town
Low Risk	0	17	Female	Yes	Exposure B	30	15	5	500	Bachelor	Bachelor	Orthodox	Georgian	City/Town
Low Risk	2	22	Female	No	Exposure B	60	60	60	500	High Schoo	Middle Sch	Orthodox	Georgian	Village
Low Risk	1	20	Male	No	Exposure B	13	13	10	4000	High Schoo	High Schoo	Orthodox	Georgian	City/Town
Low Risk	1	22	Female	No	Exposure B	60	60	60	500	High Schoo	Middle Sch	Orthodox	Georgian	Village
Low Risk	0	20	Male	No	Exposure B	50	50	50	15002	Bachelor	Bachelor	Orthodox	Georgian	City/Town
Low Risk	0	29	Male	No	Exposure B	15	90	190		Bachelor	Bachelor	Orthodox	Georgian	City/Town
Low Risk	0	20	Male	No	Not Expose	60	100	200	1500	High Schoo	High Schoo	Orthodox	Georgian	City/Town
Low Risk	1	20	Female	No	Not Expose	0	0	0	8000	Bachelor	Bachelor	Other	Georgian	City/Town
Low Risk	1	25	Male	No	Exposure B	30	30	30	2500	Bachelor	High Schoo	Orthodox	Georgian	Village
Low Risk	1	26	Female	No	Exposure B	90	30	50	3000	Bachelor	Bachelor	Orthodox	Georgian	Village
Low Risk	0	29	Male	No	Exposure B	30	30	30	1500	Bachelor	Bachelor	Orthodox	Georgian	City/Town
Low Risk	2	23	Male	Yes	Exposure B	360	180	180	3000	Bachelor	Master or I	Orthodox	Georgian	City/Town
Low Risk	0	24	Female	No	Exposure B	20	20	15	2000	Master or I	Bachelor	Orthodox	Georgian	Village
Low Risk	0	23	Female	No	Not Expose	15	90	120	500	Bachelor	Bachelor	Orthodox	Georgian	Village
Low Risk	1	22	Male	No	Exposure B	30	90	40	1500	Bachelor	Bachelor	Orthodox	Georgian	City/Town
Low Risk	1	18	Male	No	Exposure B	90	180	180	1500	Master or I	Bachelor	Orthodox	Georgian	Village
Low Risk	0	18	Male	No	Not Expose	0	30	30	1500	Master or I	Bachelor	Orthodox	Georgian	City/Town

Low Risk	0	25	Female	Yes	Exposure B	90	90	90	3000	Bachelor	High Schoo	Orthodox	Georgian	City/Town
Low Risk	2	25	Female	No	Exposure B	180	30	30	2000	Bachelor	Bachelor	Orthodox	Georgian	City/Town
Low Risk	1	24	Male	No	Exposure B	30	30	20	2000	Bachelor	High Schoo	Orthodox	Armenian	City/Town
Low Risk	2	29	Female	No	Exposure B	60	60	60	1500	High Schoo	High Schoo	Orthodox	Georgian	Mountainous Region
Low Risk	0	23	Female	No	Not Expose	20	20	10	10000	Master or I	Master or I	Orthodox	Georgian	City/Town
Low Risk														
Low Risk	1	25	Male	Yes	Exposure B	90	90	90	3000	High Schoo	Bachelor	Orthodox	Georgian	City/Town
Low Risk	0	23	Female	No	Exposure B	30	30	0	3000	Bachelor	Bachelor	Orthodox	Georgian	Emigrant (West)
Low Risk	2	26	Female	No	Exposure B	30	90	150	1500	High Schoo	High Schoo	Orthodox	Georgian	Village
Medium Ri	5	18	Male	No	Not Expose	0	0	0	14000	Bachelor	Bachelor	Orthodox	Other	Emigrant (West)
Medium Ri	4	26	Male	No	Exposure B	30	30	30		Bachelor	High Schoo	Orthodox	Georgian	Village
Low Risk	1	17	Male	No	Not Expose	0	0	0	15000	Master or I	Master or I	Orthodox	Georgian	Village
Low Risk	2	26	Male	No	Exposure B	30	30	30	1500	Master or I	High Schoo	Orthodox	Georgian	Village
Low Risk	1	24	Male	No	Not Expose	60	60	60	1500	High Schoo	High Schoo	Orthodox	Georgian	City/Town
High Risk	16	19	Male	No	Not Expose	30	30	30	1500	Bachelor	High Schoo	Orthodox	Georgian	Village
Low Risk	0	28	Female	No	Exposure B	60	60	60	3000	Bachelor	Bachelor	Orthodox	Georgian	City/Town
High Risk	8	25	Male	Yes	Exposure B	90	90	90	3000	High Schoo	High Schoo	Orthodox	Georgian	City/Town
Low Risk	1	24	Female	Yes	Exposure B	90	90	90	1500	High Schoo	High Schoo	Orthodox	Georgian	City/Town
Low Risk	0	27	Male	Yes	Exposure B	120				Master or I	Bachelor	Orthodox	Georgian	Village
Low Risk	0	27	Male	Yes	Exposure B	90	30	30	3000	Bachelor	Bachelor	Orthodox	Georgian	Emigrant (West)
Medium Ri	3	18	Male	No	Not Expose	90	90	180	1500	High Schoo	High Schoo	Orthodox	Georgian	City/Town
Medium Ri	3	25	Male	No	Exposure B	90	90	90		Bachelor	Master or I	Orthodox	Georgian	City/Town
Low Risk	1	19	Male	No	Exposure B	15	15	15	1500	Bachelor	Master or I	Orthodox	Georgian	City/Town
Low Risk	0	24	Male	No	Exposure B	30	20	10	2000	High Schoo	High Schoo	Orthodox	Georgian	City/Town
Low Risk	0	18	Male	No	Exposure B	30	30	30	2000	High Schoo	High Schoo	Orthodox	Georgian	Village
Medium Ri	5	19	Male	Yes	Exposure B	360	360	360	1500	High Schoo	High Schoo	Orthodox	Georgian	City/Town
Low Risk	0	19	Female	Yes	Exposure B	90	90	90	3000	Master or I	Bachelor	Orthodox	Georgian	City/Town
Medium Ri	6	20	Male	Yes	Exposure B	30	90	360	3000	Middle Sch	Middle Sch	Orthodox	Georgian	Emigrant (West)
Medium Ri	4	17	Male	Yes	Exposure B	180	360	360	2500	Bachelor	Bachelor	Orthodox	Georgian	City/Town
Low Risk	0	19	Male	No	Not Expose	0	0	0	2000	Bachelor	Master or I	Orthodox	Georgian	City/Town
Medium Ri	5	21	Male	Yes	Exposure B	90	90	360	3000	High Schoo	High Schoo	Orthodox	Georgian	City/Town
Medium Ri	7	20	Male	Yes	Exposure B	90	180	360	3000	Middle Sch	Middle Sch	Orthodox	Georgian	Emigrant (West)
Medium Ri	5	25	Male	Yes	Exposure B	90	30	30	2000	High Schoo	High Schoo	Orthodox	Georgian	City/Town
Low Risk	0	26	Male	No	Not Expose	0	0	0	0	Master or I	Bachelor	Orthodox	Georgian	Emigrant (West)
Low Risk	2	24	Male	No	Not Expose	0	0	0	3000	Bachelor	Master or I	Other	Georgian	City/Town
Low Risk														
Low Risk	0	18	Female	Yes	Exposure B	90	360	90	3000	Master or I	Middle Sch	Orthodox	Georgian	City/Town
Medium Ri	3	24	Male	Yes	Exposure B	10	20	20	2000	High Schoo	Master or I	Orthodox	Georgian	City/Town
Low Risk	0	23	Male	Yes	Exposure B	30	30	90	1500	Bachelor	Bachelor	Orthodox	Georgian	City/Town
Low Risk	2	21	Female	Yes	Exposure B	20	90	50	2000	Bachelor	Bachelor	Orthodox	Georgian	City/Town
Low Risk	1	19	Male	No	Not Expose	0	0	0	3000	Bachelor	High Schoo	Orthodox	Georgian	City/Town
Low Risk	0	18	Female	No	Not Expose	10	10	10	500	High Schoo	High Schoo	Orthodox	Georgian	Mountainous Region
Low Risk	0	24	Male	Yes	Exposure B	90	90	90	5000	Bachelor	PhD	Orthodox	Georgian	Village
Low Risk	0	19	Male	Yes	Exposure B	0	360	360	2000	Master or I	Bachelor	Orthodox	Georgian	City/Town

Low Risk	1	18	Male	No	Exposure B	30	30	30	500	Bachelor	Middle Sch	Orthodox	Georgian	City/Town
Low Risk	0	20	Male	No	Exposure B	5	5	2	5000	Master or I	Bachelor	Orthodox	Georgian	City/Town
Low Risk	1	22	Male	No	Not Expose	0	5	30	10000	Bachelor	Master or I	Orthodox	Georgian	City/Town
Low Risk	2	24	Male	Yes	Exposure B	10	20	20	2000	High Schoo	Bachelor	Orthodox	Georgian	City/Town
Low Risk	1	26	Female	No	Exposure B	30	30	30	3000	Middle Sch	High Schoo	Orthodox	Georgian	City/Town
Low Risk	0	20	Female	Yes	Exposure B	180	180	360	3000	Master or I	Master or I	Orthodox	Mixed	City/Town
High Risk	8	30	Male	No	Exposure B	90	90	90	4000	PhD	Master or I	Orthodox	Georgian	City/Town
Low Risk	0	20	Female	No	Exposure Between 12 and 18 Months					Master or I	Bachelor	Orthodox	Georgian	City/Town
Medium Ri	3	17	Female	Yes	Exposure B	30	360	30	500	Bachelor	Middle Sch	Orthodox	Georgian	City/Town
Low Risk	0	20	Female	No	Exposure B	10	10	10	1000	Bachelor	Master or I	Orthodox	Georgian	City/Town
Low Risk	0	29	Male	Yes	Exposure B	30	180	180	2500	Bachelor	High Schoo	Orthodox	Georgian	City/Town
Medium Ri	4	27	Female	Yes	Exposure B	360	360	360	600	High Schoo	Middle Sch	Other	Other	City/Town
Low Risk	0	23	Male	No	Exposure B	30	30	10	800	Bachelor	High Schoo	Orthodox	Georgian	Village
Low Risk	2	18	Male	Yes	Exposure B	180	180	300	3000	High Schoo	High Schoo	Muslim	Georgian	Village
Medium Ri	3	24	Male	Yes	Exposure B	90	360	360	1500	Bachelor	Bachelor	Orthodox	Georgian	Village
Low Risk	1	20	Male	No	Exposure B	60	30	30	1500	Master or I	High Schoo	Orthodox	Georgian	City/Town
Low Risk	1	27	Male	Yes	Exposure B	30	30	30	1000	Bachelor	High Schoo	Orthodox	Georgian	Village
Low Risk														
Low Risk	0	22	Male	No	Exposure B	60	60	30	5000	Bachelor	Bachelor	Orthodox	Georgian	City/Town
Low Risk	0	19	Female	Yes	Exposure B	10	15	10	900	High Schoo	High Schoo	Orthodox	Georgian	City/Town
Low Risk	2	21	Male	No	Exposure B	30	40	40	5000	Bachelor	Master or I	Orthodox	Georgian	Emigrant (West)
Medium Ri	3	23	Male	Yes	Exposure B	30	60	60	3000	Bachelor	Bachelor	Orthodox	Georgian	Village
High Risk	8	18	Male	Yes	Exposure B	240	240	300	3000	High Schoo	High Schoo	Muslim	Georgian	Village
Low Risk	1	20	Female	No	Exposure B	30	30	30	1500	Bachelor	High Schoo	Orthodox	Georgian	Village
Low Risk	0	18	Male	Yes	Exposure B	30	30	30	2500	Master or I	High Schoo	Orthodox	Georgian	City/Town
Low Risk	2	22	Female	Yes	Exposure B	180	180	180	300	High Schoo	High Schoo	Orthodox	Georgian	Village
Low Risk	0	26	Female	Yes	Exposure B	180	180	180	3000	Bachelor	Bachelor	Orthodox	Georgian	City/Town
Medium Ri	3	30	Female	No	Exposure B	30	30	30	1500	High Schoo	Middle Sch	Orthodox	Georgian	Village
Low Risk	1	18	Male	Yes	Exposure B	0	40	30	3000	Bachelor	High Schoo	Orthodox	Georgian	City/Town
Low Risk	2	23	Male	No	Exposure B	15	15	20	2000	Bachelor	Bachelor	Orthodox	Georgian	City/Town
Low Risk	2	17	Female	Yes	Exposure B	30	30	30	3000	High Schoo	High Schoo	Orthodox	Georgian	City/Town
Low Risk	0	17	Female	No	Exposure B	18	105	105	1500	Bachelor	Bachelor	Orthodox	Georgian	Village
Low Risk	2	18	Female	Yes	Exposure B	180	360	180	1500	Master or I	Master or I	Orthodox	Georgian	Emigrant (West)
Medium Ri	3	23	Male	Yes	Exposure B	20	120	240	1000	Bachelor	High Schoo	Orthodox	Georgian	Village
Medium Ri	7	19	Male	Yes	Exposure B	90	90	180	2500	High Schoo	High Schoo	Orthodox	Georgian	City/Town
Low Risk	0	26	Male	No	Exposure B	90	300	360	3500	Master or I	Bachelor	Orthodox	Georgian	City/Town
Low Risk	2	25	Male	No	Not Expose	30	30	30	2000	Middle Sch	Middle Sch	Orthodox	Georgian	City/Town
Low Risk	0	24	Female	Yes	Exposure B	15	15	30	4000	Bachelor	Bachelor	Orthodox	Georgian	City/Town
Low Risk	0	24	Female	Yes	Exposure B	30	30	30	5000	Master or I	Master or I	Orthodox	Georgian	City/Town
Low Risk	0	21	Female	Yes	Exposure B	20	30	30	3000	Master or I	Master or I	Orthodox	Georgian	City/Town
Low Risk	0	20	Female	Yes	Exposure B	30	25	30	2000	High Schoo	Bachelor	Orthodox	Georgian	City/Town
Medium Ri	5	30	Male	Yes	Exposure B	180	360	180	3000	High Schoo	High Schoo	Orthodox	Georgian	Village
Low Risk	0	28	Male	Yes	Exposure B	90	90	90	1500	High Schoo	High Schoo	Orthodox	Georgian	City/Town
Low Risk	1	23	Male	No	Exposure B	90	90	90	3000	Bachelor	High Schoo	Orthodox	Georgian	City/Town

Low Risk	0	22	Male	No	Exposure B	30	30	30	3000	Master or	Master or	Orthodox	Georgian	Emigrant (West)
Low Risk	0	24	Male	No	Exposure B	20	20	20	2000	Master or	Master or	Orthodox	Georgian	City/Town
Low Risk	1	19	Female	Yes	Exposure B	20	20	20	1500	Bachelor	High Schoo	Orthodox	Mixed	Emigrant (West)
Medium Ri	7	19	Male	Yes	Exposure B	45	20	90	3000	Bachelor	High Schoo	Orthodox	Georgian	City/Town
Low Risk	1	25	Female	Yes	Exposure B	30	23	15	3000	Bachelor	Master or	Orthodox	Georgian	City/Town
Medium Ri	7	24	Male	Yes	Exposure B	90	180	180	1500	High Schoo	High Schoo	Orthodox	Georgian	Village
High Risk	11	26	Female	Yes	Exposure B	90	90	90	4000	Bachelor	High Schoo	Orthodox	Georgian	City/Town
High Risk	8	30	Male	Yes	Exposure B	60	180	360	2000	Bachelor	Bachelor	Orthodox	Georgian	Village
Low Risk	0	25	Female	No	Exposure B	0	30	30	1500	High Schoo	High Schoo	Orthodox	Georgian	City/Town
Low Risk	2	17	Male	No	Not Expose	40	40	40	0	High Schoo	Bachelor	Orthodox	Georgian	City/Town
Low Risk	0	19	Female	Yes	Exposure B	90	90	180	3000	Bachelor	Bachelor	Orthodox	Georgian	City/Town
Low Risk	1	18	Male	No	Exposure B	0	60	90	2500	Master or	Master or	Orthodox	Georgian	City/Town
Low Risk	0	18	Male	No	Exposure B	90	90	90	500	Master or	PhD	Other	Other	City/Town
Medium Ri	5	21	Male	No	Not Expose	30	90	180	1500	High Schoo	High Schoo	Protestant	Azerbaijan	City/Town
Medium Ri	3	30	Male	Yes	Exposure B	180	90	180	1500	Bachelor	High Schoo	Orthodox	Georgian	Emigrant (West)
High Risk	13	25	Female	Yes	Exposure B	360	30	30	6000	Bachelor	Bachelor	Orthodox	Mixed	City/Town
Low Risk	0	17	Male	No	Not Expose	0	0	0	5000	Master or	Master or	Orthodox	Georgian	City/Town
Low Risk	1	18	Female	No	Not Expose	0	0	0	1500	Bachelor	Bachelor	Orthodox	Georgian	City/Town
Low Risk	0	25	Male	Yes	Exposure B	80	90	90	7000	Master or	Bachelor	Orthodox	Georgian	City/Town
Low Risk	0	20	Male	No	Exposure B	30	30	30	500	Middle Sch	Middle Sch	Other	Georgian	Village
Low Risk	0	25	Male	Yes	Exposure B	90	90	90	7000	Master or	Bachelor	Orthodox	Georgian	City/Town
Medium Ri	3	24	Male	Yes	Exposure B	30	30	30	5000	Bachelor	High Schoo	Orthodox	Georgian	City/Town
Medium Ri	3	18	Female	Yes	Exposure B	90	90	180	500	Bachelor	PhD	Orthodox	Georgian	City/Town
Low Risk	1	24	Male	No	Exposure B	30	90	180	3000	Bachelor	Master or	Orthodox	Mixed	Emigrant (West)
Low Risk														
Medium Ri	6	24	Female	Yes	Exposure B	90	90	180		High Schoo	High Schoo	Orthodox	Georgian	Emigrant (West)
Low Risk	1	24	Male	No	Exposure B	90	90	180	1500	Master or	Master or	Orthodox	Georgian	City/Town
Low Risk	0	29	Female	Yes	Exposure B	60	90	60	3500	High Schoo	High Schoo	Orthodox	Georgian	City/Town
Medium Ri	3	24	Male	Yes	Exposure B	30	30	30	3000	Bachelor	Bachelor	Orthodox	Georgian	City/Town
Low Risk	0	20	Male	No	Exposure B	30	30	30	1500	Middle Sch	Middle Sch	Other	Georgian	Village
Low Risk	1	23	Male	Yes	Exposure B	90	90	90	1000	High Schoo	High Schoo	Orthodox	Georgian	Emigrant (West)
Low Risk	1	20	Female	No	Exposure B	30	300	400	3000	Bachelor	High Schoo	Orthodox	Georgian	City/Town
Low Risk	0	24	Male	Yes	Exposure B	100	360	360	200	High Schoo	High Schoo	Muslim	Other	Mountainous Region
Low Risk	1	25	Female	Yes	Exposure B	180	180	180	500	High Schoo	Bachelor	Orthodox	Georgian	Village
Low Risk	1	18	Male	Yes	Exposure B	10	10	10	2000	Bachelor	Bachelor	Orthodox	Georgian	City/Town
High Risk	8	25	Male	Yes	Exposure B	180	30	90	500	Bachelor	High Schoo	Orthodox	Georgian	Village
Low Risk	0	24	Male	Yes	Exposure B	90	90	180	3000	Bachelor	Master or	Orthodox	Georgian	City/Town
Low Risk	0	22	Female	Yes	Exposure B	30	30	30	3000	Bachelor	Bachelor	Orthodox	Georgian	City/Town
Low Risk	2	19	Male	Yes	Exposure B	180	180	180	3000	Bachelor	Bachelor	Orthodox	Georgian	City/Town
Low Risk	0	18	Female	No	Not Expose	0	0	0	0	Master or	Bachelor	Orthodox	Georgian	City/Town
Medium Ri	4	24	Male	Yes	Exposure B	420	90	180	1000	High Schoo	High Schoo	Orthodox	Georgian	City/Town
Low Risk	1	27	Male	No	Not Expose	0	0	0	2000	Bachelor	Master or	Orthodox	Georgian	City/Town
Low Risk	0	27	Male	Yes	Exposure B	30	30	30	2000	Bachelor	Bachelor	Orthodox	Georgian	Village
Low Risk	1	29	Female	No	Not Expose	0	0	0	1500	High Schoo	Middle Sch	Orthodox	Georgian	City/Town

Low Risk	1	24	Female	Yes	Exposure B	30	30	30	2000	High Schoo	High Schoo	Orthodox	Georgian	Village
Low Risk	1	20	Male	No	Not Expose	0	0	0	0	Bachelor	High Schoo	Orthodox	Georgian	City/Town
Medium Ri	4	20	Male	Yes	Exposure B	180	180	180	3000	Bachelor	High Schoo	Orthodox	Georgian	City/Town
Low Risk	0	28	Male	No	Exposure B	90	90	30	2500	Bachelor	Bachelor	Orthodox	Georgian	Village
Low Risk	1	24	Male	No	Exposure B	90	90	30	1200	High Schoo	High Schoo	Orthodox	Georgian	City/Town
Low Risk	0	24	Female	No	Exposure B	15	10	5	2600	High Schoo	Bachelor	Orthodox	Georgian	City/Town
Low Risk	1	17	Male	Yes	Exposure B	30	30	30	1500	Bachelor	Bachelor	Orthodox	Georgian	City/Town
Low Risk	0	17	Female	No	Not Expose	5	10	10	2000	High Schoo	High Schoo	Orthodox	Georgian	City/Town
Medium Ri	3	30	Male	Yes	Exposure B	180	90	90	3000	Master or I	Bachelor	Orthodox	Georgian	City/Town
Low Risk	0	20	Male	Yes	Exposure B	90	60	60	0	Master or I	Bachelor	Orthodox	Georgian	City/Town
Medium Ri	7	24	Male	Yes	Exposure B	30	30	30	3000	High Schoo	High Schoo	Orthodox	Georgian	City/Town
Low Risk	0	28	Male	No	Exposure B	30	30	40	960	High Schoo	Middle Sch	Orthodox	Georgian	City/Town
Medium Ri	5	22	Male	No	Exposure B	180	180	0	3000	Bachelor	Bachelor	Orthodox	Georgian	Emigrant (West)
Low Risk	0	26	Male	Yes	Exposure B	360	180	180	2000	Bachelor	PhD	Orthodox	Georgian	City/Town
Low Risk	0	25	Male	No	Not Expose	10	60	60	1500	Bachelor	Bachelor	Orthodox	Georgian	City/Town
Medium Ri	5	30	Female	Yes	Exposure B	180	180	180	200	High Schoo	Master or I	Orthodox	Georgian	City/Town
Low Risk	0	24	Male	No	Not Expose	30	30	30	500	High Schoo	High Schoo	Orthodox	Georgian	Emigrant (West)
High Risk	11	30	Male	Yes	Exposure B	30	30	30	150	Bachelor	Bachelor	Orthodox	Georgian	City/Town
Low Risk	1	25	Female	Yes	Exposure B	90	90	90	3000	High Schoo	Bachelor	Orthodox	Mixed	City/Town
Medium Ri	6	30	Male	No	Exposure B	60	60	60	2000	Bachelor	Master or I	Orthodox	Georgian	City/Town
Medium Ri	4	24	Female	Yes	Exposure B	30	90	90	1000	High Schoo	High Schoo	Orthodox	Georgian	Village
Medium Ri	4	20	Male	Yes	Exposure B	180	240	240	3000	Bachelor	Master or I	Orthodox	Georgian	City/Town
Low Risk	0	25	Female	No	Not Expose	30	360	360	1500	High Schoo	High Schoo	Orthodox	Georgian	Mountainous Region
Low Risk	2	24	Male	No	Not Expose	0	0	0	1500	High Schoo	High Schoo	Orthodox	Georgian	City/Town
Medium Ri	4	24	Female	Yes	Exposure B	30	90	90	3000	High Schoo	High Schoo	Orthodox	Georgian	Village
Low Risk	2	24	Female	Yes	Exposure B	90	90	180	3000	Bachelor	Bachelor	Orthodox	Georgian	City/Town
Low Risk	0	24	Male	No	Exposure B	90	90	360	3000	Bachelor	Bachelor	Orthodox	Russian	Emigrant (Asia/Russia/Africa)
Medium Ri	7	30	Female	No	Exposure B	40	30	15	1500	High Schoo	High Schoo	Muslim	Georgian	City/Town
Medium Ri	6	23	Male	Yes	Exposure B	15	15	15		Middle Sch	High Schoo	Atheist	Georgian	City/Town
Low Risk	2	29	Male	Yes	Exposure B	90	90	90	500	High Schoo	High Schoo	Orthodox	Georgian	City/Town
Low Risk	0	24	Female	No	Exposure B	90	90	30	1000	Bachelor	Master or I	Orthodox	Ukrainian	City/Town
Medium Ri	5	25	Male	Yes	Exposure B	90	90	30	2500	High Schoo	High Schoo	Orthodox	Georgian	Mountainous Region
Low Risk	0	18	Male	Yes	Exposure B	30	40	40	3000	High Schoo	Bachelor	Muslim	Azerbaijan	City/Town
Low Risk	0	17	Male	Yes	Exposure B	120	60	30	3000	Master or I	Master or I	Orthodox	Georgian	City/Town
Low Risk	1	24	Female	Yes	Exposure B	90	180	180	4000	Bachelor	Master or I	Orthodox	Georgian	City/Town
Low Risk	2	25	Female	Yes	Exposure B	30	180	20	3000	Middle Sch	High Schoo	Orthodox	Georgian	Emigrant (West)
Medium Ri	5	24	Male	Yes	Exposure B	180	180	90	1300	Bachelor	High Schoo	Orthodox	Georgian	Village
Low Risk	0	29	Male	No	Exposure B	30	0	0	2000	Bachelor	Bachelor	Orthodox	Georgian	City/Town
High Risk	15	30	Male	No	Exposure B	30	30	30	1500	Bachelor	Bachelor	Orthodox	Georgian	City/Town
Medium Ri	4	18	Male	Yes	Exposure B	30	30	30	3000	High Schoo	High Schoo	Orthodox	Georgian	Emigrant (Asia/Russia/Africa)
Low Risk	0	18	Female	Yes	Exposure B	10	10	10	10000	Bachelor	Master or I	Orthodox	Other	Emigrant (West)
Low Risk	1	22	Female	No	Not Expose	0	0	0	1000	Bachelor	Bachelor	Orthodox	Georgian	City/Town
Low Risk	0	21	Male	No	Not Expose	3	0	2	2000	Master or I	High Schoo	Orthodox	Georgian	City/Town
Low Risk	0	24	Male	No	Exposure B	30	30	30	3000	Master or I	PhD	Orthodox	Georgian	City/Town

Low Risk	1	19	Male	No	Not Expose	0	30	30	3000	Master or I	Bachelor	Orthodox	Georgian	City/Town
Low Risk	1	24	Male	Yes	Exposure B	15	15	15	3000	Bachelor	Middle Sch	Orthodox	Georgian	Village
Low Risk	2	18	Male	No	Not Expose	50	50	50	7000	Bachelor	Master or I	Orthodox	Georgian	City/Town
Low Risk	0	20	Male	No	Not Expose	0	0	0	3000	High Schoo	High Schoo	Orthodox	Georgian	Emigrant (West)
Low Risk	0	28	Female	No	Exposure B	15	10	7	3500	Bachelor	Bachelor	Orthodox	Georgian	City/Town
Low Risk	1	18	Male	No	Exposure B	5	30	30	4000	Master or I	Master or I	Orthodox	Georgian	City/Town
Low Risk	0	19	Male	Yes	Exposure B	20	30	30	800	Bachelor	High Schoo	Orthodox	Georgian	City/Town
Low Risk	0	29	Female	No	Exposure B	30	360	360	3000	Bachelor	Master or I	Orthodox	Georgian	Village
Low Risk	0	20	Male	No	Not Expose	0	0	0	3000	High Schoo	High Schoo	Orthodox	Georgian	Emigrant (West)
Low Risk	1	18	Female	Yes	Exposure B	30	30	30	3000	Bachelor	Bachelor	Orthodox	Georgian	City/Town
Low Risk	1	20	Male	Yes	Exposure B	120	120	120	2500	High Schoo	High Schoo	Orthodox	Ukrainian	City/Town
Low Risk	0	20	Female	No	Exposure B	18	17	17	5000	Master or I	Bachelor	Orthodox	Georgian	City/Town
Low Risk	0	29	Female	No	Exposure B	100	100	80	5000	Bachelor	Master or I	Orthodox	Georgian	City/Town
Low Risk	0	24	Female	No	Not Expose	10	30	90	1500	Middle Sch	High Schoo	Orthodox	Mixed	City/Town
Low Risk	0	22	Male	No	Exposure B	30	30	30	2500	Bachelor	Bachelor	Orthodox	Georgian	City/Town
Low Risk	0	27	Female	Yes	Exposure B	60	180	360	3000	Bachelor	Bachelor	Orthodox	Georgian	City/Town
Low Risk	1	19	Male	No	Not Expose	0	0	0	600	Bachelor	Bachelor	Orthodox	Georgian	Village
Low Risk	2	19	Male	Yes	Exposure B	90	90	90	1000	High Schoo	High Schoo	Orthodox	Georgian	City/Town
Medium Ri	3	25	Male	Yes	Exposure B	20	30	90	2000	High Schoo	Master or I	Other	Mixed	City/Town
Low Risk	2	28	Male	Yes	Exposure B	180	360	360	1000	Bachelor	Bachelor	Orthodox	Georgian	City/Town
Low Risk	0	25	Male	Yes	Exposure B	35	180	360	1500	Bachelor	Bachelor	Orthodox	Georgian	City/Town
Low Risk	2	29	Male	No	Not Expose	30	360	360	4000	Master or I	High Schoo	Orthodox	Georgian	Village
Low Risk	1	27	Female	No	Exposure B	5	5	5	3000	Bachelor	Bachelor	Orthodox	Georgian	City/Town
Low Risk	0	21	Male	No	Exposure B	30	360	500	2000	Bachelor	Bachelor	Orthodox	Georgian	City/Town
Medium Ri	6	30	Female	No	Not Expose	400	400	400	8000	High Schoo	Middle Sch	Muslim	Georgian	City/Town
High Risk	8	25	Male	Yes	Exposure B	60	120	150	2000	High Schoo	Master or I	Other	Mixed	City/Town
Medium Ri	4	24	Female	Yes	Exposure B	15	90	90	5000	Master or I	Master or I	Orthodox	Georgian	City/Town
Low Risk	1	24	Female	No	Exposure B	30	30	30	1500	Bachelor	Bachelor	Orthodox	Georgian	City/Town
Medium Ri	3	20	Female	No	Exposure B	30	90	90	3000	High Schoo	Bachelor	Orthodox	Georgian	Mountainous Region
Low Risk	0	17	Male	Yes	Exposure B	30	90	180	3000	Bachelor	Bachelor	Orthodox	Georgian	Mountainous Region
Low Risk	1	18	Male	Yes	Exposure B	15	0	0	3500	High Schoo	High Schoo	Orthodox	Georgian	City/Town
Medium Ri	7	23	Female	No	Exposure B	10	120	360	1000	High Schoo	High Schoo	Orthodox	Georgian	City/Town
Low Risk	0	24	Male	Yes	Exposure B	30	30	30	500	Bachelor	Bachelor	Orthodox	Georgian	City/Town
Medium Ri	6	30	Female	Yes	Exposure B	60	100	100	1500	Bachelor	Bachelor	Orthodox	Georgian	Emigrant (West)
Low Risk	0	28	Female	No	Exposure B	30	180	90	100	Bachelor	High Schoo	Orthodox	Georgian	City/Town
Low Risk	0	22	Male	No	Exposure B	30	180	180	1500	High Schoo	High Schoo	Muslim	Georgian	Village
Low Risk	1	20	Female	No	Not Expose	15	15	15	3000	Master or Equivalent		Orthodox	Georgian	City/Town
Low Risk	0	17	Male	Yes	Exposure B	40	20	20	4000	Master or I	High Schoo	Orthodox	Mixed	Emigrant (West)
Low Risk														
Low Risk	0	24	Male	No	Exposure B	60	60	60	1500	Bachelor	High Schoo	Orthodox	Georgian	City/Town
Low Risk	0	18	Male	No	Exposure B	90	30	30	1500	Bachelor	Bachelor	Orthodox	Georgian	City/Town
Low Risk	1	21	Male	No	Exposure B	30	20	20	5000	Bachelor	Bachelor	Orthodox	Georgian	City/Town
Low Risk	0	20	Male	No	Exposure B	30	30	30	3000	Master or I	Bachelor	Orthodox	Georgian	City/Town
Low Risk														

Low Risk	0	17	Female	No	Exposure B	30	30	30	2000	Bachelor	Bachelor	Orthodox	Georgian	City/Town
Low Risk	0	28	Male	Yes	Exposure B	30	30	30	1500	Bachelor	Bachelor	Orthodox	Georgian	City/Town
Low Risk	0	22	Male	Yes	Exposure B	30	30	30	3000	High Schoo	High Schoo	Orthodox	Georgian	City/Town
High Risk	10	21	Male	Yes	Exposure B	180	30	180	3000	Bachelor	Bachelor	Orthodox	Georgian	Village
Low Risk	0	18	Female	No	Not Expose	0	0	0	2000	Master or I	Master or I	Orthodox	Georgian	Village
Low Risk	2	28	Female	Yes	Exposure B	30	30	400	3000	Bachelor	Bachelor	Orthodox	Georgian	City/Town
Low Risk	0	17	Male	No	Exposure B	30	30	30	2000	Bachelor	Bachelor	Orthodox	Georgian	City/Town
Low Risk	0	24	Female	No	Exposure B	90	60	60	3500	Bachelor	Bachelor	Muslim	Other	City/Town
Low Risk	0	24	Male	Yes	Exposure B	30	40	30	4000	Bachelor	High Schoo	Orthodox	Georgian	Village
High Risk	8	30	Male	Yes	Exposure B	90	180	360	1500	High Schoo	High Schoo	Orthodox	Georgian	Emigrant (West)
High Risk	9	22	Female	Yes	Exposure B	60	360	540	10000	Master or I	Bachelor	Orthodox	Georgian	City/Town
Medium Ri	3	18	Male	Yes	Exposure B	30	38			Bachelor	Bachelor	Orthodox	Georgian	City/Town
Low Risk	1	19	Male	Yes	Exposure B	90	30	30	3000	Bachelor	High Schoo	Orthodox	Georgian	City/Town
Low Risk	0	17	Male	No	Exposure B	30	30	30	2000	Bachelor	Bachelor	Orthodox	Georgian	City/Town
Low Risk	0	18	Female	Yes	Exposure B	30	30	30	1500	High Schoo	High Schoo	Orthodox	Georgian	City/Town
Medium Ri	6	27	Female	No	Exposure B	30	90	90	1500	High Schoo	High Schoo	Orthodox	Georgian	City/Town
Medium Ri	4	30	Male	No	Exposure B	180	360	90	1500	Bachelor	High Schoo	Orthodox	Georgian	Village
Low Risk	0	18	Male	No	Not Expose	30	30	30	5000	Bachelor	Bachelor	Orthodox	Georgian	City/Town
Low Risk	0	22	Female	Yes	Exposure B	90	20	30		Bachelor	Bachelor	Orthodox	Georgian	City/Town
Low Risk	0	20	Male	Yes	Exposure B	30	30	30	3000	Bachelor	Bachelor	Orthodox	Georgian	City/Town
Low Risk	0	21	Female	No	Exposure B	15	15	10	7000	Bachelor	Bachelor	Orthodox	Georgian	City/Town
Low Risk	0	24	Male	No	Not Expose	0	0	0	2000	High Schoo	High Schoo	Orthodox	Georgian	City/Town
Low Risk	1	19	Female	Yes	Exposure B	90	90	90		Bachelor	Bachelor	Orthodox	Other	Emigrant (Asia/Russia/Africa)
Low Risk	0	19	Male	No	Not Exposed Yet				2000	Master or I	Master or I	Orthodox	Georgian	City/Town
Low Risk	0	24	Male	No	Not Expose	5	40	50	10000	PhD	Master or I	Orthodox	Georgian	City/Town
Low Risk	0	23	Male	No	Exposure B	130	90	180	1500	Bachelor	Bachelor	Orthodox	Georgian	Village
Low Risk	0	20	Male	No	Exposure B	15	15	15	500	Bachelor	Bachelor	Orthodox	Georgian	City/Town
Medium Ri	7	19	Male	Yes	Exposure B	30	10	10	100	Bachelor	High Schoo	Orthodox	Georgian	City/Town
Low Risk	0	18	Male	No	Not Expose	30	60	30	2000	Bachelor	Bachelor	Orthodox	Georgian	City/Town
Low Risk	0	24	Male	No	Not Expose	30	180	180	500	High Schoo	High Schoo	Muslim	Georgian	Village
Low Risk	0	20	Male	Yes	Exposure B	30	30	30	10000	High Schoo	High Schoo	Orthodox	Georgian	City/Town
High Risk	13	29	Female	No	Exposure B	30	150	30	2000	High Schoo	High Schoo	Orthodox	Georgian	City/Town
Low Risk	0	27	Male	Yes	Exposure B	15	15	15	3000	Bachelor	High Schoo	Orthodox	Georgian	City/Town
Low Risk	1	24	Male	No	Not Expose	60	60	60	3000	Bachelor	High Schoo	Orthodox	Georgian	City/Town
Low Risk	2	20	Male	Yes	Exposure B	30	30	30	0	Bachelor	Bachelor	Orthodox	Georgian	Emigrant (Asia/Russia/Africa)
Low Risk	1	29	Male	Yes	Exposure B	3	360	370	600	High Schoo	High Schoo	Orthodox	Georgian	City/Town
Low Risk	1	19	Female	No	Exposure B	10	20	20	1500	Bachelor	High Schoo	Orthodox	Georgian	City/Town
Low Risk	1	24	Male	Yes	Exposure B	150	120	150	4500	Bachelor	Bachelor	Orthodox	Georgian	City/Town
Low Risk	2	21	Male	No	Not Expose	0	0	0	6000	Bachelor	Bachelor	Orthodox	Georgian	Village
Low Risk	0	24	Male	Yes	Exposure B	90	90	90	4500	Bachelor	Bachelor	Orthodox	Georgian	City/Town
Medium Ri	7	24	Female	No	Exposure B	30	90	90	1500	Bachelor	Middle Sch	Orthodox	Georgian	Village
Medium Ri	7	30	Male	Yes	Exposure B	180	180	189	1000	Master or I	Master or I	Orthodox	Georgian	City/Town
Low Risk	0	20	Female	No	Exposure B	20	20	10	1000	Master or I	Bachelor	Orthodox	Georgian	Village
Low Risk	0	20	Male	No	Not Expose	0	0	0	4000	Bachelor	Bachelor	Orthodox	Georgian	City/Town

Low Risk	1	18	Female	No	Exposure B	30	20	0	4000	High Schoo	High Schoo	Orthodox	Georgian	City/Town
Low Risk	1	29	Female	No	Exposure B	30	500		3000	Master or I	Master or I	Orthodox	Georgian	City/Town
High Risk	8	22	Male	Yes	Exposure B	360	360	360	1500	Master or I	Bachelor	Orthodox	Georgian	City/Town
Low Risk	1	26	Male	Yes	Exposure B	0	0	90	3000	Master or I	Bachelor	Orthodox	Georgian	City/Town
Low Risk	0	24	Male	No	Not Expose	0	30	15	4500	Bachelor	High Schoo	Orthodox	Georgian	City/Town
Low Risk	1	23	Female	Yes	Exposure B	30	20	20	6000	Bachelor	Bachelor	Orthodox	Georgian	City/Town
Low Risk	0	17	Male	No	Not Expose	30	30	30	1500	Bachelor	Master or I	Orthodox	Georgian	City/Town
Low Risk	1	19	Male	Yes	Exposure B	30	30	30	3000	High Schoo	High Schoo	Orthodox	Georgian	Village
Low Risk	2	24	Male	Yes	Exposure B	90	180	180	5000	Bachelor	Bachelor	Orthodox	Georgian	Emigrant (West)
Low Risk	0	20	Female	No	Exposure B	30	30	30	6000	Master or I	Bachelor	Orthodox	Georgian	City/Town
Low Risk	1	24	Female	Yes	Exposure B	90	90	90	3000	Bachelor	Master or I	Muslim	Georgian	City/Town
Low Risk	0	24	Male	No	Exposure B	180	180	90	1500	Bachelor	Master or I	Muslim	Georgian	City/Town
Low Risk	0	20	Female	No	Exposure B	30	30	30	3500	High Schoo	Master or I	Orthodox	Georgian	Village
Low Risk	0	25	Female	No	Not Expose	0	20	40	3000	Master or I	Bachelor	Orthodox	Georgian	City/Town
Low Risk	0	20	Female	No	Exposure B	39	90	90	700	Master or I	Bachelor	Orthodox	Georgian	City/Town
Low Risk	0	19	Male	Yes	Exposure B	30	30	30	5000	Bachelor	Bachelor	Orthodox	Georgian	City/Town
Low Risk	1	24	Female	No	Not Expose	90	90	90	3000	Bachelor	Bachelor	Orthodox	Georgian	City/Town
Low Risk	0	20	Female	No	Exposure B	10	60	120	4000	Bachelor	PhD	Orthodox	Georgian	City/Town
Low Risk	0	19	Female	Yes	Exposure B	30	150	30	1500	Bachelor	Bachelor	Orthodox	Georgian	Village
Low Risk	0	20	Female	No	Not Expose	0	10	15	6000	Bachelor	High Schoo	Orthodox	Georgian	City/Town
Low Risk														
Low Risk	0	19	Male	Yes	Exposure B	90	90	90	3000	Master or I	Master or I	Orthodox	Georgian	City/Town
Low Risk	0	20	Male	No	Not Expose	30	25	35	3000	Bachelor	Master or I	Orthodox	Georgian	City/Town
Low Risk	1	23	Male	No	Exposure B	30	90	90	500			Orthodox	Georgian	City/Town
High Risk	13	30	Female	No	Exposure B	60	360	500	3000	High Schoo	High Schoo	Muslim	Georgian	Village
Low Risk	0	24	Male	No	Exposure B	30	90	180	3000	Master or I	Master or I	Orthodox	Georgian	City/Town
Low Risk	0	22	Male	Yes	Exposure B	30	180	360	5000	Bachelor	High Schoo	Orthodox	Georgian	City/Town
Low Risk	1	18	Male	No	Not Expose	0	0	0	1500	Master or I	High Schoo	Orthodox	Georgian	City/Town
Low Risk	0	26	Male	No	Exposure B	90	180	90	1500	Bachelor	Master or I	Muslim	Georgian	City/Town
Low Risk	0	20	Male	No	Not Expose	25	25	20	3000	Bachelor	Master or I	Orthodox	Georgian	City/Town
Low Risk	0	21	Male	Yes	Exposure B	180	180	180	1500	Bachelor	Bachelor	Orthodox	Georgian	City/Town
Low Risk	2	19	Male	No	Exposure B	30	90	180	1500	High Schoo	Middle Sch	Orthodox	Georgian	City/Town
Low Risk	0	24	Male	No	Exposure B	30	30	30	3000	High Schoo	Bachelor	Orthodox	Georgian	City/Town
Low Risk	0	24	Female	No	Exposure B	30	30	90	1500	High Schoo	High Schoo	Orthodox	Georgian	City/Town
Low Risk	0	26	Male	No	Exposure B	30	30	30	5000	Master or I	Bachelor	Orthodox	Georgian	City/Town
Medium Ri	3	24	Male	No	Not Expose	90	180	180	1500	Bachelor	Bachelor	Orthodox	Georgian	City/Town
Low Risk	0	24	Female	No	Exposure B	30	90	180	2000	High Schoo	High Schoo	Orthodox	Georgian	Village
Low Risk	0	21	Male	No	Exposure B	30	25	15	2000	Bachelor	Bachelor	Orthodox	Georgian	Village
Low Risk	0	19	Female	No	Not Expose	0	0	0	0	High Schoo	Bachelor	Orthodox	Georgian	City/Town
Low Risk	0	24	Female	No	Exposure B	30	30	30	1500	Bachelor	High Schoo	Orthodox	Georgian	City/Town
Low Risk	0	29	Male	No	Exposure B	5	30	40	4000	Bachelor	Master or I	Orthodox	Georgian	City/Town
Low Risk	0	24	Male	No	Not Expose	0	0	0	0	Bachelor	Bachelor	Orthodox	Georgian	City/Town
Low Risk	1	26	Male	Yes	Exposure B	120	120	120	2000	Bachelor	Bachelor	Orthodox	Georgian	City/Town
Low Risk	1	25	Male	No	Exposure B	90	90	360	2000	Middle Sch	Middle Sch	Orthodox	Georgian	City/Town

Medium Ri	5	30	Female	Yes	Exposure B	130	130	130	1500	Bachelor	High Schoo	Orthodox	Georgian	Village
Low Risk	0	23	Male	Yes	Exposure B	30	30	30	4000	Master or I	Master or I	Orthodox	Georgian	City/Town
Low Risk	0	25	Male	Yes	Exposure B	0	90	90	8000	Bachelor	Bachelor	Orthodox	Georgian	City/Town
Medium Ri	7	17	Female	Yes	Exposure B	90	60	10	3000	Bachelor	Bachelor	Orthodox	Georgian	Emigrant (West)
Low Risk	0	23	Male	No	Exposure B	10	10	10	5000	Master or I	High Schoo	Orthodox	Georgian	City/Town
High Risk	10	21	Male	No	Exposure B	360	360	360	3000	High Schoo	High Schoo	Orthodox	Georgian	Village
Medium Ri	4	19	Male	Yes	Exposure B	180	180	180	3000	Bachelor	Bachelor	Orthodox	Georgian	City/Town
Low Risk	0	24	Male	No	Exposure B	10	10	20	5000	Master or I	High Schoo	Orthodox	Georgian	City/Town
Low Risk	0	24	Male	No	Exposure B	20	20	20	3000	Bachelor	Bachelor	Orthodox	Georgian	Emigrant (West)
Low Risk	0	18	Male	Yes	Exposure B	90	90	90	3500	Bachelor	Bachelor	Orthodox	Georgian	Village
Low Risk	1	20	Male	No	Exposure B	90	90	180	1000	Bachelor	Bachelor	Orthodox	Georgian	City/Town
Low Risk	0	29	Male	Yes	Exposure B	60	60	60	15000	Master or I	Master or I	Orthodox	Georgian	City/Town
Low Risk	2	18	Male	Yes	Exposure B	90	180	180	3000	Bachelor	Bachelor	Orthodox	Georgian	Village
Low Risk	0	18	Male	No	Not Expose	30	30	30	500	Bachelor	Bachelor	Orthodox	Georgian	City/Town
Low Risk	0	20	Female	Yes	Exposure B	60	45	30	3000	Bachelor	High Schoo	Orthodox	Georgian	City/Town
Medium Ri	7	30	Female	Yes	Exposure B	180	360	180	2000	High Schoo	High Schoo	Orthodox	Georgian	City/Town
Medium Ri	4	20	Male	No	Exposure B	90	90	30	6000	Bachelor	Bachelor	Orthodox	Georgian	City/Town
Low Risk	2	24	Male	Yes	Exposure B	30	30	90	1500	High Schoo	Middle Sch	Orthodox	Azerbaijan	City/Town
Low Risk	0	18	Male	No	Not Expose	0	0	0	0	Master or I	Master or I	Orthodox	Georgian	Emigrant (West)
High Risk	8	26	Male	Yes	Exposure B	180	360	90	1500	Bachelor	Middle Sch	Orthodox	Georgian	Village
Low Risk	1	25	Female	No	Not Expose	5	30	10	2000	Bachelor	High Schoo	Orthodox	Georgian	City/Town
Medium Ri	3	24	Male	No	Exposure B	30	30	30	3000	Master or I	Bachelor	Orthodox	Georgian	Emigrant (West)
Medium Ri	4	30	Male	No	Exposure B	90	90	90	3000	Middle Sch	Master or I	Orthodox	Georgian	City/Town
Low Risk	0	18	Male	No	Not Expose	30	30	30	1500	High Schoo	High Schoo	Orthodox	Georgian	Village
Low Risk	0	23	Male	No	Exposure B	40	10	5	10000	PhD	Master or I	Orthodox	Georgian	Emigrant (West)
High Risk	8	26	Male	Yes	Exposure B	360	360	360	1500	High Schoo	High Schoo	Orthodox	Other	Village
Low Risk	0	22	Male	No	Exposure B	60	360	360	2000	Bachelor	High Schoo	Orthodox	Georgian	Emigrant (West)
Low Risk	0	17	Male	Yes	Exposure B	30	30	30	1000	High Schoo	High Schoo	Orthodox	Georgian	Village
Low Risk	0	17	Male	Yes	Exposure B	30	30	30	1000	High Schoo	High Schoo	Orthodox	Georgian	Village
Low Risk	0	21	Female	No	Not Expose	30	30	30	3000	Bachelor	Bachelor	Orthodox	Georgian	Village
Medium Ri	3	24	Male	No	Exposure B	180	180	90	600	Bachelor	High Schoo	Orthodox	Georgian	City/Town
Low Risk	1	27	Female	Yes	Exposure B	360	360	360	2000	High Schoo	Bachelor	Orthodox	Georgian	City/Town
Low Risk	1	27	Female	Yes	Exposure B	90	90	180	1500	High Schoo	High Schoo	Orthodox	Georgian	City/Town
Low Risk	1	19	Female	Yes	Exposure B	20	20	20	5000	High Schoo	High Schoo	Orthodox	Georgian	Emigrant (West)
Low Risk	1	20	Male	Yes	Exposure B	0	60	120	8000	Bachelor	Bachelor	Orthodox	Georgian	City/Town
Low Risk	0	23	Female	Yes	Exposure B	20	30	30	3000	Master or I	Master or I	Orthodox	Georgian	Emigrant (West)
Low Risk	0	17	Male	No	Not Expose	8	10	5	2500	Bachelor	Bachelor	Orthodox	Georgian	City/Town
Low Risk	2	22	Male	Yes	Exposure B	20	20	20	2500	Bachelor	High Schoo	Orthodox	Georgian	Emigrant (West)
Low Risk	0	17	Female	Yes	Exposure B	60	60	60	2000	Bachelor	Master or I	Orthodox	Georgian	City/Town
Low Risk	0	23	Female	No	Not Expose	0	0	10	4000	PhD	Master or I	Orthodox	Georgian	City/Town
Low Risk	0	26	Male	No	Exposure B	90	30	20	3000	Bachelor	Master or I	Orthodox	Georgian	City/Town
Medium Ri	4	27	Female	Yes	Exposure B	20	30	30	3000	Bachelor	Bachelor	Orthodox	Georgian	City/Town
Low Risk	0	18	Female	No	Exposure B	30	30	30	3000	Master or I	Master or I	Orthodox	Georgian	Emigrant (West)
Low Risk	0	25	Female	No	Exposure B	60	180	180	6000	Master or I	Master or I	Orthodox	Georgian	City/Town

Low Risk	1	22	Female	Yes	Exposure B	30	30	30	1500	Bachelor	Master or I	Orthodox	Georgian	City/Town
High Risk	10	30	Male	No	Exposure B	30	30	30	1500	Bachelor	Master or I	Orthodox	Georgian	City/Town
Low Risk	1	18	Male	No	Not Expose	180	90	90	3000	Bachelor	High Schoo	Orthodox	Georgian	Emigrant (West)
Low Risk	1	17	Male	Yes	Exposure B	360	360	360	3000	Master or I	Bachelor	Orthodox	Georgian	Emigrant (West)
Low Risk	0	18	Male	Yes	Exposure B	5	30	40	1500	High Schoo	High Schoo	Orthodox	Georgian	City/Town
Medium Ri	7	23	Male	No	Exposure B	10	180	90		Master or I	Master or I	Orthodox	Georgian	City/Town
Medium Ri	6	28	Male	Yes	Exposure B	60	60	60	2000	High Schoo	High Schoo	Catholic	Georgian	City/Town
Low Risk	0	17	Male	Yes	Exposure B	30	30	30		Bachelor	Bachelor	Orthodox	Georgian	City/Town
Low Risk	2	17	Male	Yes	Exposure B	180	90	30	4000	Middle Sch	Middle Sch	Orthodox	Georgian	City/Town
Low Risk	0	18	Male	Yes	Exposure B	90	90	180	3000	Bachelor	High Schoo	Orthodox	Russian	Emigrant (West)
Low Risk	1	28	Male	Yes	Exposure B	180	180	135	1500	Middle Sch	High Schoo	Orthodox	Georgian	City/Town
Low Risk	0	21	Male	No	Exposure B	30	30	30	3000	Bachelor	Bachelor	Orthodox	Georgian	Emigrant (West)
Medium Ri	5	25	Female	Yes	Exposure B	15	15	15	2000	Bachelor	Bachelor	Orthodox	Georgian	City/Town
Low Risk	0	22	Female	No	Not Expose	5	5	5	3000	Master or I	Master or I	Orthodox	Georgian	City/Town
Low Risk	0	21	Female	Yes	Exposure B	90	120	180	1700	Bachelor	High Schoo	Orthodox	Georgian	City/Town
Low Risk	0	26	Female	No	Exposure B	30	30	30		Bachelor	Bachelor	Orthodox	Georgian	City/Town
Low Risk	1	18	Female	No	Exposure B	10	10	0	1500	High Schoo	High Schoo	Orthodox	Georgian	City/Town
Low Risk	0	22	Female	Yes	Exposure B	120	90	60	4000	Master or I	Master or I	Orthodox	Georgian	City/Town
Low Risk	0	25	Female	No	Not Expose	5	30	30	1500	High Schoo	Middle Sch	Other	Georgian	City/Town
Medium Ri	3	23	Male	Yes	Exposure B	30	30	30	5000	High Schoo	Bachelor	Orthodox	Georgian	City/Town
Low Risk	0	25	Male	No	Exposure B	15	5	0	3000	Bachelor	High Schoo	Other	Georgian	Emigrant (West)
Low Risk														
Medium Ri	6	30	Male	Yes	Exposure B	150	150	150	1800	Bachelor	High Schoo	Orthodox	Mixed	Emigrant (West)
High Risk	11	23	Male	Yes	Exposure B	90	90	90	1500	Bachelor	Bachelor	Orthodox	Other	City/Town
Low Risk	0	18	Female	No	Not Expose	30	30	30	3000	High Schoo	Middle Sch	Orthodox	Armenian	Emigrant (West)
High Risk	13	30	Male	Yes	Exposure B	360	360	360	4000	PhD	Master or I	Orthodox	Georgian	City/Town
Medium Ri	4	24	Male	No	Exposure B	360	360	240	1500	Middle Sch	Middle Sch	Orthodox	Georgian	Emigrant (West)
Low Risk	0	19	Male	No	Exposure B	30	90	90	3000	Bachelor	Bachelor	Orthodox	Georgian	City/Town
Medium Ri	6	30	Male	Yes	Exposure B	240	240	240	5000	Bachelor	Bachelor	Orthodox	Georgian	City/Town
Low Risk	0	26	Male	No	Exposure B	30	30	30	3000	Master or I	High Schoo	Orthodox	Georgian	City/Town
Low Risk	0	23	Male	No	Not Expose	0	0	0	5000	High Schoo	High Schoo	Orthodox	Georgian	Emigrant (West)
Medium Ri	3	22	Male	No	Exposure B	30	180	360	2000	High Schoo	Middle Sch	Orthodox	Georgian	Village
Low Risk	0	17	Female	No	Not Expose	0	0	0	1500	Bachelor	Bachelor	Muslim	Georgian	City/Town
Medium Ri	4	19	Male	No	Exposure B	30	30	30	3000	Bachelor	High Schoo	Orthodox	Georgian	Emigrant (West)
Medium Ri	3	17	Male	No	Exposure B	15	10	10	1500	Bachelor	Middle Sch	Orthodox	Georgian	Village
Low Risk	0	20	Female	No	Exposure B	15	30	360	3000	Bachelor	High Schoo	Orthodox	Georgian	City/Town
Low Risk	0	19	Female	No	Not Expose	0	0	0	700	High Schoo	High Schoo	Orthodox	Georgian	Village
Low Risk	1	24	Female	No	Exposure B	10	60	70	3000	Master or I	Bachelor	Orthodox	Mixed	City/Town
Medium Ri	3	30	Female	No	Exposure B	180	180	180	1500	Bachelor	Middle Sch	Muslim	Georgian	Village
Medium Ri	3	30	Male	No	Exposure B	10	10	10	2000	Bachelor	Bachelor	Protestant	Georgian	Emigrant (West)
Low Risk	1	17	Female	Yes	Exposure B	90	30	90	5000	Master or I	Master or I	Orthodox	Georgian	City/Town
Low Risk	1	24	Male	No	Exposure B	30	30	30	1500	Master or I	Bachelor	Orthodox	Georgian	City/Town
Low Risk	0	28	Female	No	Exposure B	30	30	30	3000	High Schoo	Bachelor	Orthodox	Georgian	City/Town
Low Risk	0	26	Female	Yes	Exposure B	360	360	360	1500	Middle Sch	High Schoo	Orthodox	Georgian	Emigrant (West)

Low Risk	0	18	Female	No	Exposure B	30	30	30	6000	Master or I	Bachelor	Orthodox	Georgian	Emigrant (West)
Low Risk	0	26	Male	No	Exposure B	20	20	20	3500	Bachelor	Bachelor	Orthodox	Georgian	City/Town
Low Risk	0	28	Male	No	Exposure B	30	30	90	1500	High Schoo	High Schoo	Orthodox	Georgian	
Medium Ri	4	22	Male	No	Exposure B	30	180	360	2000	High Schoo	Middle Sch	Orthodox	Georgian	Village
Low Risk	0	24	Male	No	Exposure B	90	180	180	1500	Bachelor	Bachelor	Orthodox	Georgian	City/Town
Low Risk	0	17	Male	Yes	Exposure B	30	15	15	500	High Schoo	High Schoo	Orthodox	Georgian	City/Town
Medium Ri	3	18	Male	No	Not Expose	180	180	180	3000	High Schoo	High Schoo	Orthodox	Georgian	City/Town
Low Risk	0	24	Male	No	Exposure B	10	10	10	5000	Bachelor	High Schoo	Orthodox	Georgian	City/Town
Medium Ri	5	17	Male	Yes	Exposure B	90	90	180	1500	Bachelor	Bachelor	Orthodox	Georgian	City/Town
Low Risk	0	29	Male	No	Exposure B	30	30	90	3000	High Schoo	High Schoo	Orthodox	Georgian	City/Town
Low Risk	0	25	Male	Yes	Exposure B	50	50	50	1000	High Schoo	High Schoo	Orthodox	Georgian	Village
Low Risk	0	22	Female	Yes	Exposure B	60	60	30	6000	Bachelor	Bachelor	Orthodox	Georgian	Emigrant (West)
Low Risk	0	21	Male	No	Exposure B	5	90	90	4500	Master or I	High Schoo	Orthodox	Georgian	City/Town
Low Risk	0	17	Female	Yes	Exposure B	30	180	180	5000	Bachelor	Bachelor	Orthodox	Georgian	City/Town
Low Risk	0	19	Female	No	Exposure B	60	30	10	9000	Bachelor	Bachelor	Orthodox	Georgian	City/Town
High Risk	13	24	Female	Yes	Exposure B	180	90	90	3000	Bachelor	Master or I	Orthodox	Georgian	City/Town
Low Risk	0	20	Male	No	Exposure B	30	210	360		Bachelor	High Schoo	Orthodox	Georgian	City/Town
Low Risk	1	17	Male	Yes	Exposure B	30	15	15	500	High Schoo	High Schoo	Orthodox	Georgian	City/Town
Low Risk	1	18	Male	No	Exposure B	30	30	30	2000	Bachelor	High Schoo	Orthodox	Georgian	City/Town
Low Risk	1	17	Female	No	Exposure B	10	30	20	6000	Bachelor	Bachelor	Orthodox	Georgian	City/Town
Low Risk	2	17	Male	Yes	Exposure B	30	30	30	4000	High Schoo	Bachelor	Orthodox	Georgian	City/Town
Low Risk														
Medium Ri	5	30	Female	Yes	Exposure B	60	60	60	0	Master or I	Master or I	Orthodox	Georgian	City/Town
Medium Ri	3	30	Female	No	Exposure B	180	180	180	1500	High Schoo	High Schoo	Muslim	Other	Village
Low Risk	1	27	Male	No	Exposure B	60	60	60	1000	Bachelor	Bachelor	Orthodox	Georgian	Village
High Risk	11	30	Female	Yes	Exposure B	180	180	180	1500	Bachelor	High Schoo	Orthodox	Georgian	City/Town
Low Risk	1	29	Female	Yes	Exposure B	180	180	180	3000	Master or I	Bachelor	Orthodox	Georgian	Emigrant (West)
Low Risk	1	18	Male	No	Exposure B	10	90	180	3000	High Schoo	High Schoo	Orthodox	Georgian	City/Town
Low Risk	0	22	Male	No	Not Expose	0	0	0	5000	Master or I	Bachelor	Orthodox	Georgian	City/Town
Low Risk	1	26	Female	Yes	Exposure B	180	360	360	3000	Bachelor	Bachelor	Other	Georgian	City/Town
Medium Ri	7	19	Female	Yes	Exposure B	15	180	360	2000	High Schoo	High Schoo	Orthodox	Georgian	City/Town
Low Risk	0	20	Female	No	Not Expose	0	0	0	0	Master or I	Master or I	Orthodox	Georgian	City/Town
Low Risk	1	24	Male	No	Exposure B	5	0	0	1000	High Schoo	High Schoo	Other	Georgian	City/Town
Low Risk	0	26	Male	No	Exposure B	60	60	30	2500	Master or I	Bachelor	Muslim	Georgian	City/Town
Low Risk	0	21	Male	Yes	Exposure B	180	90	30	1500	High Schoo	Middle Sch	Muslim	Azerbaijan	Village
Low Risk	0	26	Male	Yes	Exposure B	90	90	90	2500	Master or I	Bachelor	Orthodox	Georgian	City/Town
Low Risk	0	19	Female	Yes	Exposure B	120	360	360	2000	Bachelor	High Schoo	Orthodox	Georgian	City/Town
Low Risk	0	21	Female	No	Exposure B	60	40	60	3000	Bachelor	Master or I	Orthodox	Georgian	City/Town
Low Risk	2	20	Male	Yes	Exposure B	90	180	360	1500	High Schoo	High Schoo	Orthodox	Georgian	Village
Low Risk	0	26	Female	No	Exposure B	90	90	90	6000	Master or I	Bachelor	Orthodox	Georgian	City/Town
Low Risk	0	18	Male	No	Not Expose	0	5	5	5000	Bachelor	Bachelor	Orthodox	Georgian	City/Town
Low Risk	0	24	Female	No	Exposure B	30	30	30	1500	Bachelor	Bachelor	Orthodox	Georgian	City/Town
Low Risk	0	19	Male	No	Not Expose	4	24	50	3000	Master or I	Master or I	Orthodox	Georgian	City/Town
Low Risk	0	20	Male	Yes	Exposure B	360	360	360	1750	Bachelor	Bachelor	Orthodox	Georgian	City/Town

Low Risk	2	23	Male	No	Not Expose	90	600		7000	Bachelor	Bachelor	Orthodox	Georgian	City/Town
Low Risk	0	17	Female	Yes	Exposure B	90	180	180	4000	Bachelor	Bachelor	Muslim	Azerbaijan	City/Town
Low Risk	1	18	Female	No	Not Expose	10	15	5	6000	Master or I	Master or I	Orthodox	Georgian	City/Town
Low Risk	1	18	Male	Yes	Exposure B	45	45	0	3000	High Schoo	Bachelor	Orthodox	Georgian	City/Town
Low Risk	0	22	Male	No	Not Expose	0	0	0	1500	Bachelor	Bachelor	Orthodox	Georgian	City/Town
Low Risk	1	18	Male	No	Exposure B	15	30	30	4500	High Schoo	High Schoo	Orthodox	Georgian	Emigrant (West)
Low Risk	2	24	Female	No	Not Expose	5	5	0	1500	Bachelor	High Schoo	Orthodox	Georgian	City/Town
Medium Ri	5	30	Male	Yes	Exposure B	90	360	360	5000	Master or I	PhD	Orthodox	Georgian	City/Town
Low Risk	0	20	Female	No	Exposure B	10	90	180	3000	Master or I	High Schoo	Orthodox	Georgian	City/Town
Low Risk	0	27	Female	No	Exposure B	90	90	30	3000	Bachelor	Master or I	Orthodox	Georgian	Village
Low Risk	0	25	Female	No	Exposure B	30	30	30	1500	Bachelor	Bachelor	Orthodox	Georgian	Emigrant (West)
Low Risk	1	24	Female	No	Exposure B	30	360	360	4000	Bachelor	Bachelor	Orthodox	Georgian	City/Town
Low Risk	1	23	Female	No	Exposure B	30	90	15	2000	Bachelor	High Schoo	Orthodox	Georgian	City/Town
Medium Ri	5	17	Male	Yes	Exposure B	90	360	180	3000	Bachelor	Bachelor	Orthodox	Georgian	City/Town
Low Risk	1	21	Female	No	Not Expose	45	46	45	1500	Bachelor	High Schoo	Protestant	Georgian	City/Town
Low Risk	1	17	Female	Yes	Exposure B	20	20	15	3000	Bachelor	High Schoo	Orthodox	Georgian	City/Town
Medium Ri	5	26	Male	Yes	Exposure B	30	30	180	1500	Middle Sch	Bachelor	Orthodox	Georgian	City/Town
Low Risk	0	19	Male	No	Not Expose	0	0	0		Master or I	High Schoo	Orthodox	Georgian	City/Town
Low Risk	0	28	Female	No	Exposure B	90	30	30	3000	Bachelor	Bachelor	Orthodox	Georgian	City/Town
Low Risk	0	21	Female	No	Exposure B	10	15	15	3000	High Schoo	Bachelor	Orthodox	Georgian	Village
Low Risk	0	29	Male	No	Not Expose	20	20	20	1500	Bachelor	High Schoo	Orthodox	Georgian	Mountainous Region
Low Risk	0	25	Female	No	Not Expose	3	100	180	3500	Master or I	High Schoo	Orthodox	Georgian	City/Town
Low Risk	0	17	Female	No	Exposure B	10	10	5	4000	Bachelor	Bachelor	Orthodox	Georgian	City/Town
Low Risk	1	26	Male	No	Exposure B	20	30	30	1000	Master or I	Master or I	Orthodox	Georgian	City/Town
Low Risk	1	18	Female	No	Exposure B	180	90	30	8000	Master or I	Bachelor	Orthodox	Georgian	City/Town
Low Risk	0	21	Male	No	Not Expose	0	0	0	2300	Bachelor	Bachelor	Orthodox	Georgian	City/Town
Low Risk	2	25	Male	No	Exposure B	90	90	90	1500	Master or I	Bachelor	Orthodox	Georgian	Emigrant (West)
Low Risk	1	17	Male	Yes	Exposure B	90	90	90	3000	High Schoo	Bachelor	Orthodox	Georgian	City/Town
Medium Ri	5	26	Female	Yes	Exposure B	30	90	90	3000	Master or I	Master or I	Orthodox	Georgian	City/Town
Low Risk	1	18	Male	No	Exposure B	15	5	5	5000	Master or I	High Schoo	Orthodox	Georgian	Emigrant (West)
Low Risk	0	29	Male	No	Not Expose	30	90	360	3000	Bachelor	Bachelor	Orthodox	Georgian	City/Town
Medium Ri	6	23	Male	Yes	Exposure B	360	360	360	2000	Bachelor	Bachelor	Orthodox	Georgian	City/Town
Low Risk	1	21	Male	No	Not Expose	0	0	0	2200	Bachelor	Bachelor	Orthodox	Georgian	City/Town
Low Risk	1	29	Male	Yes	Exposure B	90	90	90	4000	High Schoo	High Schoo	Orthodox	Georgian	Emigrant (West)
Medium Ri	5	22	Female	Yes	Exposure B	90	90	90	3000	Bachelor	High Schoo	Muslim	Georgian	Village
Low Risk	0	27	Male	Yes	Exposure B	90	90	90	3000	Master or I	Bachelor	Orthodox	Georgian	City/Town
Low Risk	0	21	Male	No	Not Expose	0	0	0	1500	Master or I	Bachelor	Orthodox	Georgian	City/Town
Medium Ri	3	29	Female	No	Exposure B	30	30	30	2500	Bachelor	Bachelor	Orthodox	Georgian	Emigrant (West)
Low Risk	0	17	Female	No	Not Expose	0	0	0	3000	Bachelor	Bachelor	Orthodox	Georgian	City/Town
Low Risk	1	18	Male	No	Exposure B	30	30	30	3000	Master or I	Bachelor	Orthodox	Georgian	City/Town
Low Risk	0	18	Female	Yes	Exposure B	30	30	30	1000	High Schoo	High Schoo	Other	Georgian	Emigrant (Asia/Russia/Africa)
Low Risk	0	18	Female	No	Not Expose	5								
Low Risk	0	24	Male	No	Not Expose	0	30	0	1500	High Schoo	High Schoo	Muslim	Mixed	Emigrant (West)
Low Risk	0	16	Female	No	Exposure B	30	90	180	3000	Bachelor	Bachelor	Orthodox	Georgian	City/Town

Low Risk	1	30	Female	Yes	Exposure B	90	90	90	3000	Bachelor	Bachelor	Orthodox	Georgian	City/Town
Low Risk	0	16	Male	No	Not Expose	10	10	0	3000	High Schoo	Bachelor	Orthodox	Georgian	Emigrant (West)
Low Risk														
Low Risk	2	30	Male	Yes	Exposure B	30	180	220	3000	Bachelor	High Schoo	Orthodox	Georgian	Village
Low Risk	0	30	Male	No	Exposure B	90	90	90	1500	Bachelor	Bachelor	Orthodox	Georgian	City/Town
Low Risk	1	21	Male	Yes	Exposure B	90	60	30	10000	Master or	High Schoo	Atheist	Mixed	City/Town
Medium Ri	3	19	Male	Yes	Exposure B	60	60	20	1500	Bachelor	Bachelor	Orthodox	Georgian	City/Town
Low Risk	0	23	Female	No	Exposure B	15	10	5	3000	Bachelor	Bachelor	Orthodox	Georgian	City/Town
Low Risk	0	16	Male	No	Exposure B	15	60	15	3000	Master or	Master or	Orthodox	Georgian	City/Town
Low Risk	0	18	Female	Yes	Exposure B	30	30	30	3000	High Schoo	High Schoo	Orthodox	Georgian	Emigrant (West)
Low Risk	1	18	Male	No	Not Expose	15	90	90	2000	High Schoo	High Schoo	Orthodox	Georgian	City/Town
Low Risk	1	30	Female	No	Not Expose	30	60	30	2000	Master or	Bachelor	Orthodox	Georgian	City/Town
Low Risk	0	16	Female	No	Not Expose	0	20	20	3000	Bachelor	High Schoo	Muslim	Georgian	Village
Low Risk	0	21	Male	Yes	Exposure B	90	90	30	6000	Bachelor	Bachelor	Orthodox	Georgian	City/Town
Low Risk	1	24	Female	No	Not Expose	5	30	60	2000	Bachelor	High Schoo	Orthodox	Georgian	City/Town
Low Risk	0	16	Female	No	Not Expose	3	10	10	3000	Master or	High Schoo	Orthodox	Georgian	City/Town
Low Risk	1	21	Female	Yes	Exposure B	60	60	180	4000	High Schoo	High Schoo	Orthodox	Georgian	City/Town
Medium Ri	4	23	Female	No	Not Expose	30	90	60	800	High Schoo	Bachelor	Orthodox	Georgian	City/Town
Low Risk	0	21	Female	Yes	Exposure B	90	90	90	3000	Master or	Bachelor	Orthodox	Georgian	City/Town
Low Risk	0	16	Female	No	Exposure B	3	30	30		High Schoo	Master or	Orthodox	Georgian	City/Town
Low Risk	0	30	Male	No	Exposure B	45	45	45	9000	Bachelor	Bachelor	Orthodox	Georgian	City/Town
Low Risk	0	20	Male	Yes	Exposure B	30	30	30	3000	High Schoo	High Schoo	Orthodox	Georgian	City/Town
Low Risk	0	16	Male	Yes	Exposure B	90	30	30	3000	Bachelor	High Schoo	Orthodox	Georgian	City/Town
Low Risk	1	30	Male	No	Exposure B	30	30	30	1000	Master or	Master or	Orthodox	Georgian	City/Town
Low Risk	0	23	Female	No	Not Expose	5	30	90	3000	Master or	Bachelor	Orthodox	Georgian	City/Town
Medium Ri	6	25	Male	No	Not Expose	30	30	30	3000	Bachelor	Bachelor	Orthodox	Georgian	Village
Low Risk	0	30	Female	No	Not Expose	0	0	0	5500	Bachelor	Bachelor	Orthodox	Georgian	City/Town
Low Risk	1	16	Male	No	Exposure B	30	90	90	4000	Bachelor	Bachelor	Orthodox	Georgian	City/Town
Low Risk	0	26	Male	Yes	Exposure B	90	90	90	3000	High Schoo	High Schoo	Orthodox	Georgian	City/Town
Medium Ri	3	30	Male	Yes	Exposure B	30	180	90	1500	Bachelor	Bachelor	Orthodox	Georgian	City/Town
High Risk	15	30	Male	No	Not Expose	30	30	30	3000	Bachelor	Bachelor	Orthodox	Georgian	City/Town
Low Risk	0	27	Female	No	Not Expose	20	20	10	2600	High Schoo	High Schoo	Orthodox	Georgian	City/Town
Low Risk	1	30	Female	No	Exposure B	30	180	180	1000	Bachelor	High Schoo	Other	Georgian	City/Town
Low Risk	1	27	Male	Yes	Exposure B	30	30	120	5000	Master or	Master or	Orthodox	Georgian	City/Town
Low Risk	0	16	Male	No	Not Expose	10	10	10	1900	Bachelor	High Schoo	Orthodox	Armenian	City/Town
Low Risk	1	16	Male	No	Not Expose	0	0	0	4000	Master or	Bachelor	Orthodox	Georgian	City/Town
Low Risk	1	20	Male	No	Not Expose	0	30	30	1500	Bachelor	High Schoo	Orthodox	Georgian	City/Town
Low Risk	1	22	Female	No	Exposure B	30	30	30	3000	Master or	Bachelor	Orthodox	Georgian	City/Town
Low Risk	0	23	Female	No	Exposure B	30	15	7	6000	Bachelor	Master or	Orthodox	Georgian	City/Town
Low Risk	0	25	Female	Yes	Exposure B	60	60	45	2000	Bachelor	High Schoo	Orthodox	Georgian	City/Town
Low Risk	0	17	Male	Yes	Exposure B	30	30	30	2500	Bachelor	Bachelor	Orthodox	Georgian	City/Town
Low Risk	1	30	Female	No	Exposure B	30	30	30	5000	Bachelor	Bachelor	Orthodox	Georgian	Emigrant (West)
Low Risk	1	16	Male	Yes	Exposure B	30	30	30	2000	Bachelor	High Schoo	Orthodox	Georgian	Emigrant (West)
Low Risk	0	27	Female	Yes	Exposure B	90	90	90	5000	Master or	Master or	Orthodox	Georgian	Emigrant (West)

Low Risk	1	22	Male	Yes	Exposure B	90	90	180	2500	High Schoo	High Schoo	Orthodox	Georgian	City/Town
Low Risk	0	21	Female	Yes	Exposure B	90	360	180	1500	Bachelor	Middle Sch	Orthodox	Georgian	Village
Low Risk	0	24	Male	Yes	Exposure B	90	90	90	4000	Bachelor	Bachelor	Orthodox	Georgian	City/Town
Medium Ri	3	30	Male	Yes	Exposure B	90	180	360	3000	High Schoo	High Schoo	Muslim	Georgian	Village
Low Risk	0	19	Female	No	Exposure B	90	30	30	3000	High Schoo	High Schoo	Orthodox	Georgian	City/Town
Medium Ri	3	30	Male	Yes	Exposure B	0	30	90	500	Bachelor	Bachelor	Orthodox	Georgian	Emigrant (West)
Low Risk	1	28	Female	Yes	Exposure B	600	0	360	3000	Bachelor	Middle Sch	Orthodox	Georgian	Emigrant (West)
Low Risk	0	30	Female	No	Exposure B	30	30	60	3000	Master or I	Master or I	Orthodox	Georgian	City/Town
Low Risk	1	16	Female	No	Not Expose	0	15	0	3000	Bachelor	Bachelor	Orthodox	Georgian	Emigrant (West)
Low Risk	2	19	Male	No	Exposure B	30	360	360	1500	Bachelor	Master or I	Orthodox	Georgian	City/Town
Low Risk	1	26	Male	Yes	Exposure B	30	90	90	2000	Master or I	Master or I	Orthodox	Georgian	City/Town
Low Risk	0	18	Male	No	Exposure B	30	30	30	3000	High Schoo	High Schoo	Orthodox	Georgian	Emigrant (West)
Medium Ri	5	30	Female	Yes	Exposure Before 6 Months					Middle Sch	High Schoo	Orthodox	Georgian	Mountainous Region
Low Risk	0	30	Female	No	Exposure B	30	180	360	0	Bachelor	High Schoo	Orthodox	Georgian	Village
Medium Ri	3	16	Male	No	Exposure B	30	200	30	3500	Bachelor	Bachelor	Orthodox	Georgian	City/Town
Medium Ri	3	30	Male	Yes	Exposure B	90	180	360	3000	Bachelor	High Schoo	Orthodox	Georgian	Village
Low Risk	1	30	Female	No	Exposure B	90	90	180	3000	Master or I	Middle Sch	Orthodox	Georgian	City/Town
Low Risk	0	18	Male	No	Not Expose	10	10	10	1500	Bachelor	Master or I	Orthodox	Georgian	Village
Low Risk	2	30	Male	Yes	Exposure B	60	60	360	3000	Bachelor	Master or I	Orthodox	Georgian	City/Town
Low Risk	1	18	Male	No	Not Expose	15	15	15	3000	Bachelor	Bachelor	Orthodox	Georgian	City/Town
Low Risk	1	27	Male	Yes	Exposure B	30	90	90	3000	Master or I	High Schoo	Orthodox	Georgian	City/Town
Low Risk	0	23	Male	No	Not Expose	0	0	0	1500	Master or I	High Schoo	Orthodox	Georgian	City/Town
Low Risk	0	18	Male	Yes	Exposure B	30	30	30	3000	Bachelor	Bachelor	Orthodox	Armenian	City/Town
Low Risk	0	20	Male	No	Not Expose	15	60	90	3000	Bachelor	Bachelor	Orthodox	Georgian	City/Town
Medium Ri	3	16	Female	No	Not Expose	0	0	0	3000	Master or I	Bachelor	Orthodox	Georgian	City/Town
Low Risk	2	23	Male	No	Exposure B	40	40	40	3000	Bachelor	High Schoo	Orthodox	Georgian	City/Town
Low Risk	2	19	Female	Yes	Exposure B	180	180	120	5000	Master or I	High Schoo	Orthodox	Georgian	City/Town
Low Risk	0	21	Female	Yes	Exposure B	30	180	30	1500	Bachelor	Middle Sch	Orthodox	Georgian	Village
Low Risk	0	18	Male	Yes	Exposure B	5	30	90	1500	High Schoo	High Schoo	Orthodox	Georgian	City/Town
Low Risk	0	29	Male	No	Exposure B	60	60	45	1500	High Schoo	High Schoo	Orthodox	Georgian	City/Town
Low Risk	1	16	Male	No	Not Expose	30	90	90	1500	High Schoo	Middle Sch	Orthodox	Georgian	Village
Low Risk	0	30	Female	No	Exposure B	30	30	15	3000	Master or I	Master or I	Orthodox	Georgian	Village
Low Risk	1	24	Male	No	Exposure B	30	30	30	5000	Bachelor	Bachelor	Other	Georgian	City/Town
Low Risk	1	16	Female	Yes	Exposure B	30	360	430	3000	Master or I	Master or I	Orthodox	Georgian	City/Town
Low Risk	0	16	Male	Yes	Exposure B	180	180	500	10000	Bachelor	High Schoo	Orthodox	Georgian	City/Town
Low Risk	0	23	Male	Yes	Exposure B	90	90	90	1500	Bachelor	Bachelor	Orthodox	Georgian	Emigrant (West)
Low Risk	0	22	Male	No	Exposure B	25	25	25	8000	Master or I	Bachelor	Orthodox	Georgian	City/Town
Low Risk	0	24	Female	Yes	Exposure B	40	90	180	1500	High Schoo	High Schoo	Orthodox	Georgian	City/Town
Low Risk	0	16	Male	No	Not Expose	0	0	0	3000	Bachelor	Bachelor	Orthodox	Georgian	Village
Low Risk	0	22	Female	Yes	Exposure B	180	180	180	7000	Bachelor	High Schoo	Orthodox	Mixed	Emigrant (West)
Low Risk	1	30	Male	Yes	Exposure B	90	30	90	2500	Bachelor	Bachelor	Orthodox	Georgian	City/Town
Low Risk	0	30	Male	No	Not Expose	180	180	180	7200	Master or I	Master or I	Orthodox	Georgian	City/Town
Low Risk	0	28	Female	No	Not Expose	20	30	30	1300	Bachelor	High Schoo	Orthodox	Georgian	Village
Low Risk	0	16	Female	Yes	Exposure B	15	20	0	5000	Master or I	Bachelor	Orthodox	Georgian	City/Town

Low Risk	2	16	Male	Yes	Exposure B	60	60	60	3000	Bachelor	High Schoo	Orthodox	Georgian	Village
Low Risk	2	26	Male	No	Exposure B	10	15	40	3000	Bachelor	Bachelor	Orthodox	Georgian	City/Town
Low Risk	0		Male	Yes	Exposure B	180	180		1500	High Schoo	Bachelor	Orthodox		
Low Risk	0	21	Male	No	Not Expose	5	5	5		Master or I	Master or I	Orthodox	Georgian	City/Town
Low Risk	1	28	Male	Yes	Exposure B	180	180	360	10000	Bachelor	Bachelor	Orthodox	Georgian	City/Town
Low Risk	1	22	Female	No	Exposure B	30	180	360	1500	Bachelor	Bachelor	Orthodox	Georgian	City/Town
Low Risk	1	19	Male	No	Exposure B	90	360	360	3000	Bachelor	High Schoo	Orthodox	Georgian	City/Town
Low Risk	0			No	Exposure Between 12 and 18 Months									
Low Risk	0	24	Male	No	Not Expose	0	0	0	0	Bachelor	High Schoo	Orthodox	Georgian	City/Town
Low Risk	0	30	Female	No	Exposure B	30	90	90	1700	High Schoo	PhD	Orthodox	Georgian	City/Town
Medium Ri	6	30	Female	Yes	Exposure B	10			2000	Middle Sch	High Schoo	Orthodox	Georgian	Mountainous Region
Low Risk	1	24	Female	No	Not Expose	90	90	90	3000	Master or I	High Schoo	Orthodox	Georgian	Village
Low Risk	0	24	Male	No	Exposure B	15	90	90	5000	Master or I	Bachelor	Orthodox	Georgian	City/Town
Low Risk														
Low Risk	1	16	Female	No	Not Expose	0	0	0	3000	PhD	Bachelor	Orthodox	Georgian	City/Town
Low Risk	0	30	Male	No	Exposure B	30	30	30	1500	Master or I	Bachelor	Orthodox	Georgian	City/Town
Low Risk		26	Female											
Low Risk	0	28	Male	Yes	Exposure B	60	90	90	1500	Bachelor	Middle Sch	Orthodox	Georgian	City/Town
Low Risk	0	24	Female	No	Exposure B	15	90	120	2300	Bachelor	Master or I	Orthodox	Georgian	City/Town
Low Risk	0	25	Male	Yes	Exposure B	90	30	30	3000	High Schoo	High Schoo	Orthodox	Georgian	City/Town
Medium Ri	3	16	Male	No	Not Expose	0	0	0	2500	Bachelor	Bachelor	Orthodox	Georgian	City/Town
Low Risk	1	28	Male	No	Exposure B	20	60	90	500	Bachelor	Bachelor	Orthodox	Georgian	City/Town
Low Risk	0	30	Male	No	Exposure B	30	90	90	1500	Master or I	Middle Sch	Orthodox	Armenian	Village
Low Risk	1	16	Female	Yes	Exposure B	30	30	90	3000	Bachelor	High Schoo	Orthodox	Georgian	City/Town
Low Risk	0	28	Female	No	Not Expose	20	30	30	1300	Bachelor	High Schoo	Orthodox	Georgian	Village
Medium Ri	3	30	Female	No	Exposure B	30	180	360	3000	Bachelor	High Schoo	Orthodox	Georgian	City/Town
Low Risk	0	25	Female	No	Not Expose	30	30	30	3000	Bachelor	High Schoo	Muslim	Georgian	City/Town
Low Risk	0	21	Male	Yes	Exposure B	60	60	40	2000	Bachelor	High Schoo	Orthodox	Georgian	Mountainous Region
Low Risk	0	19	Male	No	Not Expose	0	0	0	150	High Schoo	High Schoo	Orthodox	Georgian	Village
Medium Ri	3	19	Male	No	Exposure B	10	180	360	3000	Bachelor	Bachelor	Orthodox	Georgian	City/Town
Low Risk	1	16	Female	No	Not Expose	0	0	0	3500	Bachelor	High Schoo	Orthodox	Georgian	City/Town
Low Risk	2	17	Male	No	Exposure B	30	30	30	3000	High Schoo	Bachelor	Orthodox	Georgian	City/Town
Low Risk	2	16	Male	Yes	Exposure B	2	25	25	4000	High Schoo	High Schoo	Muslim	Georgian	Emigrant (West)
High Risk	12	30	Male	No	Not Expose	180	180	90	500	Middle Sch	High Schoo	Orthodox	Georgian	Village
Low Risk	2	28	Female	No	Not Expose	180	180	360	5000	Bachelor	Bachelor	Orthodox	Georgian	City/Town
Low Risk	0	18	Female	Yes	Exposure B	90	90	180	3000	Bachelor	Bachelor	Orthodox	Mixed	City/Town
Low Risk	0	16	Male	No	Not Expose	30	180	180	3000	Bachelor	High Schoo	Orthodox	Georgian	City/Town
Medium Ri	3	30	Male	No	Not Expose	15	15	15	3000	Master or I	High Schoo	Orthodox	Georgian	Village
Low Risk	0	16	Female	No	Not Expose	0	0	0	3000	Bachelor	Bachelor	Orthodox	Georgian	City/Town
Low Risk	2	30	Female	No	Exposure B	17	20	17	2100	Bachelor	High Schoo	Orthodox	Georgian	Village
Low Risk	1	30	Male	No	Exposure B	5	20	30	3000	Bachelor	High Schoo	Orthodox	Georgian	Village
Low Risk	1	27	Male	No	Exposure B	20	20	25	1000	High Schoo	High Schoo	Orthodox	Georgian	Village
Low Risk	1	22	Female	Yes	Exposure B	60	180	180	2000	Master or I	High Schoo	Muslim	Georgian	City/Town
Low Risk	1	27	Male	No	Exposure B	30	90	90	609	Bachelor	High Schoo	Orthodox	Georgian	City/Town

Low Risk	1	24	Male	No	Not Expose	0	0	0	1000	High Schoo	High Schoo	Orthodox	Georgian	City/Town
Low Risk	0	24	Male	No	Not Expose	30	30	30	500	Bachelor	Bachelor	Orthodox	Georgian	Village
Low Risk	0	18	Female	No	Exposure B	20	20	20	1000	Master or I	Master or I	Orthodox	Georgian	Village
Low Risk	0	20	Female	No	Exposure B	15	15	15	4000	Master or I	Bachelor	Orthodox	Georgian	City/Town
Low Risk	2	30	Male	Yes	Exposure B	30	30	30	1500	Master or I	Master or I	Orthodox	Georgian	City/Town
Low Risk	1	16	Female	No	Not Expose	60	60	60	600	High Schoo	High Schoo	Orthodox	Georgian	City/Town
Low Risk	0	17	Male	Yes	Exposure B	30	30	30	3000	Bachelor	Bachelor	Orthodox	Georgian	City/Town
Low Risk	0	16	Male	No	Not Expose	0	0	0	4000	Bachelor	Bachelor	Orthodox	Georgian	City/Town
Low Risk	0	23	Female	Yes	Exposure B	60	180	360	300	Master or I	Master or I	Other	Georgian	Emigrant (West)
Low Risk	0	18	Male	No	Exposure B	30	180	360	0	Bachelor	Bachelor	Orthodox	Georgian	City/Town
Low Risk	1	16	Female	Yes	Exposure B	180	180	180	5000	High Schoo	High Schoo	Orthodox	Georgian	City/Town
High Risk	16	20	Female	No	Not Expose	30	30	30	500	High Schoo	High Schoo	Orthodox	Georgian	City/Town
Low Risk	1	16	Male	No	Exposure B	20	30	30	1500	Bachelor	Bachelor	Orthodox	Georgian	City/Town
Low Risk	0	19	Male	No	Exposure B	30	30	30	3000	Bachelor	Bachelor	Orthodox	Georgian	City/Town
Low Risk	0	18	Male	Yes	Exposure B	30	30	30	3000	High Schoo	High Schoo	Orthodox	Georgian	City/Town
Low Risk	0	18	Female	Yes	Exposure B	20	2	90	2000	Bachelor	Bachelor	Orthodox	Georgian	City/Town
Low Risk	0	22	Female	No	Exposure B	90	90	180	3000	Bachelor	Bachelor	Orthodox	Georgian	Village
Low Risk	1	16	Female	No	Not Expose	0	0	0		High Schoo	High Schoo	Orthodox	Georgian	City/Town
Medium Ri	3	18	Male	Yes	Exposure B	30	30	30	1500	High Schoo	High Schoo	Orthodox	Georgian	Emigrant (West)
Low Risk	2	16	Male	Yes	Exposure B	30	180	90	4000	Bachelor	Bachelor	Orthodox	Georgian	Village
Low Risk	0	29	Female	No	Exposure B	30	30	30	10000	Bachelor	Bachelor	Orthodox	Georgian	City/Town
Low Risk	0	29	Male	Yes	Exposure B	90	90	180	3000	Bachelor	Bachelor	Orthodox	Georgian	City/Town
Low Risk	0	19	Female	Yes	Exposure B	40	30	30	2500	Bachelor	Bachelor	Orthodox	Georgian	City/Town
High Risk	10	28	Male	Yes	Exposure B	120	120	120	1500	Master or I	High Schoo	Orthodox	Georgian	City/Town
Low Risk	0	17	Male	No	Not Expose	0	0	0		Master or I	Master or I	Orthodox	Georgian	City/Town
Low Risk	0	26	Male	No	Not Expose	40	40	40	9000	Master or I	High Schoo	Orthodox	Georgian	City/Town
Medium Ri	4	23	Male	Yes	Exposure B	180	180	180	600	Bachelor	High Schoo	Orthodox	Georgian	City/Town
Low Risk	1	22	Male	No	Not Expose	0	0	0	4000	Bachelor	Master or I	Orthodox	Georgian	City/Town
Low Risk	0	23	Female	Yes	Exposure B	30	30	30	2000	High Schoo	High Schoo	Orthodox	Georgian	City/Town
Low Risk	0	16	Female	No	Not Expose	30	30	30	2500	Bachelor	Bachelor	Orthodox	Georgian	City/Town
Medium Ri	5	16	Female	No	Exposure B	90	90	90	3000	Bachelor	Master or I	Orthodox	Georgian	City/Town
Low Risk	1	20	Male	No	Not Expose	5	5	5	3000	Bachelor	Bachelor	Orthodox	Georgian	City/Town
Low Risk	0	21	Male	No	Not Expose	0	0	0	5000	Master or I	Master or I	Orthodox	Georgian	City/Town
Low Risk	0	20	Female	Yes	Exposure B	120	120	150	6000	Master or I	Bachelor	Orthodox	Georgian	City/Town
Low Risk	1	18	Female	Yes	Exposure B	90	90	90	1500	High Schoo	High Schoo	Orthodox	Georgian	City/Town
Low Risk	1	28	Male	No	Exposure B	90	90	60	1500	Bachelor	High Schoo	Protestant	Georgian	Village
Low Risk	1	16	Female	No	Exposure B	15	15	30	2000	Bachelor	High Schoo	Orthodox	Georgian	City/Town
Low Risk	2	16	Female	Yes	Exposure B	30	60	60	2000	Bachelor	Bachelor	Orthodox	Georgian	City/Town
Low Risk	0	24	Female	No	Not Expose	30	30	30	1500	Master or I	Middle Sch	Orthodox	Georgian	Village
Low Risk	0	20	Male	No	Exposure B	30	360	500	3000	Master or I	Master or I	Orthodox	Georgian	City/Town
Low Risk	0	16	Male	No	Exposure B	30	30	30	10000	Master or I	Bachelor	Orthodox	Georgian	City/Town
Low Risk	0	22	Male	No	Exposure B	180	360	720	300	Bachelor	Bachelor	Orthodox	Georgian	Village
Low Risk	0	30	Male	No	Exposure B	20	20	20	900	Master or I	High Schoo	Muslim	Georgian	Village
Low Risk	1	27	Male	Yes	Exposure B	5	30	60	2000	Master or I	Master or I	Orthodox	Georgian	City/Town

Low Risk	0	16	Female	Yes	Exposure B	90	90	90	1500	Bachelor	Bachelor	Orthodox	Georgian	City/Town
Low Risk	1	16	Male	No	Exposure B	30	90	180	2500	Master or	Bachelor	Orthodox	Georgian	City/Town
Medium Ri	4	30	Male	No	Not Expose	30	30	30	3000	Bachelor	Bachelor	Orthodox	Georgian	City/Town
Low Risk	0	22	Male	Yes	Exposure B	5	90	200	1200	Bachelor	High Schoo	Orthodox	Georgian	City/Town
Low Risk	1	16	Female	No	Exposure B	30	90	120	2000	Bachelor	Bachelor	Orthodox	Georgian	City/Town
Low Risk	1	30	Male	No	Exposure B	60	0	0	2000	High Schoo	High Schoo	Orthodox	Georgian	Village
Medium Ri	3	25	Male	Yes	Exposure B	120	120	120	800	High Schoo	High Schoo	Orthodox	Georgian	City/Town
Low Risk	0	18	Male	No	Not Expose	0	0	0	3000	Bachelor	Bachelor	Orthodox	Georgian	City/Town
Low Risk	1	16	Male	No	Not Expose	0	0	0	2000	Bachelor	Master or	Orthodox	Georgian	City/Town
Medium Ri	3	24	Male	Yes	Exposure B	30	30	30	6000	Bachelor	Bachelor	Orthodox	Georgian	City/Town
Low Risk														
Low Risk	0	16	Female	Yes	Exposure B	90	180	180	3000	Master or	Bachelor	Orthodox	Georgian	Village
Low Risk	0	30	Female	Yes	Exposure B	30	30	30	22000	Master or	Master or	Orthodox	Georgian	City/Town
Low Risk	0	16	Female	No	Not Expose	1	10	20	8000	Bachelor	High Schoo	Orthodox	Georgian	City/Town
Medium Ri	6	30	Male	Yes	Exposure B	60	60	30	2500	PhD	PhD	Orthodox	Georgian	City/Town
Low Risk	1	27	Male	No	Not Expose	0	0	0	3000	Bachelor	Bachelor	Orthodox	Azerbaijan	City/Town
Low Risk	0	24	Male	No	Not Expose	10	90	90	2000	Bachelor	Bachelor	Orthodox	Georgian	City/Town
Low Risk	0	17	Female	No	Not Expose	0	0	0	7000	Bachelor	Bachelor	Orthodox	Georgian	City/Town
Low Risk	0	17	Male	No	Exposure B	20	15	20	1000	Bachelor	High Schoo	Orthodox	Georgian	Village
Low Risk	1	20	Female	Yes	Exposure B	180	180	90	3000	Bachelor	Bachelor	Orthodox	Georgian	City/Town
Low Risk	0	16	Male	Yes	Exposure B	5	15	30	5000	Bachelor	Bachelor	Orthodox	Georgian	Village
Low Risk	0	26	Female	No	Exposure B	15	100	150	1000	High Schoo	Middle Sch	Orthodox	Georgian	City/Town
Low Risk	0	23	Male	No	Exposure B	15	15	10	20000	Master or	Master or	Other	Mixed	City/Town
Low Risk	0	24	Male	No	Exposure B	120	120	120	10000	Bachelor	Bachelor	Orthodox	Mixed	City/Town
Low Risk	1	29	Female	No	Not Expose	30	30	30	500	Bachelor	Master or	Orthodox	Georgian	City/Town
Low Risk	0	22	Male	Yes	Exposure B	90	20	90	3000	Bachelor	Master or	Orthodox	Georgian	City/Town
Low Risk	0	30	Male	Yes	Exposure B	90	360	180	3000	Master or	Master or	Orthodox	Georgian	City/Town
Low Risk	1	16	Male	Yes	Exposure B	30	30	30	3000	High Schoo	High Schoo	Orthodox	Georgian	City/Town
Low Risk	0	16	Female	Yes	Exposure B	30	30	30	3000	Master or	Bachelor	Orthodox	Georgian	City/Town
Low Risk	0	23	Male	No	Exposure B	30	90	180	2000	Bachelor	High Schoo	Orthodox	Georgian	Village
Low Risk	2	16	Female	No	Not Expose	8	60	90	3000	Bachelor	Bachelor	Orthodox	Georgian	City/Town
Low Risk	2	16	Female	No	Not Expose	3	5	5	3000	Bachelor	High Schoo	Orthodox	Georgian	City/Town
Low Risk	1	21	Male	Yes	Exposure B	180	180	180	8000	High Schoo	Master or	Orthodox	Ukrainian	City/Town
Low Risk	0	16	Male	No	Not Expose	30	30	30	500	High Schoo	High Schoo	Orthodox	Georgian	City/Town
Medium Ri	5	30	Female	No	Exposure B	90	90	180	3000	High Schoo	High Schoo	Orthodox	Georgian	City/Town
Low Risk	1	16	Female	No	Exposure B	15	360	360	10000	Bachelor	Bachelor	Orthodox	Georgian	City/Town
Low Risk	0	17	Female	Yes	Exposure B	5	5	5	1500	Master or	High Schoo	Orthodox	Georgian	City/Town
Low Risk	2	24	Male	Yes	Exposure B	30	0	0	0	High Schoo	High Schoo	Orthodox	Georgian	Village
Low Risk	0	27	Male	No	Exposure B	90	180	180	1500	High Schoo	High Schoo	Orthodox	Georgian	City/Town
Low Risk	1	17	Male	Yes	Exposure B	30	30	60	3000	Master or	High Schoo	Orthodox	Georgian	City/Town
Low Risk	0	22	Male	No	Exposure B	15	15	50	3000	Bachelor	Bachelor	Orthodox	Georgian	City/Town
Low Risk	0	17	Female	No	Not Expose	10	10	1	2000	High Schoo	High Schoo	Orthodox	Georgian	Village
Low Risk	1	16	Male	No	Not Expose	0	0	0	10000	Bachelor	Middle Sch	Catholic	Mixed	Emigrant (West)
Low Risk	0	26	Male	Yes	Exposure B	30	180	180		Master or	Bachelor	Orthodox	Georgian	City/Town

Low Risk	2	21	Male	Yes	Exposure B	90	90	90	500	High Schoo	Master or I	Orthodox	Ukrainian	City/Town
Low Risk	0	26	Male	No	Exposure B	90	90	180	3000	PhD	Bachelor	Orthodox	Georgian	City/Town
Medium Ri	5	30	Female	Yes	Exposure B	90	180	180	1500	Bachelor	Bachelor	Orthodox	Georgian	Emigrant (West)
Low Risk	0	18	Female	No	Exposure B	30	90	90	2000	Bachelor	Bachelor	Orthodox	Georgian	City/Town
Medium Ri	6	30	Male	Yes	Exposure B	90	90	90	3000	Bachelor	Bachelor	Orthodox	Georgian	Mountainous Region
Low Risk	2	30	Male	No	Exposure B	180	360	180	1500	High Schoo	High Schoo	Orthodox	Georgian	City/Town
High Risk	11	30	Female	Yes	Exposure B	90	90	90	4000	Bachelor	High Schoo	Orthodox	Georgian	City/Town
Low Risk	0	17	Male	No	Exposure B	5	30	90	3000	Bachelor	Master or I	Orthodox	Georgian	City/Town
Low Risk	0	30	Male	No	Exposure B	60	90	90	3000	High Schoo	High Schoo	Orthodox	Georgian	Emigrant (West)
Low Risk	1	16	Female	No	Not Expose	15	15	29	1500	Master or I	Master or I	Orthodox	Georgian	Village
Low Risk	0	24	Male	Yes	Exposure B	90	90	180	3000	Master or I	PhD	Orthodox	Georgian	City/Town
Low Risk	1	30	Male	No	Exposure B	0	80	30	1200	Bachelor	High Schoo	Orthodox	Georgian	Village
Low Risk	0	22	Female	No	Exposure B	20	20	10	2000	Bachelor	High Schoo	Orthodox	Georgian	City/Town
Low Risk	0	23	Male	No	Exposure B	30	360	360	3000	Bachelor	Bachelor	Orthodox	Georgian	City/Town
Medium Ri	6	19	Female	Yes	Exposure B	90	180	360	1500	Bachelor	High Schoo	Orthodox	Georgian	City/Town
Low Risk	0	24	Female	Yes	Exposure B	30	30	30	5000	Master or I	Bachelor	Orthodox	Georgian	City/Town
Low Risk	0	22	Male	No	Exposure B	40	40	40	3000	Bachelor	High Schoo	Orthodox	Georgian	City/Town
Low Risk	1	16	Male	No	Not Expose	10	10	10	1500	Master or I	Bachelor	Orthodox	Georgian	City/Town
Medium Ri	4	21	Male	No	Exposure B	90	360	360	4000	Bachelor	Bachelor	Orthodox	Georgian	City/Town
Low Risk	0	18	Male	No	Not Expose	45	40	30	3000	Bachelor	High Schoo	Orthodox	Georgian	City/Town
Medium Ri	4	25	Female	Yes	Exposure B	20	30	30	6700	Bachelor	Bachelor	Orthodox	Georgian	City/Town
Low Risk	1	30	Female	Yes	Exposure B	30	30	30	2000	Bachelor	Bachelor	Orthodox	Georgian	City/Town
Low Risk	0	27	Male	No	Exposure B	60	90	90	400	Master or Equivalent		Orthodox	Georgian	City/Town
Low Risk	2	19	Male	Yes	Exposure B	90	90	30	4000	Master or I	High Schoo	Orthodox	Georgian	City/Town
High Risk	12	30	Female	Yes	Exposure B	30	180	180	1500	Bachelor	Bachelor	Orthodox	Georgian	Village
Low Risk	0	26	Female	Yes	Exposure B	90	90	90	3000	Bachelor	Bachelor	Orthodox	Georgian	City/Town
Low Risk	1	20	Male	No	Exposure B	30	360	360	1500	Bachelor	Middle Sch	Orthodox	Georgian	City/Town
Low Risk	0	16	Female	No	Not Expose	30	30	30	1500	Master or I	Bachelor	Orthodox	Russian	City/Town
Low Risk	0	24	Male	No	Not Expose	0	30	90	3000	High Schoo	High Schoo	Orthodox	Georgian	City/Town
Low Risk	2	29	Male	No	Exposure B	90	90	90	1500	High Schoo	High Schoo	Orthodox	Georgian	Village
Low Risk	0	16	Male	Yes	Exposure B	10	10	10	8000	Master or I	Master or I	Orthodox	Georgian	City/Town
Low Risk	1	29	Female	No	Exposure B	90	90	90	3000	Bachelor	Bachelor	Orthodox	Georgian	City/Town
Low Risk	0	30	Male	No	Exposure B	30			2500	Master or I	Master or I	Orthodox	Georgian	City/Town
Low Risk	0	24	Female	Yes	Exposure B	30	30	30	1500	Master or I	Bachelor	Orthodox	Georgian	City/Town
Low Risk	1	17	Male	No	Not Expose	5	30	60	1000	Bachelor	Bachelor	Orthodox	Georgian	Village
Low Risk	1	30	Female	No	Exposure Between 18 and 24 Months				1500	Bachelor	High Schoo	Orthodox	Georgian	Village
Low Risk	2	16	Male	No	Not Expose	30	30	30	3000	Bachelor	Bachelor	Orthodox	Georgian	City/Town
Low Risk	1	24	Male	Yes	Exposure B	30	30	30	3000	High Schoo	PhD	Muslim	Georgian	City/Town
Low Risk	1	18	Male	Yes	Exposure B	30	90	90	500	Bachelor	High Schoo	Orthodox	Georgian	Village
Medium Ri	3	18	Male	Yes	Exposure B	40	40	40	1800	Master or I	High Schoo	Orthodox	Georgian	City/Town
Low Risk	1	27	Female	Yes	Exposure B	30	30	60	4000	Bachelor	High Schoo	Orthodox	Georgian	City/Town
Low Risk	0	18	Male	No	Not Expose	5	5	5	12500	Master or I	Master or I	Orthodox	Georgian	City/Town
Medium Ri	7	18	Male	Yes	Exposure B	180	90	90	4000	Middle Sch	Bachelor	Orthodox	Armenian	City/Town
Low Risk	1	18	Male	Yes	Exposure B	90	180	180	3000	Bachelor	High Schoo	Orthodox	Georgian	Village

Low Risk	1	16	Female	No	Exposure B	30	0	0	3000	Bachelor	Master or	Orthodox	Georgian	City/Town
Low Risk	0	24	Female	No	Exposure B	60	15	75		Bachelor	Bachelor	Orthodox	Georgian	City/Town
Low Risk														
Low Risk	0	23	Female	Yes	Exposure B	90	600	800	6000	Bachelor	Middle Sch	Orthodox	Georgian	City/Town
Low Risk	2	30	Male	No	Exposure B	180	180	90	2000	Bachelor	Middle Sch	Orthodox	Georgian	City/Town
Low Risk	0	18	Female	No	Not Expose	90	90	0	2500	Bachelor	High Schoo	Orthodox	Georgian	Village
Medium Ri	4	21	Male	Yes	Exposure B	60	90	360	1700	High Schoo	High Schoo	Orthodox	Georgian	Village
Low Risk	0	21	Female	Yes	Exposure B	10	15	15	3500	Bachelor	Bachelor	Orthodox	Georgian	City/Town
Low Risk	2	30	Male	No	Exposure B	60	60	60	1500	Bachelor	High Schoo	Orthodox	Georgian	City/Town
Low Risk	2	30	Male	No	Exposure B	60	60	60	1500	Bachelor	Middle Sch	Orthodox	Mixed	City/Town
Low Risk	0	19	Male	Yes	Exposure B	30	90	18	7000	Master or	Bachelor	Orthodox	Georgian	City/Town
Low Risk	0	19	Male	Yes	Exposure Between 6 and 12 Months									
Low Risk	1	20	Male	Yes	Exposure B	120	180	30	2000	Bachelor	Bachelor	Orthodox	Georgian	City/Town
Low Risk	0	21	Female	Yes	Exposure B	30	30	20	10000	Bachelor	Master or	Orthodox	Georgian	City/Town
Low Risk	0	26	Female	No	Exposure B	60	120	180	2500	Bachelor	Bachelor	Orthodox	Georgian	City/Town
Low Risk	1	30	Male	Yes	Exposure B	180	180	360	1000	Bachelor	Bachelor	Orthodox	Georgian	City/Town
Low Risk	0	18	Female	Yes	Exposure B	90	60	30	5000	Master or	Bachelor	Orthodox	Georgian	City/Town
Low Risk	0	16	Male	No	Not Expose	13	60	60	6000	High Schoo	Bachelor	Orthodox	Georgian	City/Town
Low Risk	0	16	Female	No	Not Expose	0	0	0	5000	Bachelor	Bachelor	Orthodox	Georgian	City/Town
High Risk	13	16	Male	Yes	Exposure B	30	30	180	3000	Bachelor	High Schoo	Catholic	Georgian	Village
Low Risk	0	16	Male	Yes	Exposure B	30	90	30	3000	Bachelor	High Schoo	Catholic	Georgian	Village
Low Risk	1	21	Female	Yes	Exposure B	15	30	90	3500	High Schoo	High Schoo	Orthodox	Georgian	City/Town
Low Risk	1	16	Male	No	Not Expose	0	0	0		Master or	Master or	Other	Mixed	City/Town
Low Risk	0	19	Female	Yes	Exposure B	120	120	120	5000	Bachelor	Master or	Orthodox	Georgian	City/Town
Low Risk	1	19	Male	No	Not Expose	2	30	60	5600	Master or	Bachelor	Orthodox	Georgian	City/Town
Low Risk	1	18	Male	Yes	Exposure B	20	20	30	2000	High Schoo	High Schoo	Orthodox	Georgian	City/Town
Low Risk	0	24	Female	No	Not Expose	5	60	90	7000	Bachelor	Master or	Orthodox	Georgian	City/Town
Low Risk	1	24	Female	Yes	Exposure B	60	60	60	3000	High Schoo	High Schoo	Orthodox	Georgian	City/Town
Low Risk	0	17	Female	Yes	Exposure B	90	360	180	3000	Bachelor	Bachelor	Orthodox	Georgian	City/Town
Low Risk	0	26	Male	Yes	Exposure B	90	90	90	3000	Master or	Master or	Orthodox	Georgian	Village
Low Risk	0	24	Male	No	Exposure B	120	120	120	2500	Bachelor	Bachelor	Orthodox	Georgian	City/Town
Low Risk	0	18	Male	Yes	Exposure B	60	60	60	3000	Bachelor	High Schoo	Orthodox	Georgian	City/Town
Low Risk	0	24	Male	No	Exposure B	45	60	0	3000	Bachelor	Bachelor	Orthodox	Georgian	City/Town
Low Risk	1	16	Female	No	Exposure B	30	90	180	2000	Bachelor	High Schoo	Muslim	Georgian	City/Town
Low Risk	2	16	Female	Yes	Exposure B	30	30	360	2000	Bachelor	High Schoo	Muslim	Georgian	City/Town
Low Risk	1	30	Female	Yes	Exposure B	30	30	90	1500	High Schoo	High Schoo	Muslim	Georgian	City/Town
Medium Ri	6	26	Male	No	Exposure B	0	100	150	7000	Master or	Middle Sch	Orthodox	Georgian	Emigrant (West)
Low Risk	2	24	Female	No	Exposure B	15	15	7	1500	Bachelor	Master or	Orthodox	Georgian	City/Town
Medium Ri	3	19	Female	No	Exposure B	10	10	5	1000	Bachelor	Master or	Orthodox	Georgian	City/Town
Low Risk	1	30	Male	No	Exposure B	180	180	180	1200	Bachelor	Bachelor	Orthodox	Georgian	City/Town
Low Risk	1	18	Male	Yes	Exposure B	30	0	30	2500	Bachelor	Bachelor	Orthodox	Georgian	City/Town
Low Risk	0	25	Female	No	Exposure B	30	30	30	150	Bachelor	Bachelor	Orthodox	Georgian	Emigrant (West)
Medium Ri	6	24	Female	Yes	Exposure B	30	30	90	1500	High Schoo	High Schoo	Orthodox	Georgian	City/Town
Low Risk	0	30	Female	No	Exposure B	90	90	30	3000	High Schoo	High Schoo	Orthodox	Georgian	Village

Low Risk	0	29	Female	No	Exposure B	10	30	30	1500	High Schoo	High Schoo	Atheist	Georgian	Village
Low Risk	1	21	Male	Yes	Exposure B	180	180	180	100000	Bachelor	Bachelor	Orthodox	Mixed	Emigrant (West)
Medium Ri	7	21	Female	Yes	Exposure B	5	90	90	500	High Schoo	Bachelor	Orthodox	Georgian	Village
Medium Ri	6	24	Male	Yes	Exposure B	5	15	60	500	Bachelor	High Schoo	Orthodox	Georgian	City/Town
Low Risk	0	30	Male	No	Exposure B	90	30	90	1500	High Schoo	High Schoo	Muslim	Georgian	City/Town
Low Risk	0	24	Male	No	Exposure B	40	180	200	5500	Bachelor	Bachelor	Orthodox	Georgian	City/Town
High Risk	8	18	Male	Yes	Exposure B	90	90	90	1500	High Schoo	High Schoo	Orthodox	Georgian	Village
Medium Ri	7	18	Male	Yes	Exposure B	30	90	90	1500	High Schoo	High Schoo	Orthodox	Georgian	Village
Low Risk	0	16	Male	No	Not Expose	0	0	0	3000	High Schoo	Master or I	Muslim	Azerbaijan	City/Town
Low Risk	0	16	Male	Yes	Exposure B	8	300	300	3000	High Schoo	High Schoo	Orthodox	Georgian	City/Town
Low Risk	2	20	Male	Yes	Exposure B	30	30	60	5000	Master or I	Master or I	Orthodox	Georgian	City/Town
Low Risk	0	18	Female	Yes	Exposure B	90	90	90	4000	Bachelor	Middle Sch	Orthodox	Georgian	City/Town
Medium Ri	4	21	Male	Yes	Exposure B	90	180	360	1500	High Schoo	High Schoo	Orthodox	Georgian	City/Town
Medium Ri	3	30	Male	Yes	Exposure B	180	90	90	1500	Bachelor	Master or I	Orthodox	Georgian	Emigrant (West)
Medium Ri	3	25	Female	No	Not Expose	30	30	30	3000	Bachelor	High Schoo	Orthodox	Georgian	City/Town
Low Risk	1	22	Female	No	Exposure B	90	90	30	1500	Bachelor	Bachelor	Orthodox	Georgian	City/Town
Low Risk	0	16	Female	No	Not Expose	5	5	10	6000	Bachelor	Bachelor	Orthodox	Georgian	City/Town
Low Risk	0	16	Male	Yes	Exposure B	15	180	360	3000	High Schoo	High Schoo	Muslim	Azerbaijan	City/Town
Low Risk	1	24	Female	No	Exposure B	30	30	30	1000	Bachelor	Master or I	Orthodox	Russian	City/Town
Low Risk	1	24	Male	No	Exposure B	10	150	200	2000	Bachelor	Bachelor	Orthodox	Georgian	City/Town
Low Risk	1	30	Male	No	Exposure B	30	40	45	4000	Master or I	Bachelor	Orthodox	Georgian	City/Town
Low Risk	1	30	Male	Yes	Exposure B	30	30	30	2000	Bachelor	Bachelor	Orthodox	Georgian	City/Town
Low Risk	2	16	Female	No	Not Expose	30	30	30	8000	Master or I	Master or I	Orthodox	Georgian	City/Town
Low Risk	0	25	Female	Yes	Exposure B	180	180	420	2000	Bachelor	Bachelor	Orthodox	Georgian	City/Town
Medium Ri	5	30	Male	No	Exposure B	0	0	240	4000	Master or I	Master or I	Orthodox	Georgian	City/Town
Low Risk	0	16	Male	Yes	Exposure B	90	180	18	3000	Bachelor	Bachelor	Orthodox	Georgian	City/Town
High Risk	10	16	Male	Yes	Exposure B	25	35	13	500	Middle Sch	High Schoo	Orthodox	Georgian	Emigrant (West)
Low Risk	1	24	Male	No	Exposure B	30	180	180	3000	Bachelor	Bachelor	Orthodox	Georgian	City/Town
Medium Ri	4	21	Male	Yes	Exposure B	90	90	90	1500	Bachelor	High Schoo	Orthodox	Georgian	City/Town
Low Risk	2	16	Male	Yes	Exposure B	30	180	360	700	Middle Sch	Middle Sch	Orthodox	Georgian	Village
Low Risk	1	18	Male	Yes	Exposure B	45	45	30	2000	Bachelor	High Schoo	Orthodox	Georgian	Village
Low Risk	0	28	Male	No	Exposure B	30	10	30	500	Bachelor	High Schoo	Orthodox	Georgian	Village
Low Risk	0	28	Female	Yes	Exposure B	90	90	180	8000	High Schoo	Middle Sch	Orthodox	Georgian	Emigrant (Asia/Russia/Africa)
Low Risk	0	24	Male	Yes	Exposure B	30	30	90	3000	Master or I	Master or I	Orthodox	Georgian	City/Town
Medium Ri	4	30	Male	Yes	Exposure B	90	30	30	3000	Bachelor	High Schoo	Orthodox	Georgian	Village
Low Risk	2	18	Male	No	Exposure B	15	400	700	5000	Bachelor	Bachelor	Orthodox	Georgian	City/Town
Low Risk	0	19	Female	Yes	Exposure B	30	180	360	3000	Bachelor	Bachelor	Orthodox	Georgian	City/Town
Low Risk	2	19	Female	No	Exposure B	30	30	30	2500	Bachelor	High Schoo	Orthodox	Georgian	City/Town
Low Risk	0	22	Female	No	Exposure B	130	90	60	3000	Bachelor	Bachelor	Orthodox	Georgian	City/Town
Medium Ri	3	17	Male	Yes	Exposure B	40	20	5	4000	Bachelor	Bachelor	Orthodox	Georgian	City/Town
Low Risk	0	24	Male	No	Exposure B	10	180	180	6000	Bachelor	Master or I	Orthodox	Mixed	City/Town
Low Risk	1	24	Male	No	Exposure B	90	180	180	3000	Master or I	Bachelor	Orthodox	Georgian	City/Town
Medium Ri	3	16	Male	Yes	Exposure B	15	15	20	2000	Bachelor	High Schoo	Muslim	Georgian	Village
Low Risk	0	19	Female	Yes	Exposure B	15	360	360	4000	Bachelor	Bachelor	Orthodox	Georgian	Village

Low Risk														
Low Risk	1	16	Male	No	Not Expose	5	5	5	3000	Bachelor	Bachelor	Orthodox	Georgian	Village
Low Risk	1	18	Female	No	Not Expose	0	0	0	2000	Master or I	Bachelor	Orthodox	Georgian	City/Town
Low Risk	0	30	Male	No	Exposure B	90	90	360	150	Bachelor	High Schoo	Orthodox	Georgian	City/Town
High Risk	9	18	Male	Yes	Exposure B	50	250	300	5000	Bachelor	Bachelor	Orthodox	Georgian	City/Town
Low Risk	0	30	Female	No	Exposure B	30	30	90	1500	Bachelor	Bachelor	Orthodox	Georgian	City/Town
Low Risk	0	23	Male	Yes	Exposure B	30	30	15	5000	Bachelor	Master or I	Orthodox	Georgian	City/Town
Medium Ri	5	16	Male	Yes	Exposure B	35	40	30	1500	Middle Sch	High Schoo	Orthodox	Georgian	Emigrant (West)
Low Risk	1	20	Male	No	Exposure B	10	30	90	4000	Master or I	High Schoo	Orthodox	Mixed	City/Town
Low Risk	1	18	Male	No	Not Expose	5	90	90	4000	Master or I	High Schoo	Orthodox	Mixed	City/Town
Low Risk	0	24	Male	No	Exposure B	30	60	60	4000	Master or I	PhD	Orthodox	Georgian	City/Town
Medium Ri	5	18	Male	Yes	Exposure B	10	150	150	6000	Master or I	Master or I	Orthodox	Georgian	City/Town
Low Risk	2	18	Female	No	Exposure B	15	10	0	3000	Bachelor	Bachelor	Orthodox	Georgian	City/Town
Low Risk	0	18	Female	Yes	Exposure B	30	30	30		Bachelor	Bachelor	Orthodox	Georgian	City/Town
Low Risk	1	18	Male	Yes	Exposure B	20	15	20	3000	Bachelor	Bachelor	Orthodox	Georgian	City/Town
Low Risk	2	25	Male	Yes	Exposure B	10	10	90	4000	Bachelor	Bachelor	Orthodox	Georgian	City/Town
Medium Ri	5	20	Male	Yes	Exposure B	90	90	90	1500	High Schoo	Bachelor	Orthodox	Mixed	City/Town
Medium Ri	6	16	Male	Yes	Exposure B	90	360	480	3000	Bachelor	Bachelor	Orthodox	Georgian	City/Town
Low Risk	0	24	Male	Yes	Exposure B	30	140	180	2000	Bachelor	PhD	Muslim	Georgian	City/Town
Low Risk	0	24	Male	Yes	Exposure B	30	120	180	2000	Bachelor	PhD	Muslim	Georgian	City/Town
Medium Ri	3	24	Male	Yes	Exposure B	180	180	180	4000	Middle Sch	Middle Sch	Orthodox	Georgian	Emigrant (West)
Low Risk	1	16	Female	Yes	Exposure B	30	90	360	500	High Schoo	High Schoo	Orthodox	Georgian	City/Town
Medium Ri	3	19	Male	No	Exposure B	10	50	90	3000	Bachelor	Bachelor	Orthodox	Georgian	City/Town
Low Risk	1	17	Female	No	Not Expose	20	180	180	5000	Bachelor	Bachelor	Orthodox	Georgian	City/Town
Low Risk	0	20	Female	No	Exposure B	360	360	360	1500	High Schoo	High Schoo	Orthodox	Georgian	City/Town
Low Risk	0	16	Female	Yes	Exposure B	20	20	20	3500	Master or I	Master or I	Orthodox	Georgian	City/Town
Low Risk	0	16	Male	Yes	Exposure B	0	30	90	1500	Bachelor	Bachelor	Orthodox	Georgian	City/Town
Low Risk	0	20	Female	Yes	Exposure B	30	60	60	5000	Master or I	Bachelor	Orthodox	Georgian	City/Town
Low Risk	1	17	Male	Yes	Exposure B	20	18	18	1500	Bachelor	High Schoo	Orthodox	Georgian	City/Town
Low Risk	0	17	Male	Yes	Exposure B	60	360	500	3000	Master or I	Master or I	Orthodox	Georgian	City/Town
Low Risk	0	25	Male	Yes	Exposure Between 6 and 12 Months									
Medium Ri	6	24	Male	No	Exposure B	360	180	100	7000	High Schoo	Middle Sch	Orthodox	Georgian	Emigrant (West)
Medium Ri	5	16	Male	Yes	Exposure B	0	360	360	4000	High Schoo	High Schoo	Orthodox	Georgian	Emigrant (West)
Medium Ri	6	16	Male	Yes	Exposure B	180	100	60	8000	High Schoo	High Schoo	Muslim	Georgian	Emigrant (West)
High Risk	8	24	Male	Yes	Exposure B	540	400	300		High Schoo	Middle Sch	Orthodox	Georgian	Emigrant (West)
Medium Ri	5	18	Male	Yes	Exposure B	30	30	30	3000	Bachelor	Bachelor	Orthodox	Georgian	City/Town
Medium Ri	3	16	Male	Yes	Exposure B	60	60	90	1000	High Schoo	High Schoo	Orthodox	Georgian	Village
Low Risk														
Medium Ri	3	24	Female	Yes	Exposure B	90	180	360	2000	Bachelor	Bachelor	Orthodox	Georgian	Emigrant (West)
Medium Ri	6	28	Male	No	Not Expose	0	0	0	2000	Bachelor	High Schoo	Orthodox	Georgian	Village
Low Risk	0	24	Female	Yes	Exposure B	180	360	400	3500	Bachelor	Bachelor	Orthodox	Georgian	City/Town
Low Risk	1	18	Female	Yes	Exposure B	30	30	30	1500	High Schoo	High Schoo	Orthodox	Mixed	City/Town
Medium Ri	3	16	Male	Yes	Exposure B	90	30	30	2000	High Schoo	Master or I	Orthodox	Georgian	City/Town
Low Risk	2	16	Female	No	Not Expose	30	30	30	1000	High Schoo	High Schoo	Orthodox	Georgian	City/Town

Medium Ri	3	30	Male	Yes	Exposure B	30	90	360	150	High Schoo	Bachelor	Orthodox	Georgian	City/Town
Low Risk	1	16	Male	Yes	Exposure B	30	30	30	4000	Bachelor	Bachelor	Orthodox	Mixed	Village
Low Risk	0	16	Male	Yes	Exposure B	15	15	15	1900	Bachelor	High Schoo	Orthodox	Georgian	Village
Medium Ri	7	17	Male	Yes	Exposure B	40	180	180	5000	Bachelor	High Schoo	Orthodox	Georgian	City/Town
Medium Ri	3	23	Male	No	Exposure B	0	90	60	5000	Bachelor	Bachelor	Orthodox	Georgian	Emigrant (West)
Low Risk	0	24	Male	No	Exposure B	90	180	360	500	High Schoo	Middle Sch	Orthodox	Azerbaijan	City/Town
Low Risk	0	16	Male	Yes	Exposure B	30	30	30	1000	High Schoo	High Schoo	Orthodox	Georgian	City/Town
Low Risk	0	19	Male	No	Exposure B	20	200	20	2000	Bachelor	High Schoo	Orthodox	Georgian	Village
Low Risk	0	22	Male	No	Not Expose	10	10	10		Master or I	Bachelor	Orthodox	Georgian	City/Town
Low Risk	1	16	Male	Yes	Exposure B	10			2000	Master or I	Bachelor	Orthodox	Georgian	Village
Low Risk	1	16	Male	No	Exposure B	120			3000	Middle Sch	High Schoo	Orthodox	Georgian	Village
Low Risk	0	21	Male	No	Exposure B	130			3000	Bachelor	Bachelor	Orthodox	Georgian	City/Town
Low Risk	0	24	Male	Yes	Exposure B	60			5000	Bachelor	High Schoo	Orthodox	Mixed	City/Town
Low Risk	0	18	Male	Yes	Exposure B	90			3500	Master or I	Bachelor	Orthodox	Georgian	City/Town
Low Risk	2	20	Male	Yes	Exposure B	60			2500	Bachelor	Bachelor	Orthodox	Georgian	City/Town
Low Risk	0	16	Male	Yes	Exposure B	30			1000	High Schoo	High Schoo	Orthodox	Georgian	City/Town
Medium Ri	3	20	Male	Yes	Exposure B	180			1500	Bachelor	High Schoo	Orthodox	Georgian	City/Town
Low Risk	1	30	Male	No	Exposure B	90			2000	Master or I	High Schoo	Orthodox	Georgian	City/Town
Medium Ri	3	26	Female	No	Not Expose	30			3000	High Schoo	High Schoo	Orthodox	Georgian	Village
Low Risk	0	24	Male	Yes	Exposure B	90			1500	High Schoo	High Schoo	Orthodox	Georgian	City/Town
Low Risk	2	24	Male	Yes	Exposure B	300			1000	High Schoo	High Schoo	Orthodox	Georgian	Village
Low Risk	1	20	Male	No	Exposure B	15			3000	High Schoo	High Schoo	Orthodox	Georgian	City/Town
Low Risk	1	20	Male	No	Exposure B	15			3000	High Schoo	High Schoo	Orthodox	Georgian	City/Town
Medium Ri	4	17	Male	Yes	Exposure B	90			7000	Bachelor	Master or I	Orthodox	Georgian	City/Town
Low Risk	0	16	Female	No	Not Expose	5			1500	Bachelor	Bachelor	Orthodox	Georgian	City/Town
Low Risk	0	16	Male	Yes	Exposure B	180			3000	Bachelor	Middle Sch	Orthodox	Georgian	City/Town
Low Risk	1	18	Female	No	Exposure B	10			5000	Bachelor	Bachelor	Orthodox	Georgian	City/Town
High Risk	9	16	Male	Yes	Exposure B	180			500	High Schoo	High Schoo	Orthodox	Georgian	City/Town
Medium Ri	5	16	Male	Yes	Exposure B	30			1500	High Schoo	High Schoo	Orthodox	Georgian	City/Town
Medium Ri	3	23	Male	Yes	Exposure B	30			3000	Bachelor	Bachelor	Orthodox	Georgian	City/Town
Low Risk	0	30	Male	No	Exposure B	180			3000	High Schoo	High Schoo	Muslim	Mixed	Emigrant (West)
Low Risk	0	16	Female	No	Exposure B	30			6000	Master or I	PhD	Orthodox	Georgian	City/Town
Low Risk	0	27	Female	No	Exposure B	30			3000	Master or I	PhD	Orthodox	Georgian	Village
Low Risk	1	30	Male	No	Exposure B	90			3000	Master or I	Bachelor	Orthodox	Georgian	City/Town
Low Risk	2	20	Male	Yes	Exposure B	90			500	Bachelor	Bachelor	Orthodox	Georgian	City/Town
Low Risk	1	30	Male	Yes	Exposure B	90			500	Bachelor	Bachelor	Orthodox	Georgian	City/Town
Low Risk	2	16	Male	Yes	Exposure B	15			1900	Bachelor	High Schoo	Orthodox	Georgian	Village
High Risk	8	24	Male	Yes	Exposure B	30			2000	High Schoo	High Schoo	Orthodox	Georgian	City/Town
Medium Ri	7	16	Male	Yes	Exposure B	360			4500	High Schoo	High Schoo	Orthodox	Georgian	Emigrant (West)
High Risk	8	16	Male	Yes	Exposure B	180			4500	High Schoo	High Schoo	Orthodox	Georgian	Emigrant (West)
Low Risk	0	30	Male	No	Exposure B	30			500	Bachelor	High Schoo	Orthodox	Georgian	City/Town
Low Risk	0	18	Male	Yes	Exposure B	90			1500	Bachelor	Bachelor	Orthodox	Georgian	City/Town
Low Risk	1	20	Male	Yes	Exposure B	30			4000	Master or I	Bachelor	Orthodox	Georgian	City/Town
Low Risk	0	30	Female	No	Exposure B	180			2000	Bachelor	Master or I	Orthodox	Georgian	City/Town

Medium Ri	3	24	Male	Yes	Exposure B	180			5000	Master or I	Bachelor	Orthodox	Georgian	City/Town
Low Risk	1	18	Female	Yes	Exposure B	30			2500	High Schoo	High Schoo	Orthodox	Georgian	City/Town
High Risk	14	30	Male	Yes	Exposure B	360			5000	Bachelor	Bachelor	Orthodox	Georgian	City/Town
Medium Ri	6	16	Male	Yes	Exposure B	15			1900	Bachelor	High Schoo	Orthodox	Georgian	Village
High Risk	8	20	Male	Yes	Exposure B	90			1500	High Schoo	High Schoo	Orthodox	Georgian	Village
Low Risk	1	30	Male	Yes	Exposure B	90			3000	Master or I	Master or I	Other	Georgian	City/Town
Low Risk	2	16	Male	No	Not Expose	10			1500	Bachelor	Bachelor	Orthodox	Georgian	City/Town
Low Risk	0	19	Female	Yes	Exposure B	90			4000	Bachelor	High Schoo	Orthodox	Georgian	Village
Medium Ri	7	30	Female	Yes	Exposure B	0			1500	Bachelor	Bachelor	Orthodox	Georgian	Emigrant (West)
Medium Ri	4	23	Male	Yes	Exposure B	10			4000	Bachelor	Bachelor	Orthodox	Georgian	City/Town
Medium Ri	4	22	Male	No	Exposure B	20			3000	Master or I	High Schoo	Orthodox	Georgian	City/Town
Low Risk	0	16	Male	No	Exposure B	8				Bachelor	Bachelor	Orthodox	Georgian	City/Town
Low Risk	2	30	Female	Yes	Exposure B	60			1500	Bachelor	Bachelor	Orthodox	Georgian	City/Town
Low Risk	2	16	Male	No	Not Expose	0			1500	Bachelor	Bachelor	Orthodox	Georgian	Emigrant (West)
Low Risk	1	18	Male	Yes	Exposure B	17			1500	High Schoo	High Schoo	Orthodox	Georgian	City/Town
Low Risk	0	24	Female	No	Exposure B	30			1500	Middle Sch	Middle Sch	Orthodox	Georgian	City/Town
Medium Ri	4	16	Male	Yes	Exposure B	15			1500	Bachelor	High Schoo	Orthodox	Georgian	Village
Medium Ri	3	16	Male	No	Exposure B	15			1500	Bachelor	High Schoo	Orthodox	Georgian	Village
Low Risk	0	19	Female	Yes	Exposure B	30			3000	Master or I	Bachelor	Orthodox	Georgian	City/Town
Low Risk	0	16	Female	Yes	Exposure B	15			3000	Bachelor	Master or I	Orthodox	Georgian	Mountainous Region
Medium Ri	4	16	Female	Yes	Exposure B	15			5000	Bachelor	High Schoo	Orthodox	Georgian	City/Town
Low Risk	0	24	Male	No	Exposure B	15			3000	High Schoo	High Schoo	Muslim	Georgian	City/Town
Medium Ri	4	23	Male	No	Exposure B	0			5000	Bachelor	Master or I	Orthodox	Georgian	City/Town
Medium Ri	4	21	Male	No	Exposure B	0			5000	Bachelor	Bachelor	Orthodox	Georgian	City/Town
Low Risk	0	30	Female	Yes	Exposure B	90			1500	Bachelor	Bachelor	Orthodox	Georgian	City/Town
Low Risk														
Low Risk	1	24	Male	Yes	Exposure B	30			3000	High Schoo	PhD	Orthodox	Georgian	City/Town
Low Risk	0	24	Male	Yes	Exposure B	30			3000	High Schoo	PhD	Orthodox	Georgian	City/Town
Low Risk	1	30	Female	No	Not Expose	30			1500	High Schoo	High Schoo	Orthodox	Georgian	City/Town
Low Risk	2	20	Male	Yes	Exposure B	90			1500	Master or I	High Schoo	Orthodox	Georgian	City/Town
Low Risk	1	16	Male	Yes	Exposure B	5			2500	Master or I	Bachelor	Orthodox	Georgian	City/Town
Medium Ri	7	16	Female	Yes	Exposure B	22			2500	Bachelor	Bachelor	Orthodox	Georgian	City/Town
Low Risk	1	16	Male	Yes	Exposure B	20			3000	Bachelor	Bachelor	Orthodox	Georgian	City/Town
Low Risk	0	22	Female	Yes	Exposure B	30			2300	Bachelor	High Schoo	Orthodox	Georgian	City/Town
Low Risk	1	20	Female	Yes	Exposure B	30			1000	Bachelor	Bachelor	Orthodox	Georgian	Emigrant (West)
Low Risk	0	16	Female	Yes	Exposure B	90			1000	Bachelor	Bachelor	Orthodox	Georgian	City/Town
Low Risk	1	16	Male	Yes	Exposure B	30			1400	High Schoo	High Schoo	Orthodox	Georgian	Village
High Risk	8	26	Male	No	Exposure B	90			3000	Bachelor	Bachelor	Orthodox	Georgian	Village
Low Risk	0	27	Female	Yes	Exposure B	30			1500	Bachelor	Bachelor	Orthodox	Georgian	Village
Low Risk	2	17	Female	Yes	Exposure B	90			1500	High Schoo	Bachelor	Orthodox	Georgian	City/Town
Low Risk	1	23	Male	Yes	Exposure B	180			3000	High Schoo	Bachelor	Orthodox	Mixed	City/Town
Low Risk	0	16	Female	Yes	Exposure B	30			5000	Bachelor	Middle Sch	Orthodox	Georgian	City/Town
Low Risk														
Low Risk	0	16	Male	Yes	Exposure B	10			2500	Bachelor	High Schoo	Orthodox	Georgian	City/Town

Low Risk	1	25	Female	Yes	Exposure B	30			10000	High Schoo	Bachelor	Orthodox	Georgian	City/Town
Low Risk	0	30	Female	No	Exposure B	180			7000	Master or	Master or	Orthodox	Georgian	City/Town
Low Risk	2	24	Male	Yes	Exposure B	60			1500	High Schoo	High Schoo	Orthodox	Georgian	City/Town
Low Risk	0	16	Female	Yes	Exposure B	30				Master or	Master or	Orthodox	Georgian	City/Town
Medium Ri	6	23	Male	No	Exposure B	90			2150	High Schoo	High Schoo	Orthodox	Georgian	City/Town
Low Risk	2	18	Male	Yes	Exposure B	90			2000	High Schoo	Bachelor	Orthodox	Georgian	City/Town
Low Risk	1	17	Female	Yes	Exposure B	60			4000	High Schoo	Bachelor	Orthodox	Georgian	City/Town
Low Risk	0	18	Male	No	Exposure B	30			3000	Bachelor	High Schoo	Orthodox	Georgian	City/Town
Low Risk	0	30	Male	Yes	Exposure B	90			2500	Bachelor	Master or	Orthodox	Georgian	City/Town
Low Risk	1	20	Female	Yes	Exposure B	20			1200	Bachelor	High Schoo	Orthodox	Georgian	City/Town
Medium Ri	4	16	Male	Yes	Exposure B	30			3000	High Schoo	Master or	Atheist	Georgian	City/Town
Medium Ri	3	24	Male	No	Exposure B	90			1000	Bachelor	High Schoo	Orthodox	Georgian	City/Town
Low Risk	2	16	Female	No	Not Expose	10			3000	Bachelor	Bachelor	Orthodox	Georgian	City/Town
Low Risk	0	18	Male	No	Exposure B	20			6000	Master or	High Schoo	Orthodox	Georgian	City/Town
Low Risk	0	27	Male	Yes	Exposure B	180			1500	Bachelor	High Schoo	Orthodox	Georgian	City/Town
Medium Ri	3	16	Female	Yes	Exposure B	30			4500	Bachelor	Middle Sch	Orthodox	Georgian	City/Town
Low Risk	0	25	Male	Yes	Exposure B	90			3000	High Schoo	High Schoo	Orthodox		
Low Risk	2	30	Male	Yes	Exposure B	45			600	High Schoo	High Schoo	Orthodox	Georgian	City/Town
Low Risk	0	30	Male	Yes	Exposure B	90			2000	Bachelor	High Schoo	Muslim	Georgian	Village
Medium Ri	7	21	Male	No	Exposure B	30			1500	Middle Sch	Middle Sch	Orthodox	Georgian	City/Town
Medium Ri	4	17	Female	Yes	Exposure B	90			1100	High Schoo	High Schoo	Orthodox	Georgian	City/Town
Medium Ri	7	24	Female	Yes	Exposure B	90				Middle Sch	High Schoo	Orthodox	Georgian	Emigrant (West)
Low Risk	1	25	Male	No	Exposure B	60			5000	Master or	Master or	Orthodox	Georgian	Emigrant (West)
Medium Ri	3	30	Female	Yes	Exposure B	90			3000	Bachelor	Bachelor	Orthodox	Georgian	City/Town
Low Risk	0	30	Female	No	Exposure B	60			10000	Master or	Master or	Orthodox	Georgian	City/Town
Low Risk	0	30	Male	No	Exposure B	30			2500	Master or	High Schoo	Orthodox	Georgian	City/Town
Low Risk	2	27	Female	Yes	Exposure B	3			3000	High Schoo	Bachelor	Orthodox	Georgian	City/Town
Low Risk	0	23	Female	No	Exposure B	60			1700	Bachelor	High Schoo	Orthodox	Georgian	City/Town
Low Risk	1	30	Female	Yes	Exposure B	90			1500	Master or	Master or	Orthodox	Georgian	City/Town
Low Risk	1	22	Male	Yes	Exposure B	90			3000	Bachelor	High Schoo	Orthodox	Georgian	City/Town
Low Risk	1	22	Male	Yes	Exposure B	90			3000	Bachelor	High Schoo	Orthodox	Georgian	City/Town
Low Risk	1	19	Male	Yes	Exposure B	40			3000	High Schoo	High Schoo	Orthodox	Georgian	City/Town
Medium Ri	7	30	Male	Yes	Exposure B	180			1500	Master or	Master or	Orthodox	Georgian	Mountainous Region
Low Risk	1	30	Male	No	Exposure B	60			10000	Bachelor	Bachelor	Orthodox	Georgian	City/Town
Low Risk	0	30	Female	No	Exposure B	90			5000	Bachelor	Bachelor	Orthodox	Georgian	City/Town
Low Risk	0	30	Male	No	Exposure B	30			1500	Master or	Bachelor	Orthodox	Georgian	City/Town
Low Risk	0	30	Male	Yes	Exposure B	30			500	High Schoo	High Schoo	Orthodox	Georgian	Village
Low Risk	0	29	Male	No	Exposure B	45			2000	Bachelor	Bachelor	Orthodox	Georgian	City/Town
Low Risk	1	16	Male	Yes	Exposure B	30			2500	High Schoo	Bachelor	Orthodox	Georgian	City/Town
Low Risk	0	16	Male	No	Exposure B	30			4000	Bachelor	High Schoo	Orthodox	Georgian	City/Town
Low Risk	0	16	Male	No	Not Expose	30			3000	High Schoo	High Schoo	Orthodox	Georgian	City/Town
Low Risk	0	23	Male	No	Exposure B	30			1000	High Schoo	High Schoo	Orthodox	Georgian	City/Town
Low Risk	1	27	Male	No	Exposure B	90			2500	Bachelor	Bachelor	Orthodox	Georgian	City/Town
Low Risk	0	30	Male	No	Exposure B	30			5500	Master or	Bachelor	Orthodox	Georgian	City/Town

Low Risk	0	16	Male	Yes	Exposure B	90			1500	High Schoo	High Schoo	Muslim	Georgian	City/Town
Low Risk	0	16	Male	No	Not Expose	0			1700	High Schoo	High Schoo	Orthodox	Georgian	City/Town
Low Risk	0	16	Male	No	Not Expose	0			3000	High Schoo	High Schoo	Orthodox	Georgian	City/Town
Low Risk	0	29	Male	Yes	Exposure B	60			4000	Bachelor	Bachelor	Orthodox	Georgian	City/Town
Low Risk	1	16	Male	No	Exposure B	20			50000	High Schoo	High Schoo	Orthodox	Georgian	Village
Medium Ri	3	16	Female	Yes	Exposure B	30			3000	High Schoo	High Schoo	Orthodox	Georgian	City/Town
Low Risk	0	30	Male	Yes	Exposure B	30			3000	Bachelor	Middle Sch	Orthodox	Georgian	City/Town
Low Risk	0	30	Male	No	Exposure B	30			8000	Master or I	PhD	Atheist	Georgian	Emigrant (West)
Low Risk	1	21	Female	No	Exposure B	90			2000	Bachelor	High Schoo	Orthodox	Georgian	City/Town
Low Risk	1	16	Male	Yes	Exposure B	15			7000	Master or I	Master or I	Orthodox	Georgian	City/Town
Low Risk	0	16	Female	Yes	Exposure B	22			700	Bachelor	High Schoo	Orthodox	Georgian	Village
Medium Ri	5	19	Male	Yes	Exposure B	240			2500	High Schoo	High Schoo	Orthodox	Georgian	City/Town
Low Risk	0	18	Male	No	Exposure B	90			10000	Master or I	Bachelor	Orthodox	Georgian	City/Town
Low Risk	1	16	Male	Yes	Exposure B	15			0	Bachelor	Bachelor	Orthodox	Georgian	City/Town
Low Risk	1	18	Female	No	Exposure B	10			8000	Master or I	Bachelor	Orthodox	Georgian	City/Town
Low Risk	1	17	Male	No	Exposure B	40			1000	High Schoo	High Schoo	Orthodox	Georgian	City/Town
Low Risk	0	22	Female	Yes	Exposure B	30			600	Bachelor	High Schoo	Orthodox	Georgian	City/Town
Low Risk	0	18	Male	Yes	Exposure B	20			4500	PhD	Master or I	Orthodox	Georgian	City/Town
Low Risk	1	16	Male	Yes	Exposure B	40			2000	High Schoo	High Schoo	Protestant	Georgian	Village
Low Risk	1	16	Male	No	Not Expose	10			6000	Master or I	PhD	Orthodox	Georgian	City/Town
Low Risk	1	30	Male	No	Exposure B	30			1000	High Schoo	High Schoo	Muslim	Georgian	City/Town
Low Risk	0	21	Female	No	Exposure B	30			2500	Master or I	High Schoo	Orthodox	Georgian	City/Town
Low Risk	0	16	Male	No	Not Expose	5			10000	Bachelor	Bachelor	Orthodox	Georgian	Emigrant (West)
Low Risk	1	25	Male	No	Exposure B	60			2500	Bachelor	High Schoo	Orthodox	Georgian	City/Town
Low Risk	0	21	Female	No	Exposure B	30			5000	Bachelor	Master or I	Orthodox	Georgian	City/Town
Low Risk	1	20	Male	No	Exposure B	30			3000	Bachelor	High Schoo	Orthodox	Georgian	City/Town
Low Risk	2	24	Male	Yes	Exposure B	60			3000	Bachelor	Bachelor	Orthodox	Georgian	City/Town
Medium Ri	4	26	Female	Yes	Exposure B	180			1500	Bachelor	Master or I	Orthodox	Georgian	City/Town
Low Risk	0	22	Female	Yes	Exposure B	30			3000	Master or I	Bachelor	Orthodox	Georgian	Emigrant (West)
Low Risk	2	16	Male	Yes	Exposure B	90			1200	High Schoo	Middle Sch	Orthodox	Georgian	City/Town
Low Risk	0	22	Male	No	Exposure B	30			3000	Master or I	Bachelor	Orthodox	Georgian	City/Town
Low Risk	0	17	Female	Yes	Exposure B	60			4000	Bachelor	Bachelor	Muslim	Other	City/Town
Low Risk	0	26	Male	No	Exposure B	30			1500	Bachelor	Bachelor	Orthodox	Georgian	City/Town
Low Risk	1	30	Male	Yes	Exposure B	180			1500	Bachelor	Bachelor	Orthodox	Georgian	Village
Low Risk	0	24	Male	Yes	Exposure B	90			4000	Master or I	High Schoo	Orthodox	Georgian	City/Town
Low Risk	0	16	Female	No	Exposure B	60			1500	Bachelor	Bachelor	Muslim	Georgian	Village
Medium Ri	3	16	Female	No	Exposure B	10			1500	Bachelor	Bachelor	Orthodox	Georgian	City/Town
Low Risk	1	24	Male	No	Exposure B	360			1500	Bachelor	Bachelor	Orthodox	Georgian	City/Town
Low Risk	2	18	Female	Yes	Exposure B	120			2000	Bachelor	Bachelor	Orthodox	Georgian	City/Town
Low Risk	1	18	Male	Yes	Exposure B	180			5000	Middle Sch	Middle Sch	Orthodox	Georgian	Emigrant (West)
Low Risk	1	16	Male	Yes	Exposure B	20			3000	Bachelor	Bachelor	Orthodox	Georgian	City/Town
Low Risk	0	17	Male	No	Not Expose	0			3000	Bachelor	Bachelor	Orthodox	Georgian	City/Town
Low Risk	0	28	Male	No	Exposure B	30			3000	Bachelor	Bachelor	Orthodox	Georgian	City/Town
Low Risk	2	30	Male	No	Exposure B	45			7000	Bachelor	Master or I	Orthodox	Georgian	City/Town

Low Risk	0	29	Female	No	Exposure B	90			4000	Bachelor	Bachelor	Orthodox	Georgian	City/Town
Medium Ri	3	21	Female	Yes	Exposure B	60			2000	Bachelor	Bachelor	Orthodox	Georgian	City/Town
Medium Ri	3	16	Female	Yes	Exposure B	15			1500	High Schoo	High Schoo	Orthodox	Georgian	City/Town
Medium Ri	3	16	Female	Yes	Exposure B	30			4000	High Schoo	Master or	Orthodox	Georgian	City/Town
Low Risk	1	21	Female	No	Exposure B	30			3000	Master or	Middle Sch	Orthodox	Georgian	Village
Low Risk	1	16	Female	Yes	Exposure B	60			7000	Bachelor	Bachelor	Orthodox	Georgian	Emigrant (West)
Low Risk	2	21	Male	No	Exposure B	90			6000	Master or	High Schoo	Orthodox	Georgian	City/Town
Low Risk	0	30	Female	Yes	Exposure B	90			2000	Bachelor	High Schoo	Orthodox	Georgian	City/Town
Low Risk	1	27	Male	No	Not Expose	20			3000	Master or	High Schoo	Muslim	Georgian	Mountainous Region
Medium Ri	6	19	Male	No	Not Expose	0			1500	Master or	Master or	Orthodox	Georgian	Village
Low Risk	2	16	Female	No	Not Expose	0			3000	High Schoo	High Schoo	Orthodox	Georgian	City/Town
Low Risk	2	16	Male	Yes	Exposure B	90			3000	Bachelor	Master or	Orthodox	Georgian	City/Town
Medium Ri	5	25	Male	No	Exposure B	360			1500	High Schoo	Master or	Orthodox	Georgian	City/Town
Medium Ri	3	17	Male	Yes	Exposure B	90			3000	Bachelor	Bachelor	Orthodox	Georgian	City/Town
Medium Ri	5	30	Male	Yes	Exposure B	360			4000	Master or	High Schoo	Orthodox	Georgian	City/Town
Low Risk	0	30	Female	No	Exposure B	180			2500	Master or	Master or	Orthodox	Georgian	Village
Low Risk	1	30	Female	No	Not Expose	360			500	High Schoo	High Schoo	Orthodox	Georgian	Emigrant (West)
Medium Ri	6	16	Male	Yes	Exposure B	30			6000	Bachelor	Bachelor	Orthodox	Georgian	City/Town
Low Risk	0	30	Male	Yes	Exposure B	90			2500	Middle Sch	High Schoo	Orthodox	Georgian	City/Town
Low Risk	0	20	Male	No	Exposure B	30			3000	Middle Sch	High Schoo	Orthodox	Georgian	City/Town
High Risk	13	20	Male	Yes	Exposure B	360				Middle Sch	High Schoo	Orthodox	Georgian	City/Town
Low Risk	0	26	Male	Yes	Exposure B	180			1500	Bachelor	Bachelor	Orthodox	Georgian	City/Town
Low Risk	0	16	Female	No	Not Expose	20			2000	Bachelor	Master or	Orthodox	Georgian	Village
Low Risk	1	18	Male	Yes	Exposure B	60			1000	Bachelor	High Schoo	Other	Georgian	City/Town
Low Risk	0	18	Male	Yes	Exposure B	30			1500	Bachelor	Bachelor	Orthodox	Georgian	City/Town
Low Risk	0	16	Male	No	Not Expose	10			2000	Bachelor	High Schoo	Orthodox	Georgian	City/Town
Medium Ri	6	21	Male	Yes	Exposure B	360			1000	Bachelor	Bachelor	Orthodox	Georgian	City/Town
Medium Ri	4	21	Male	Yes	Exposure B	360			1000	Bachelor	High Schoo	Orthodox	Georgian	City/Town
Medium Ri	6	16	Male	Yes	Exposure B	90			6000	Bachelor	Bachelor	Orthodox	Georgian	City/Town
Medium Ri	3	22	Male	Yes	Exposure B	180			4000	Bachelor	Bachelor	Orthodox	Georgian	City/Town
Low Risk	0	16	Male	Yes	Exposure B	90			3000	High Schoo	High Schoo	Orthodox	Armenian	City/Town
Low Risk	0	16	Female	Yes	Exposure B	30			3000	Master or	Bachelor	Orthodox	Georgian	City/Town
Medium Ri	5	19	Male	Yes	Exposure B	90			2000	Bachelor	High Schoo	Orthodox	Georgian	City/Town
Low Risk	1	19	Male	Yes	Exposure B	180			5000	Middle Sch	Middle Sch	Orthodox	Georgian	Emigrant (West)
Medium Ri	6	27	Male	Yes	Exposure B	360			3000	Middle Sch	High Schoo	Orthodox	Georgian	City/Town
High Risk	11	28	Male	No	Exposure B	90			1500	Bachelor	Bachelor	Orthodox	Mixed	City/Town
Low Risk	1	28	Male	Yes	Exposure B	90			3000	Bachelor	Bachelor	Orthodox	Mixed	City/Town
Medium Ri	5	18	Male	Yes	Exposure B	180			3000	Master or	Master or	Orthodox	Georgian	City/Town
Low Risk	2	26	Male	No	Exposure B	30			900	High Schoo	High Schoo	Orthodox	Georgian	City/Town
Low Risk	0	19	Female	No	Exposure B	90			3000	High Schoo	Middle Sch	Orthodox	Georgian	Village
Low Risk	0	25	Female	Yes	Exposure B	360			3000	Bachelor	High Schoo	Orthodox	Georgian	City/Town
Low Risk	1	30	Female	No	Exposure B	30			3500	High Schoo	High Schoo	Orthodox	Georgian	City/Town
Low Risk	0	20	Male	No	Exposure B	5			3000	Bachelor	Bachelor	Orthodox	Georgian	Village
Low Risk	1	18	Female	Yes	Exposure B	180			1000	High Schoo	Middle Sch	Orthodox	Georgian	City/Town

Low Risk	2	24	Male	Yes	Exposure B	50			3000	High Schoo	High Schoo	Muslim	Georgian	City/Town
Low Risk	1	30	Female	Yes	Exposure B	30			500	Bachelor	Bachelor	Orthodox	Georgian	City/Town
Low Risk	1	20	Male	Yes	Exposure B	30			1500	Bachelor	High Schoo	Orthodox	Georgian	City/Town
Low Risk	1	20	Male	Yes	Exposure B	30			3000	Bachelor	High Schoo	Orthodox	Georgian	City/Town
Low Risk	0	20	Male	No	Exposure B	30			5000	Master or	High Schoo	Orthodox	Georgian	City/Town
Low Risk	0	22	Female	Yes	Exposure B	90			5000	High Schoo	High Schoo	Orthodox	Georgian	Emigrant (West)
Medium Ri	6	16	Male	Yes	Exposure B	90			1500	Bachelor	Bachelor	Muslim	Georgian	City/Town
Low Risk	1	20	Male	No	Not Expose	30			5000	Master or	Bachelor	Orthodox	Georgian	City/Town
Low Risk	0	20	Male	No	Not Expose	30			5000	Master or	Bachelor	Orthodox	Georgian	City/Town
Low Risk	0	18	Male	Yes	Exposure B	13			5000	Bachelor	Master or	Orthodox	Georgian	City/Town
Low Risk	0	18	Male	Yes	Exposure B	13			5000	Bachelor	Master or	Orthodox	Georgian	City/Town
Low Risk	1	30	Female	No	Exposure B	30			1500	Bachelor	Bachelor	Orthodox	Georgian	Emigrant (West)
Low Risk	0	18	Male	Yes	Exposure B	17			5000	Bachelor	Master or	Orthodox	Georgian	City/Town
Low Risk	0	26	Female	No	Exposure B	30			1500	Bachelor	Bachelor	Orthodox	Georgian	City/Town
Low Risk	0	26	Male	Yes	Exposure B	45			18	Bachelor	Bachelor	Orthodox	Georgian	City/Town
Low Risk	1	19	Male	No	Exposure B	20			500	Bachelor	Bachelor	Orthodox	Georgian	City/Town
Medium Ri	5	30	Male	Yes	Exposure B	90			1500	Bachelor	Master or	Orthodox	Georgian	City/Town
Low Risk	0	30	Female	No	Exposure B	30			3000	Master or	Master or	Orthodox	Georgian	City/Town
Low Risk	1	18	Female	Yes	Exposure B	90			15000	Bachelor	Bachelor	Orthodox	Georgian	City/Town
Low Risk	0	30	Female	No	Exposure B	90			2300	Bachelor	Bachelor	Orthodox	Georgian	City/Town
Medium Ri	3	16	Female	No	Not Expose	0			1000	High Schoo	High Schoo	Orthodox	Georgian	City/Town
Low Risk	0	16	Female	Yes	Exposure B	30				Master or	Master or	Other	Georgian	Village
Low Risk	1	19	Male	No	Exposure B	30			6000	Bachelor	Bachelor	Orthodox	Georgian	City/Town
Low Risk	0	19	Male	Yes	Exposure B	60			2000	Bachelor	Bachelor	Orthodox	Georgian	City/Town
Low Risk	1	30	Female	No	Exposure B	180			1500	Middle Sch	High Schoo	Muslim	Georgian	City/Town
Low Risk	0	24	Male	No	Not Expose	150			1500	Bachelor	Bachelor	Orthodox	Georgian	City/Town
Low Risk	0	16	Female	No	Exposure B	60			2500	Bachelor	Bachelor	Orthodox	Georgian	City/Town
Low Risk	1	18	Male	Yes	Exposure B	30			2000	High Schoo	High Schoo	Orthodox	Georgian	Emigrant (West)
High Risk	13	16	Female	No	Not Expose	30				PhD	PhD	Other	Other	City/Town
Low Risk	1	16	Female	Yes	Exposure B	30			3000	High Schoo	High Schoo	Orthodox	Mixed	City/Town
Low Risk	2	16	Male	No	Not Expose	10			3000	High Schoo	High Schoo	Orthodox	Georgian	City/Town
Low Risk	1	23	Male	No	Exposure B	30			1500	High Schoo	Master or	Catholic	Georgian	City/Town
Low Risk	1	30	Male	Yes	Exposure B	360			1500	High Schoo	High Schoo	Orthodox	Georgian	City/Town
Low Risk	0	16	Female	Yes	Exposure B	10			1000	Bachelor	High Schoo	Orthodox	Georgian	Village
Medium Ri	3	23	Male	Yes	Exposure B	90			3000	Bachelor	High Schoo	Orthodox	Georgian	City/Town
Low Risk	1	18	Female	No	Exposure B	10			2000	High Schoo	High Schoo	Orthodox	Georgian	Village
Low Risk	2	16	Female	Yes	Exposure B	60			3000	High Schoo	High Schoo	Orthodox	Georgian	City/Town
High Risk	11	24	Male	Yes	Exposure B	60			2000	Bachelor	Bachelor	Orthodox	Georgian	City/Town
Low Risk	1	24	Male	Yes	Exposure B	90			4000	Bachelor	Bachelor	Orthodox	Georgian	City/Town
Low Risk	1	24	Male	No	Exposure B	90			4000	Bachelor	Bachelor	Orthodox	Georgian	City/Town
Medium Ri	4	24	Male	Yes	Exposure B	90			4000	Bachelor	Bachelor	Orthodox	Georgian	City/Town
Medium Ri	6	30	Female	Yes	Exposure B	360			1000	High Schoo	High Schoo	Orthodox	Georgian	City/Town
Low Risk	0	30	Male	Yes	Exposure B	90			5000	Master or	Bachelor	Orthodox	Georgian	City/Town
Low Risk	1	16	Female	Yes	Exposure B	20			3000	Bachelor	Bachelor	Muslim	Georgian	City/Town

Low Risk	2	23	Male	Yes	Exposure B	60			6000	Bachelor	Bachelor	Orthodox	Georgian	City/Town
Low Risk	0	16	Female	Yes	Exposure B	90			2000	Bachelor	High Schoo	Orthodox	Georgian	Village
High Risk	12	28	Male	Yes	Exposure B	90			600	High Schoo	Bachelor	Orthodox	Georgian	City/Town
Low Risk	0	20	Female	No	Exposure B	30			1500	Bachelor	Bachelor	Orthodox	Georgian	City/Town
Low Risk	0	26	Female	No	Exposure B	30			4000	Master or I	Bachelor	Orthodox	Georgian	City/Town
Low Risk	2	30	Female	Yes	Exposure B	60			1500	Bachelor	Master or I	Orthodox	Georgian	City/Town
Low Risk	1	28	Female	Yes	Exposure B	30			2000	Bachelor	Bachelor	Orthodox	Georgian	City/Town
Low Risk	0	30	Male	Yes	Exposure B	30			3000	Bachelor	Bachelor	Orthodox	Georgian	Emigrant (West)
Low Risk	1	18	Female	Yes	Exposure B	30			10000	Master or I	Bachelor	Orthodox	Georgian	City/Town
Low Risk	1	30	Male	Yes	Exposure B	180			3000	Master or I	Master or I	Orthodox	Georgian	City/Town
Low Risk	1	21	Female	Yes	Exposure B	90			1500	Bachelor	Bachelor	Orthodox	Georgian	Village
Low Risk	1	28	Female	Yes	Exposure B	180			1500	Middle Sch	High Schoo	Protestant	Georgian	City/Town
Low Risk	1	16	Male	Yes	Exposure B	30			1000	Bachelor	Bachelor	Orthodox	Georgian	City/Town
Low Risk	1	18	Male	Yes	Exposure B	180			3000	Bachelor	Bachelor	Orthodox	Georgian	City/Town
Low Risk	2	16	Male	No	Not Expose	20			3000	Bachelor	Bachelor	Orthodox	Georgian	City/Town
Medium Ri	3	17	Male	Yes	Exposure B	360			1500	Bachelor	Bachelor	Orthodox	Georgian	City/Town
Medium Ri	3	17	Male	Yes	Exposure B	360			1500	Bachelor	Bachelor	Orthodox	Georgian	Emigrant (West)
Low Risk	0	16	Male	Yes	Exposure B	20			3000	Bachelor	High Schoo	Orthodox	Georgian	City/Town
Low Risk	2	18	Male	No	Not Expose	20			1000	Bachelor	High Schoo	Orthodox	Georgian	City/Town
Medium Ri	3	18	Male	Yes	Exposure B	240			3500	Bachelor	Bachelor	Orthodox	Georgian	City/Town
Low Risk	0	30	Female	No	Exposure B	90			3000	Bachelor	Bachelor	Orthodox	Georgian	City/Town
Low Risk	1	17	Male	Yes	Exposure B	90			1500	Master or I	Master or I	Orthodox	Georgian	Emigrant (West)
Low Risk	0	30	Female	Yes	Exposure B	30			1500	High Schoo	Bachelor	Orthodox	Armenian	City/Town
Low Risk	0	16	Male	No	Not Expose	30			1000	High Schoo	High Schoo	Orthodox	Georgian	City/Town
Low Risk	0	27	Female	No	Exposure B	180			5000	Bachelor	Bachelor	Orthodox	Georgian	City/Town
Low Risk	0	18	Male	Yes	Exposure B	90			3000	Bachelor	Bachelor	Orthodox	Georgian	Village
Low Risk	0	30	Male	Yes	Exposure B	180			1500	High Schoo	High Schoo	Orthodox	Georgian	City/Town
Medium Ri	3	23	Male	No	Not Expose	180			3000	Bachelor	Bachelor	Orthodox	Georgian	City/Town
Low Risk	0	24	Female	Yes	Exposure B	10			4000	Middle Sch	Bachelor	Other	Mixed	City/Town
Low Risk	0	16	Female	Yes	Exposure B	240			2000	Master or I	Master or I	Orthodox	Georgian	City/Town
Low Risk	0	19	Female	Yes	Exposure B	10			1000	High Schoo	High Schoo	Other	Other	City/Town
Low Risk	0	19	Male	No	Not Expose	10			1500	Bachelor	Bachelor	Orthodox	Georgian	City/Town
Low Risk	0	16	Female	Yes	Exposure B	180			9000	Master or I	Master or I	Muslim	Georgian	Emigrant (West)
High Risk	8	24	Female	Yes	Exposure B	90			1500	Bachelor	Bachelor	Orthodox	Georgian	City/Town
Low Risk	0	30	Male	No	Exposure B	10			3000	Master or I	Bachelor	Orthodox	Georgian	Village
Medium Ri	3	19	Male	Yes	Exposure B	60			4000	Bachelor	Bachelor	Orthodox	Georgian	City/Town
High Risk	9	30	Male	No	Exposure B	90			2000	Master or I	High Schoo	Orthodox	Georgian	Village
Medium Ri	3	16	Male	Yes	Exposure B	30			2500	Bachelor	High Schoo	Protestant	Georgian	City/Town
Low Risk	0	16	Male	Yes	Exposure B	17			4000	Bachelor	Bachelor	Orthodox	Georgian	City/Town
Low Risk	0	22	Male	Yes	Exposure B	75			6000	Bachelor	High Schoo	Orthodox	Georgian	Emigrant (West)
Low Risk	0	24	Female	No	Not Expose	0			1500	Bachelor	High Schoo	Orthodox	Georgian	City/Town
Low Risk	0	30	Male	No	Exposure B	180			1500	High Schoo	High Schoo	Orthodox	Georgian	Emigrant (West)
Medium Ri	4	16	Male	No	Not Expose	0			0	Master or I	Master or I	Orthodox	Georgian	City/Town
Low Risk	0	25	Male	Yes	Exposure B	30			2000	Master or I	Bachelor	Orthodox	Georgian	City/Town

Low Risk	2	17	Female	Yes	Exposure B	30			6000	Master or I	Master or I	Orthodox	Georgian	City/Town
Low Risk	0	24	Male	Yes	Exposure B	180			1500	Bachelor	Bachelor	Orthodox	Georgian	Village
High Risk	8	30	Male	Yes	Exposure B	360			1000	High Schoo	High Schoo	Orthodox	Georgian	Emigrant (West)
Low Risk	0	22	Female	No	Not Expose	0			3000	Bachelor	Bachelor	Orthodox	Georgian	City/Town
Low Risk	1	16	Male	Yes	Exposure B	20			3000	High Schoo	High Schoo	Orthodox	Georgian	City/Town
Low Risk	2	24	Male	No	Not Expose	0			1000	High Schoo	High Schoo	Orthodox	Georgian	City/Town
Low Risk	0	16	Male	No	Exposure B	20			2000	Master or I	Bachelor	Orthodox	Georgian	City/Town
Low Risk	0	25	Male	Yes	Exposure B	60			3000	High Schoo	Bachelor	Orthodox	Georgian	City/Town
Low Risk	0	16	Female	Yes	Exposure B	90			2000	Bachelor	Bachelor	Orthodox	Georgian	City/Town
Low Risk	0	16	Male	No	Not Expose	0			500	High Schoo	High Schoo	Orthodox	Georgian	City/Town
Low Risk	0	25	Male	Yes	Exposure B	90			2000	High Schoo	Bachelor	Orthodox	Georgian	City/Town
Low Risk	0	26	Male	Yes	Exposure B	60			10000	Master or I	PhD	Orthodox	Georgian	Village
Low Risk	0	20	Male	No	Not Expose	10			1500	Bachelor	High Schoo	Orthodox	Georgian	City/Town
Medium Ri	5	16	Male	Yes	Exposure B	90			1500	High Schoo	High Schoo	Orthodox	Georgian	Emigrant (West)
Medium Ri	3	30	Male	No	Exposure B	30			300	Bachelor	Middle Sch	Orthodox	Georgian	City/Town
Medium Ri	4	18	Male	No	Not Expose	10			1000	Bachelor	High Schoo	Orthodox	Georgian	City/Town
Low Risk	0	28	Female	Yes	Exposure B	90			5000	Bachelor	Bachelor	Orthodox	Georgian	City/Town
Low Risk	0	19	Male	Yes	Exposure B	30			5000	High Schoo	Master or I	Orthodox	Georgian	City/Town
Medium Ri	7	30	Male	Yes	Exposure B	30			1000	High Schoo	High Schoo	Orthodox	Georgian	Village
Medium Ri	4	29	Female	No	Exposure B	60			7100	Bachelor	Bachelor	Orthodox	Georgian	City/Town
Low Risk	0	30	Male	Yes	Exposure B	180			1000	Bachelor	High Schoo	Orthodox	Georgian	Village
Low Risk	1	16	Female	No	Not Expose	30			4000	Bachelor	High Schoo	Orthodox	Georgian	City/Town
Low Risk	0	24	Male	Yes	Exposure B	30			1000	High Schoo	High Schoo	Other	Georgian	City/Town
Low Risk	0	29	Female	No	Exposure B	360			1500	Bachelor	Bachelor	Orthodox	Georgian	City/Town
Low Risk	0	16	Male	Yes	Exposure B	30			3000	Bachelor	High Schoo	Orthodox	Georgian	City/Town
Low Risk	0	24	Female	No	Exposure B	90			4000	Bachelor	Bachelor	Orthodox	Georgian	City/Town
Low Risk	0	30	Male	Yes	Exposure B	30			1500	Bachelor	High Schoo	Muslim	Georgian	Mountainous Region
Low Risk	1	30	Male	Yes	Exposure B	45			3000	Master or I	Master or I	Orthodox	Georgian	City/Town
Low Risk	1	30	Female	Yes	Exposure B	30			3000	Bachelor	Bachelor	Orthodox	Georgian	City/Town
Low Risk	1	25	Male	Yes	Exposure B	60			3000	Bachelor	Middle Sch	Orthodox	Georgian	City/Town
High Risk	10	30	Male	No	Exposure B	360			4100	Bachelor	High Schoo	Other	Georgian	City/Town
