# Supplementary material for: Screen time and autism like behavior: Cross-sectional study from Georgia
Source: Prev Med Rep. 2026 May 22;67:103503. doi: 10.1016/j.pmedr.2026.103503 (PMC13223515; doi:10.1016/j.pmedr.2026.103503)
Supplement: Supplementary material 2 — General Linear Model Results for Factors Associated With Modified Checklist for Autism in Toddlers, Revised With Follow-Up (M-CHAT-R/F) Scores Among Children Aged 16–30 Months in Georgia, April 2025–January 2026. [file mmc4.docx]

**Supplementary File 4. General Linear Model Results for Factors Associated With Modified Checklist for Autism in Toddlers, Revised With Follow-Up (M-CHAT-R/F) Scores Among Children Aged 16–30 Months in Georgia, April 2025–January 2026**

| **General linear model: biologic sex, start of screen time exposure, religious belonging of the family, and place of residence** | | | | | | | | |
| --- | --- | --- | --- | --- | --- | --- | --- | --- |
| **Dependent Variable: M-CHAT-R/F Score** | | | | | | | | |
| **Source** | **Type III Sum of Squares** | **df** | **Mean Square** | **F** | **P** | **Partial Eta Squared** | **Noncent. Parameter** | **Observed power** |
| Corrected Model | 1728.810a | 103 | 16.79 | 2.37 | < 0.01 | 0.13 | 244.51 | 1.00 |
| Intercept | 202.983 | 1 | 202.98 | 28.71 | < 0.01 | 0.02 | 28.71 | 1.00 |
| Biologic sex | 32.736 | 1 | 32.74 | 4.63 | 0.03 | 0.00 | 4.63 | 0.57 |
| Start of screen time exposure | 91.339 | 4 | 22.84 | 3.23 | 0.01 | 0.01 | 12.92 | 0.83 |
| Religious belonging of the family | 61.065 | 5 | 12.21 | 1.73 | 0.13 | 0.01 | 8.64 | 0.60 |
| Place of residence | 12.237 | 4 | 3.06 | 0.43 | 0.79 | 0.00 | 1.73 | 0.15 |

| **General linear model: biologic sex, start of screen time exposure, and parental education** | | | | | | |
| --- | --- | --- | --- | --- | --- | --- |
| **Dependent Variable: M-CHAT-R/F Score** | | | | | | |
| **Source** | **Type III Sum of Squares** | **df** | **Mean Square** | **F** | **p** | **Partial Eta Squared** |
| Corrected Model | 2755.49 | 160 | 17.22 | 2.57 | < 0.01 | 0.20 |
| Intercept | 856.68 | 1 | 856.68 | 127.87 | < 0.01 | 0.07 |
| Biologic sex | 26.40 | 1 | 26.40 | 3.94 | 0.05 | 0.00 |
| Start of screen time exposure | 229.94 | 4 | 57.48 | 8.58 | < 0.01 | 0.02 |
| Level of education (mother) | 162.98 | 4 | 40.74 | 6.08 | < 0.01 | 0.02 |
| Level of education (father) | 7.55 | 4 | 1.88 | 0.28 | 0.89 | 0.00 |

| **General linear model: start of screen time exposure and parental education** | | | | | | |
| --- | --- | --- | --- | --- | --- | --- |
| **Dependent Variable: M-CHAT-R/F Score** | | | | | | |
| **Source** | **Type III Sum of Squares** | **df** | **Mean Square** | **F** | **p** | **Partial Eta Squared** |
| Corrected Model | 6117.226a | 515 | 11.89 | 2.00 | < 0.01 | 0.45 |
| Intercept | 862.196 | 1 | 862.20 | 145.19 | < 0.01 | 0.10 |
| Start of screen time exposure | 159.668 | 4 | 39.92 | 6.72 | < 0.01 | 0.02 |
| Level of education (mother) | 62.858 | 4 | 15.71 | 2.65 | 0.03 | 0.01 |
| Level of education (father) | 22.709 | 4 | 5.68 | 0.96 | 0.43 | 0.00 |

| **General linear model: start of screen time exposure, parental education, and average daily screen time duration for the past 6 months** | | | | | | |
| --- | --- | --- | --- | --- | --- | --- |
| **Dependent Variable: M-CHAT-R/F Score** | | | | | | |
| **Source** | **Type III Sum of Squares** | **df** | **Mean Square** | **F** | **p** | **Partial Eta Squared** |
| Corrected Model | 5467.85 | 492 | 11.11 | 1.76 | < 0.01 | 0.47 |
| Intercept | 753.07 | 1 | 753.07 | 119.29 | < 0.01 | 0.11 |
| Start of screen time exposure | 50.20 | 4 | 12.55 | 1.98 | 0.09 | 0.01 |
| Level of education (mother) | 192.39 | 4 | 48.10 | 7.62 | < 0.01 | 0.03 |
| Level of education (father) | 7.32 | 4 | 1.83 | 0.29 | 0.89 | 0.00 |
| Average daily screen time duration (past 6 months) | 788.12 | 52 | 15.15 | 2.40 | < 0.01 | 0.11 |

| **General linear model: start of screen time exposure, parental education, and average daily screen time duration for the past year** | | | | | | |
| --- | --- | --- | --- | --- | --- | --- |
| **Dependent Variable: M-CHAT-R/F Score** | | | | | | |
| **Source** | **Type III Sum of Squares** | **df** | **Mean Square** | **F** | **Sig.** | **Partial Eta Squared** |
| Corrected Model | 5476.976a | 541 | 10.12 | 1.55 | < 0.01 | 0.48 |
| Intercept | 701.170 | 1 | 701.17 | 107.10 | < 0.01 | 0.11 |
| Start of screen time exposure | 98.505 | 4 | 24.63 | 3.76 | 0.01 | 0.02 |
| Level of education (mother) | 69.732 | 4 | 17.43 | 2.66 | 0.03 | 0.01 |
| Level of education (father) | 57.041 | 4 | 14.26 | 2.18 | 0.07 | 0.01 |
| Average daily screen time duration (past year) | 862.367 | 66 | 13.07 | 2.00 | < 0.01 | 0.13 |
